# A bi-filtering method for processing single nucleotide polymorphism array data improves the quality of genetic map and accuracy of quantitative trait locus mapping in doubled haploid populations of polyploid *Brassica napus*

**DOI:** 10.1186/s12864-015-1559-4

**Published:** 2015-05-28

**Authors:** Guangqin Cai, Qingyong Yang, Bin Yi, Chuchuan Fan, Chunyu Zhang, David Edwards, Jacqueline Batley, Yongming Zhou

**Affiliations:** National Key Laboratory of Crop Genetic Improvement, Huazhong Agricultural University, Wuhan, 430070 China; Key Laboratory of Rapeseed Genetics and Breeding of Agriculture Ministry of China, Huazhong Agricultural University, Wuhan, 430070 China; School of Agriculture and Food Sciences, University of Queensland, St Lucia, QLD Australia

**Keywords:** *Brassica napus*, Polyploid, SNP array, Bi-filtering analysis, Genetic map, QTL mapping

## Abstract

**Background:**

Single nucleotide polymorphism (SNP) markers have a wide range of applications in crop genetics and genomics. Due to their polyploidy nature, many important crops, such as wheat, cotton and rapeseed contain a large amount of repeat and homoeologous sequences in their genomes, which imposes a huge challenge in high-throughput genotyping with sequencing and/or array technologies. Allotetraploid *Brassica napus* (AACC, 2n = 4x = 38) comprises of two highly homoeologous sub-genomes derived from its progenitor species *B. rapa* (AA, 2n = 2x = 20) and *B. oleracea* (CC, 2n = 2x = 18), and is an ideal species to exploit methods for reducing the interference of extensive inter-homoeologue polymorphisms (mHemi-SNPs and Pseudo-simple SNPs) between closely related sub-genomes.

**Results:**

Based on a recent *B. napus* 6K SNP array, we developed a bi-filtering procedure to identify unauthentic lines in a DH population, and mHemi-SNPs and Pseudo-simple SNPs in an array data matrix. The procedure utilized both monomorphic and polymorphic SNPs in the DH population and could effectively distinguish the mHemi-SNPs and Pseudo-simple SNPs that resulted from superposition of the signals from multiple SNPs. Compared with conventional procedure for array data processing, the bi-filtering method could minimize the pseudo linkage relationship caused by the mHemi-SNPs and Pseudo-simple SNPs, thus improving the quality of SNP genetic map. Furthermore, the improved genetic map could increase the accuracies of mapping of QTLs as demonstrated by the ability to eliminate non-real QTLs in the mapping population.

**Conclusions:**

The bi-filtering analysis of the SNP array data represents a novel approach to effectively assigning the multi-loci SNP genotypes in polyploid *B. napus* and may find wide applications to SNP analyses in polyploid crops.

**Electronic supplementary material:**

The online version of this article (doi:10.1186/s12864-015-1559-4) contains supplementary material, which is available to authorized users.

## Background

Oilseed rape (*Brassica napus* L., AACC, 2n = 38) is one of the most important oil crops in the world, which provides not only edible oil but also raw materials for bio-energy applications. *B. napus* is an allotetraploid that was generated from the natural hybridization of its two progenitor diploids of *Brassica rapa* (AA, 2n = 20) and *Brassica oleracea* (CC, 2n = 18) about 7,500 years ago [1,2]. *B. rapa* and *B. oleracea* were produced by extensive triploidization of their ancestral species at the genomic level [3-5]. The *B. napus* two subgenomes A_n_ and C_n_ are largely collinear (93%) to the corresponding diploid A_r_ (*B. rapa*) and C_o_ (*B. oleracea*) genomes [2]. The three species are believed to share a common ancestor with *Arabidopsis thaliana* [2,4-6]. Thus, on average, one ortholog Arabidopsis gene can find about four homologous copies in the *B. napus* genome [2,4,5,7]. Most orthologous gene pairs in *B. rapa* and *B. oleracea* remain as homoeologous pairs in *B. napus* A_n_ and C_n_ subgenomes (An ortholog gene in the A_n_ genome in most cases has a highly homologous copy of the sequence in the C_n_ genome) [2,5].

Single nucleotide polymorphism (SNP) markers have a wide range of applications in the construction of genetic maps, mapping and cloning of quantitative trait locus (QTL), linkage analysis, molecular marker-assisted selection (MAS), and molecular breeding of crops [8-12]. Edwards *et al.* and Hayward *et al.* estimated that there was a SNP in every 600 bp of the *B. napus* genome, for a total of approximately 1.7 million SNPs [13,14]. Westermeier *at al.* and Durstewitz *et al.* identified 87 and 604 SNPs in *B. napus* using an amplicon sequencing method, respectively [15,16]. Recently, Trick *et al.* identified 23,330 and 41,593 SNPs in the two cultivars, Ningyou7 and Tapidor using Solexa transcriptome sequencing [17-19]. Bus *et al.* identified more than 20,000 SNPs in 8 *B. napus* inbred lines using a next-generation restriction-site associated DNA (RAD) sequencing method [20]. A total of 7,322 genic SNPs were selected from publically available information for Illumina Infinium genotyping by Delourme *et al.* [21], and a ultrahigh-density SNP bin map containing 8,780 SNPs was constructed using a modified ddRADseq technique for two *B. napus* inbred lines and their 91 doubled haploid (DH) progenies [22]. Several methods have been used to successfully genotype *B. napus* with SNP markers, including mini-sequencing [15], Illumina GoldenGate genotyping [16], SNaPshot [23,24], Invader® [25] assays and SNAP primer amplification [7]. Recently, high-throughput 6K and 60K SNP arrays for *B. napus* based on the Illumina Infinium HD Assay have been developed, and used for QTL mapping [26,27], genome-wide association study [28], and genome structure analysis [29,30].

Compared with diploid species, such as rice [12,31], maize [32], tomato [33], chickpea [34], sorghum [35], and apple [36], the large-scale identification of SNPs and genotyping in *B. napus* faces more challenges due to the species’ complex genome structure [2,37,38]. For instance, SNP identification by transcriptome sequencing or using known EST sequence data showed that approximately 90% of identified SNP loci correspond to hemi-SNPs [17], resulting in a large number of heterozygous signals in genotyping analyses with SNP arrays [16,38]. Because the generation of heterozygous signals is due mainly to the binding of the SNP probe to two or more different genomic sequences (i.e., non-specific binding), the detected signal may not represent the genotype corresponding to the SNP probe itself. Two traditional solutions to this problem are (i) either to remove the SNPs with the heterozygous signals from further analysis, or (ii) to code signals with the same P1 value as the A genotype, those with the same P2 value as the B, and those with a non-parental value, as missing values (recorded as “-”) in a segregating population [26,27]. The first method will result in a low usage of the SNP array data, while the premise of the second method is the uniqueness of the binding site for the SNP probe in the genome. However, it is difficult for a considerable number of probes to meet this requirement in the *B. napus* genome [2,4,5], which leads to improper utilization of SNP data in many cases.

Different parameters have been proposed for quality evaluation of SNP arrays in diploid species (e.g. rice, maize and apple). For instance, the cluster separation score (CSS) is often used to select high-quality SNP probes (CSS > 0.3). However, the CSS is not always suitable for the determination of SNP loci [39,40], as it only describes the degree of separation between two homozygous versus heterozygous clusters, rather than the separation between two homozygous clusters [39,40]. For the populations consisting of pure individuals, such as DH lines and recombinant inbreeding lines (RIL), probes with a high heterozygous proportion (>5%) and low minor allele frequencies (MAF < 0.01) may be filtered out, and materials/lines with high missing data (>20%) or low call rate (<0.7) may be discarded from further analysis [36,40-44].

Due to the high frequency of multi-loci SNPs (hemi-SNP) in polyploid species, such as *B. napus*, it is not suitable to simply apply the parameters and criterions developed in the diploid species to evaluating the quality of SNP array probes in polyploid species. Therefore, there is a need for development of effective procedures to assess the quality of the SNP array probes and to make full use of SNP genotyping data in polyploid species.

In this study, a 6K SNP array (Illumina Infinium HD Assay) [27] for *B. napus* was applied to genotyping a DH population and its parents [30]. A procedure, called bi-filtering analysis was developed to improve the efficiency and accuracy of SNP array data analysis. The procedure firstly calculates the percentage of non-parental genotypes (PNPG), based on monomorphic loci, in a segregating population. Subsequently, the difference in PNPG of single-locus SNPs (Simple SNP and sHemi-SNP) and multi-loci SNPs (mHemi-SNP and Pseudo-simple SNP) was compared to filter multi-loci SNPs among the SNP loci and unauthentic lines in the DH population. Such a bi-directional filtering can optimize the population and eliminate multi-loci SNP interference, thus improving the quality of genetic map and accuracy of QTL mapping in polyploid *B. napus*.

## Methods

### Plant materials, field trails and trait evaluation

The HJ DH population was produced from microspore culture of F1 buds of the cross between Huashuang 5 (Hua5), a semi-winter type *B. napus* variety, and J7005, a winter-type *B. napus* pure line. The two parents were purified by microspore culture before hybridization. Detailed information about this population was described in Wu *et al*. [45] and Cai *et al*. [30].

The DH lines, together with their parental lines, F1 and RF1 hybrids were grown in a semi-winter rapeseed crop area, Wuhan in 2009–2010, 2010–2011, Huanggang in 2010–2011, and a spring rapeseed crop area Gansu in 2011, respectively. The field experiment followed a randomized complete block design with three replications. Each line was planted in two rows and 10–11 plants were maintained in each row, with a distance of 17 cm between plants within each row and 30 cm between rows. The parental line Hua5 was grown in every 20 lines as a control. The field management followed essentially regular breeding practice.

### Molecular marker and SNP array genotyping

Primer sequences for the SSR markers used for genetic mapping were described by Fan *et al.* [46] and the sequence information of all SSR markers is provided by Cai *at al.* [30].

The genotyping of SNPs was performed using a 6K Illumina Infinium HD Assay SNP array of *B. napus* (Illumina Inc., San Diego, CA) developed by the University of Queensland. The SNP genotyping was conducted following the instructions from Infinium HD Assay Ultra Protocol Guide (http://www.illumina.com/). All the SNP array data were clustered and visualized for further analysis using the Illumina GenomeStudio software (Illumina Inc., San Diego, CA). Each SNP was re-checked manually to determine if any error was observed during the clustering analysis. Detailed information about SNP array genotyping and data processing was described in Cai *et al*. [30].

### Construction of linkage map and QTL mapping

The method for genetic linkage map construction was described by Cai *et al.* [30]. QTLs were detected using the composite interval mapping (CIM) procedure with the software QTL Cartographer V2.5 [47]. A significance threshold for QTL at the level P =0.05 was determined through permutation analysis using 1000 repetitions. The other parameters and methods for QTL mapping were as described by Feng *et al.* [48].

## Results and Discussion

### Majority of polymorphic SNP loci exhibited heterozygous signals in *B. napus*

**Figure 1:**
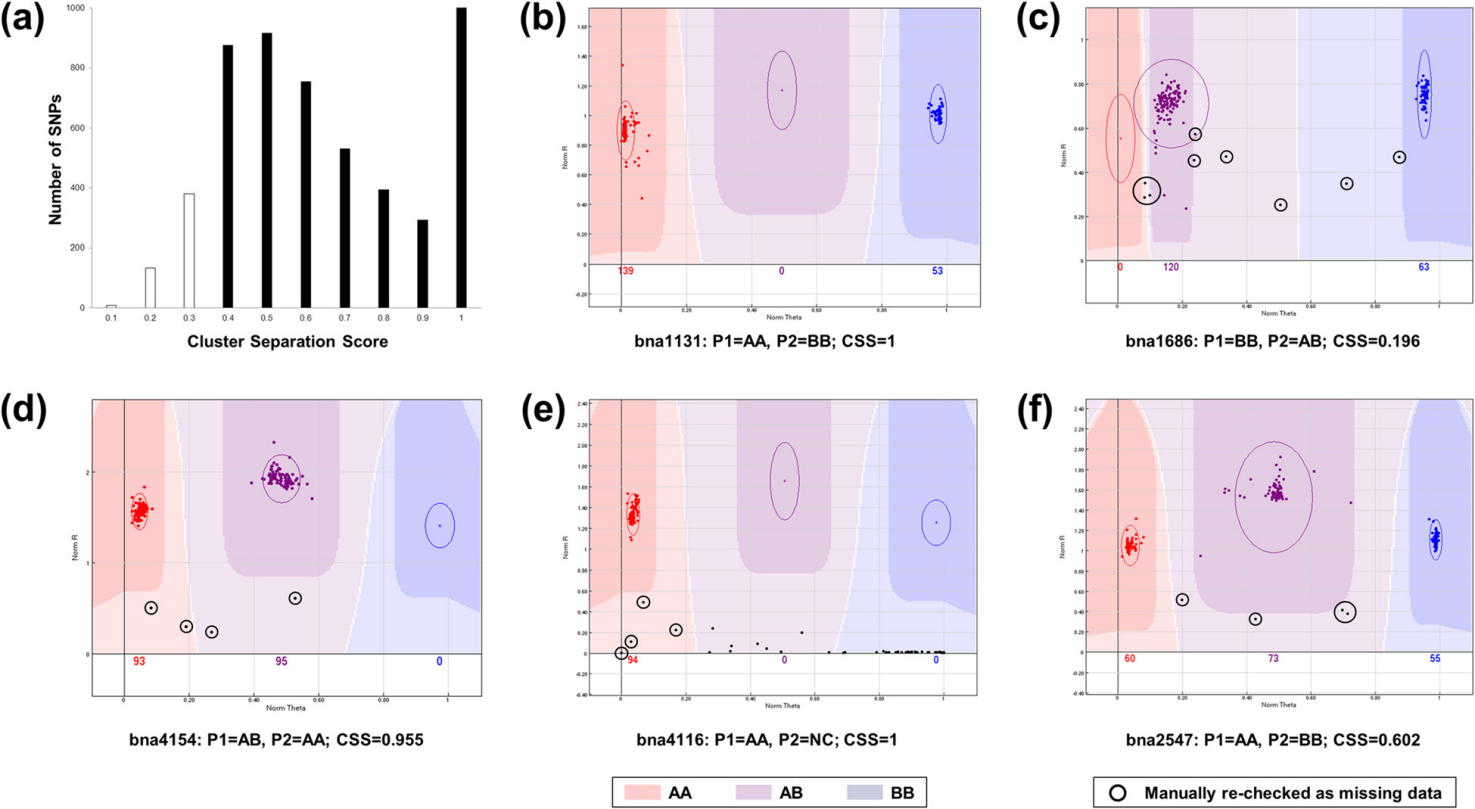
Different types of single nucleotide polymorphism (SNP) probes as clustered by GenomeStudio software in the HJ-DH population. **(a)** Distribution of the cluster separation score (CSS) for all 5306 SNPs; **(b)**-**(f)** Scoring of SNP genotyping data from different types of SNP probes. The three highlighted clusters denote the areas where the three different genotypes of homozygous allele AA (red), heterozygous AB (purple) and homozygous allele BB (blue) are called. Allele calls that are ambiguously located in the lighter colored areas between or below these areas are scored as “no call” (NC). Ellipses are used to mark the positions of the cluster calling areas. The dots with black circles are calls that needed to be manually re-checked and re-scored to missing data (“-”). **(b)** Typical score from probe bna1131 with two expected homozygous clusters (AA and BB); **(c)** The score from the SNP bna1686 with CSS <0.3 but with two clear parental genotype clusters; **(d)** The score from the SNP bna4154 with one parent being heterozygous (AB); **(e)** The score from the SNP bna4116 with one parent being NC; **(f)** The score from the SNP bna2547 with obvious 3 clusters (AA, AB, BB) in which the non-parental genotype of AB cannot be re-clustered to any homozygous cluster manually.

Previously, the HJ-DH population and its parental lines were genotyped with SSR markers and the 6K SNP arrays for *B. napus* [29,30] and the call rate of all 5,306 SNP loci on the array for all 192 samples was >0.7 [30]. There were 578 probes (10.9%) that were detected in less than 80% of samples and thus not included in further analysis. The remaining 4,728 SNPs were used for cluster analysis using GenomeStudio software [30]. Among the 4,728 SNPs, 521 (11%) had a CSS <0.3 (Figure 1a). As doubled haploids, all the DH lines should have only their parental genotypes with two expected homozygous clusters (AA and BB, Figure 1b). However, other types of genotyping data were observed after clustering, including SNPs with CSS <0.3 but with clear clusters (Figure 1c), SNPs with one of the parental genotype being heterozygous (Figure 1d) or not detected (no call, Figure 1e), and SNPs with a high frequency of non-parental genotype (NPG, i.e. the genotype in a SNP locus of a DH line different from any one of two parental lines) in the progeny population (Figure 1f). There were 155 polymorphic SNPs out of the 521 SNPs with CSS <0.3, most of which had clear clusters (Figure 1c). After a check of all the calls manually, the SNPs between two clusters were rescored as missing data (“-”, Figure 1).

Among the 1,850 polymorphic loci out of the 4728 probes (39.1%) [30], 1,149 (62%) were detected as heterozygous signals in one parent (Table 1). There were also 1,005 SNPs (54.3%) that had three clear clusters with at least 1 DH lines per cluster in the DH population (Table 1). Those two types (heterozygous SNPs and SNPs with three clear clusters in the DH population) calls in SNP arrays for diploid species would frequently be discarded [40-44]. If a similar treatment was followed in this study, there would be only 158 polymorphic SNP loci with non-heterozygous calls and two clearly parental clusters in the population left for further genotyping analysis (Table 1), only accounting for 3.0% SNPs on the array. Such a choice will significantly compromise the high-throughput property of SNP arrays.

**Table 1:** **Types and numbers of the polymorphism combinations in two parental lines**

**Homologous signals**				**Homologous/heterozygous signals**		
**Hua5**	**J7005**	**N** ^**a**^		**Hua5**	**J7005**	**N**
AA	BB	262 (234)		AA	AB	240 (63)
AA	NC	44 (10)		BB	AB	313 (165)
BB	AA	264 (242)		AB	AA	209 (44)
BB	NC	78 (39)		AB	BB	358 (173)
NC	AA	27 (7)		AB	NC	11 (8)
NC	BB	26 (11)		NC	AB	18 (9)
**Total**		**701 (543)**				**1149 (462)**

^a^The number of the SNP loci. The number in the bracket designates the SNP locus number that exhibits segregation of three clear clusters (a cluster at least had one DH line) in the DH population.

Above results revealed that near 40% SNPs were polymorphic between two parental lines, consistent with findings in maize and other crops [32,40]. However, data from monomorphic loci accounting for a large portion of the SNP array were directly discarded in previous studies [26,27,30], resulting in a potential loss of information from both the array and genotyped samples.

### Monomorphic SNP loci can be used to genotype the mapping population and assess SNP detection errors

**Figure 2:**
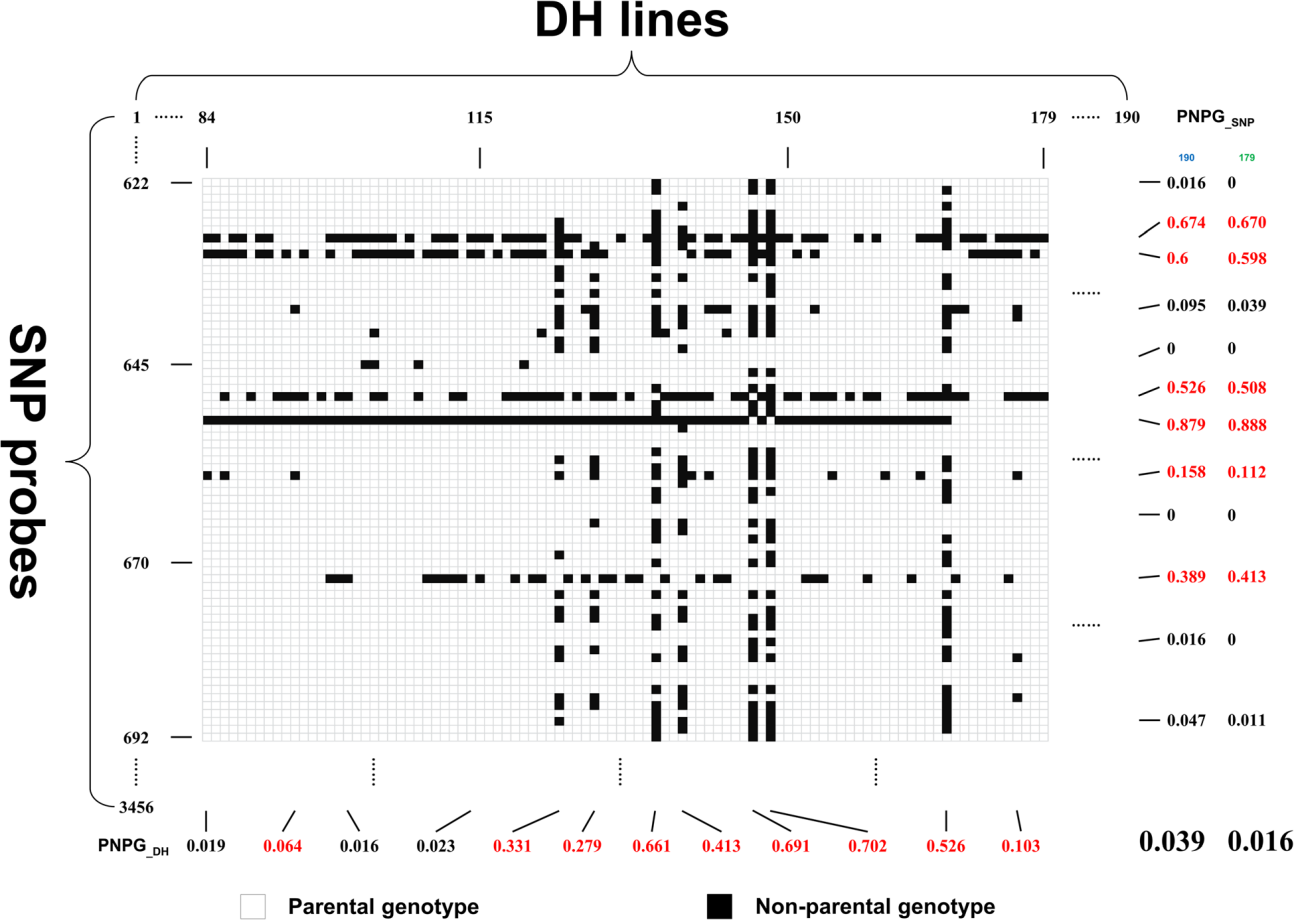
Schematic diagram of a two-dimensional matrix for analyzing monomorphic single nucleotide polymorphisms (SNPs) in the HJ-DH population. The matrix lists the genotypes of each SNP locus in all doubled haploid (DH) lines horizontally and the genotypes of each DH line in all SNP loci vertically. The blank and black squares represent the parental genotype and non-parental genotype in the population, respectively. The PNPG__SNP_ and PNPG__DH_ are calculated by the formulas as described in the Results and Discussion section.

Because the genotype of a given locus in each DH line can be inferred according to their parental genotypes in theory, we hypothesized that the monomorphic SNP loci could be used to evaluate the authenticity of each DH line, as well as the stability and error in the SNP array detection. For that purpose, a two-dimensional matrix was established to genotype the individual lines of the DH population (Figure 2, Additional file 1: Table S1). This matrix listed the genotypes of each SNP locus in all DH lines horizontally and the genotypes of each DH line in all SNP loci vertically. In such a matrix, the occurrence of a NPG might be due to an error in the SNP detection system or due to the DH line itself (e.g. mechanical or biological contamination in the sample). The quantification of the percentage of non-parental genotypes (PNPG) in these SNPs for each DH line in the vertical direction can accurately identify the authenticity of each DH line in the population. After removing the potentially unauthentic DH lines, the remaining differences can be used to assess the reliability and stability of SNP detection in the array.

For the DH lines listed in the vertical direction, the PNPG of DH lines (PNPG__DH_) can be calculated with the following formula:

$$ PNP{G}_{\_DH}=\frac{1-P{G}_j}{M_j} $$

Where PNPG__DH_ represents the percentage of NPGs in all detected SNP loci of the j^th^ DH line, PG_j_ represents the number of parental genotypes (PGs) in all SNP loci of the j^th^ line, and M_j_ represents the number of detected SNP loci of the j^th^ line.

For a true DH line, the PNPG value should theoretically be zero. However, many factors such as genetic mutations, the stability of the SNP detection system, and the mechanical or biological contamination of the samples, can affect genotyping results. However, the probability that all of these factors will have a significant impact on the genotyping results is small. Based on the above considerations, in the subsequent analysis, PNPG =0.05 was set as a threshold value to determine the authenticity of a given DH line.

**Figure 3:**
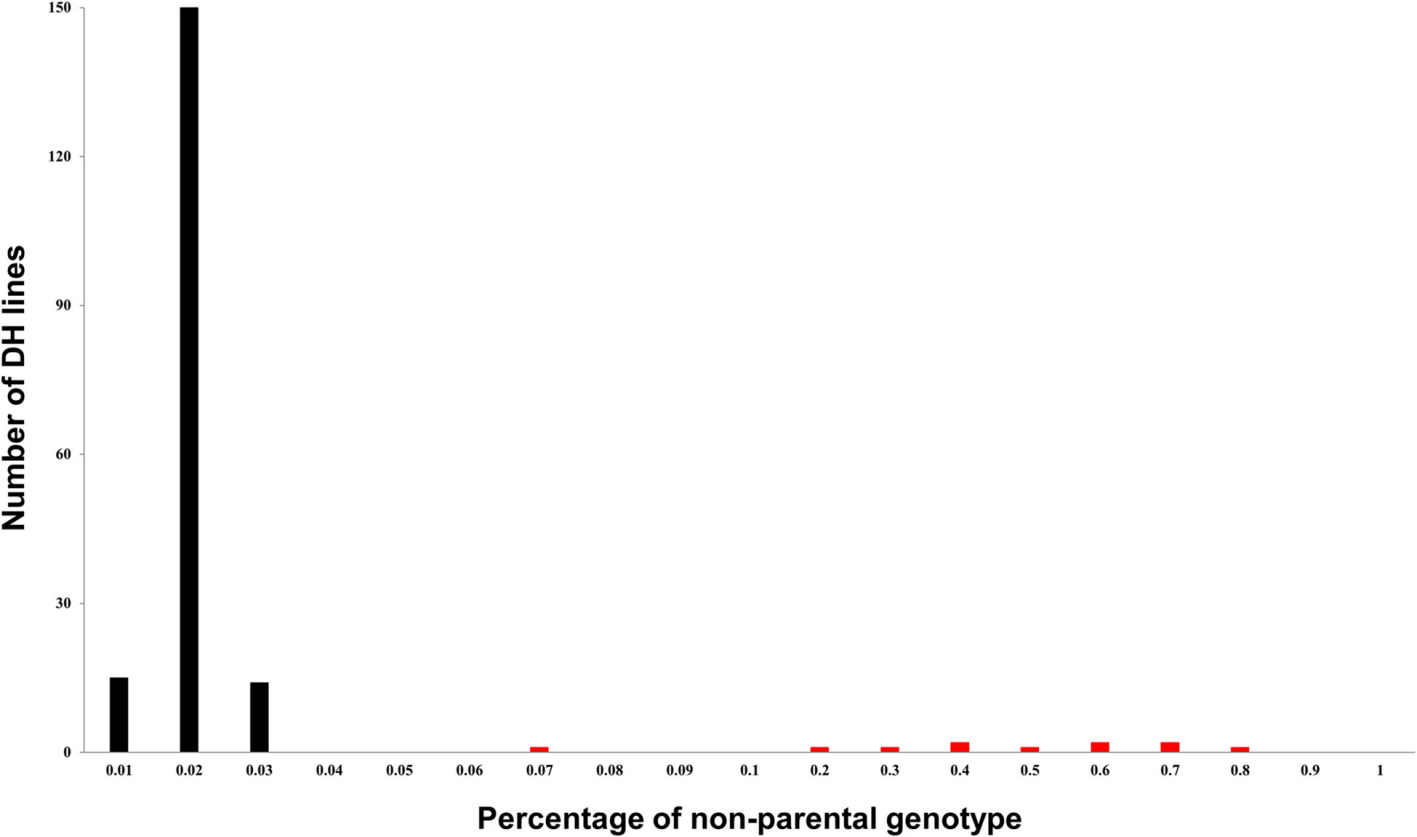
Frequency of the percentage of non-parental genotype (PNPG) measured by monomorphic single nucleotide polymorphisms (SNPs) in the HJ-DH population. The PNPG of each doubled haploid (DH) line is calculated by the formula as described in the Results and Discussion section.

The PNPG values were calculated for each DH line based on 3456 monomorphic SNP loci, including the SNPs that had more than 20% of missing data (Additional file 1: Table S1). The average PNPG for the population was 0.039, with a PNPG <0.03 in 179 lines and >0.05 in 11 lines (Figure 3, Additional file 1: Table S1). After removing these 11 lines, the average PNPG for the DH population decreased to 0.016. The remaining 179 DH lines were considered to be the true genetic offspring of the two parents and used for the subsequent analysis. We also checked the genotypes of these 11 unauthentic DH lines based on the 473 polymorphic SSR loci, and could not find the abnormity and error. One might consider a similar examination with monomorphic SSR markers. However, it seems only feasible to apply monomorphic SNPs for such a purpose, since monomorphic SSR markers normally are no longer used for genotyping of a segregation population in a regular SSR genotyping experiment, and the throughput of SSR genotyping is obviously much lower than that of SNP. In this regard, the high-throughput SNPs are more powerful in evaluating the authenticity of the DH population offspring than other regular markers. The above results showed that the PNPG value of a DH line could be used in the evaluation of the structure of the population and the authenticity of the offspring. After excluding the unauthentic DH lines from the population, the stability and error of the SNP array detection system could be further assessed.

**Figure 4:**
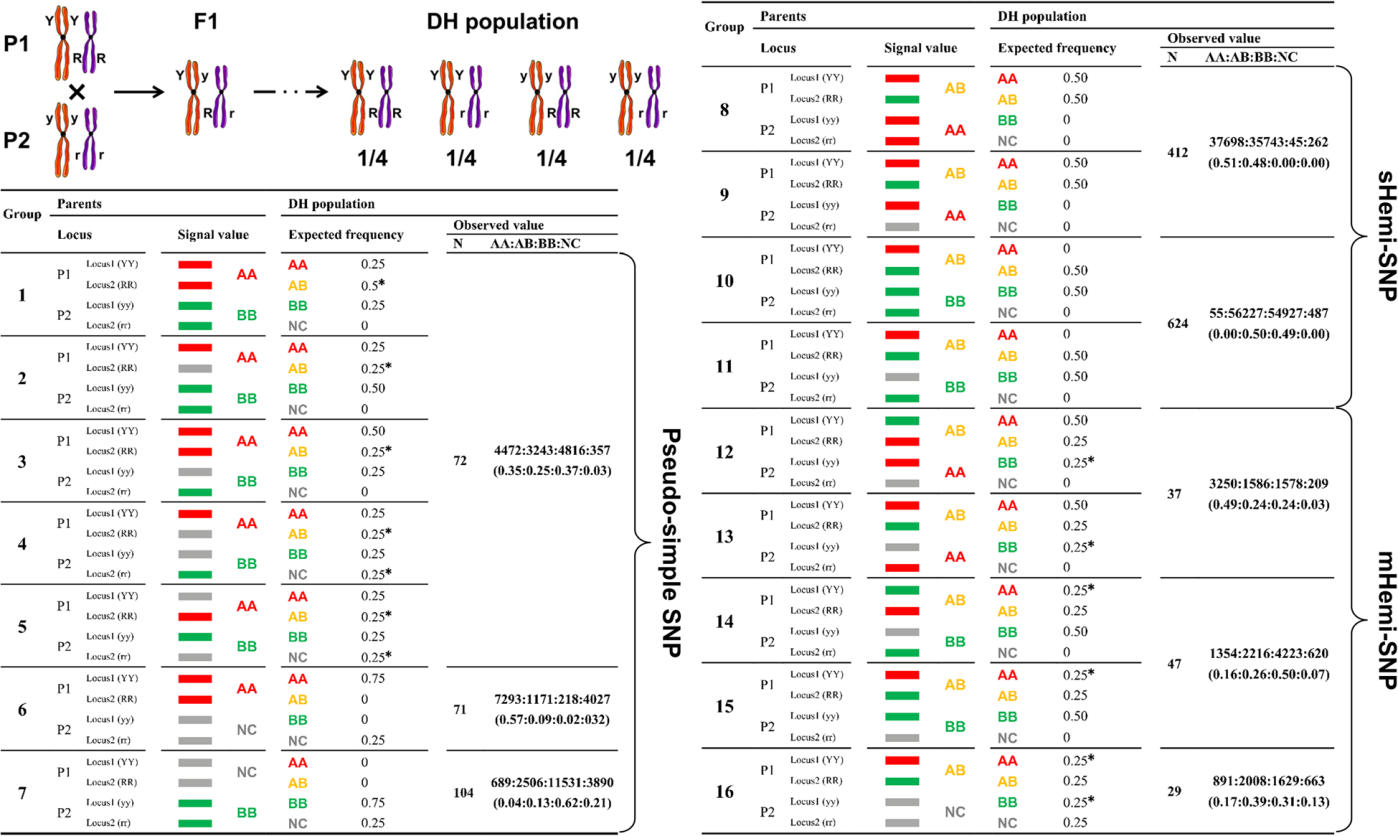
Possible genotypes derived from inter-homoeologues targeted by a given SNP probe and their frequency in the HJ-DH population. Considering the two inter-homoeologous sequences YY and RR in P1 and their alleles yy and rr in P2 as two independent loci in the genome, the DH population will expect four genotypes of YYRR, YYrr, yyRR, and yyrr with a frequency of 1/4 for each (top left). Fluorescence signals of the parental lines are assigned as AA (C/G base, red), BB (A/T base, green) and AB (heterozygosis, orange), respectively. In the case of a null locus, the miss signal is assigned as NC (grey). In Pseudo-simple SNP type (lower left), only a same set of signals as Simple SNP are detected in the parental lines but there will be non-parental genotypes (NPGs) segregation in the DH population in Group 1-5. In sHemi-SNP type, there will be AB (heterozygous) signal detected due to presence of hemi-SNP but there will no NPG in the DH population (top right). In mHemi-SNP type, the signal values are similar to sHemi-SNP in parental lines but there will be NPG signals detected in the DH populations due to multiple mismatched nucleotides within the inter-homoeologous sequences (lower right). The color bars can be used for calculation of signal values in the DH population (expected frequency). “*” marks the NPGs occurred in the DH population. N is the number of the polymorphic SNPs in indicated group(s). The numbers of each signal for corresponding SNP group in the 179 DH lines are listed in the column of AA:AB:BB:NC. The number in the bracket refers to the ratio for each signal (genotype).

Due to the existence of a large number of inter-homoeologues in the A and C subgenomes of *B. napus* [17,49], it is difficult to ensure that a SNP probe only bind to a particular genomic sequence/region when designing SNP probes. Such a lack of specificity could result in a large number of heterozygous signals in SNP detection in *B. napus*. In this study, 62% of the detected SNPs were loci with heterozygous signals in one of the parents (Table 1), although both the parental lines were homozygous (doubled haploids through microspore culture). Such a non-specific binding of SNP probes and the consequent heterozygous signals in the array analysis could result in the appearance of the NPGs in the DH population. To test the hypothesis and to understand the cause of the heterozygous signals in the SNP array, we took a similar procedure as described by Trick *et al.* [17], in which an unambiguous allelic SNP was termed “Simple SNP” and the allelic polymorphisms due to the presence of homoeologous sequences “Hemi-SNP” [17]. In such a way, we classified SNPs into Simple SNP, Hemi-SNP, and Pseudo-simple SNP according to the allelic SNP types, availability of inter-homoeologue, and consequently the locus numbers a probe can bind to. The Simple SNP refers to a typical allelic SNP, which can be only targeted by its specific probe (a single locus). Such a detection generates AA/BB/NC but no AB signal in both the parental lines and their offspring DH lines. The Hemi-SNP refers to the incomplete allelic polymorphisms due to the presence of homoeologous sequences in the *B. napus* genome. The Pseudo-simple SNP refers to an allelic SNP derived from two homoeologues that possess inter-homoeologous polymorphisms in two parental lines. In a Hemi-SNP locus, the existence of mismatch bases would result in a difference in probe binding capacity [50-53]. For instance, if the P1 can bind two loci as shown in Figure 4 (right), P1_Locus1 is of the genotype A that has a 100% binding capacity to the SNP probe, whereas P1_Locus2 is of genotype B that would have a decreased binding capacity with as few as three mismatch sites in a 50 bp-long probe [50-53]. Such a binding difference would result in a heterozygous AB signal (Figure 4). On the other hand, if the probe failed to bind Locus2 due to the competition with Locus1, there would be an incorrect classification of AA (the genotypes of Locus1). To analyze the possibility of the occurrence of such an error, we set out to assess the stability and error of the SNP detection system by calculating the PNPG in SNP loci. In the two-dimensional matrix described above, the PNPG of the horizontal SNP loci (PNPG__SNP_) can be calculated with the following formula:

$$ PNP{G}_{\_SNP}=\frac{1-P{G}_i}{M_i} $$

Where PNPG__SNP_ represents the percentage of NPGs in the i^th^ SNP loci of all DH lines, PG_i_ represents the number of PGs in the i^th^ SNP loci of all DH lines, and M_i_ represents the number of detected DH lines in the i^th^ SNP loci. After excluding the unauthentic DH lines, if the genotypes of both parents are AA at a SNP locus, the genotype of all DH lines is theoretically AA at this SNP locus. If a different genotype (such as AB or BB) is detected, it is very likely to be the result of a detection error. In this case, a PNPG value of 0.05 was still considered as the threshold to determine the reliability of call for a SNP locus.

Next, the PNPGs for 3,456 monomorphic SNPs in the horizontal direction were analyzed. A PNPG ≥0.05 was found at 108 (3.13%) SNP loci, indicating that the detection of most of the SNP loci was reliable. When excluding these 108 SNP loci, the average PNPG for the remaining SNP loci was 1.60E-03 (Table 2). The analysis of the remaining 3,348 SNP loci showed that if the genotypes of both parents were homozygous (AA or BB), the ratio of detecting a NPG in the population was <0.005; if the genotypes of both parents were heterozygous (AB), the ratio of detecting a homozygous genotype in the population was <0.05; and if both parents were detected as “no call” (NC), the PNPG was even lower (Table 2). These results suggest that if the PNPG is >0.05 at a SNP locus of a *B. napus* SNP array, it is most likely caused by inter-homoeologue polymorphism or signal superposition of multiple SNP loci. The generation of these heterozygous signals (AB) is due to the complexity of the genome of allotetraploid *B. napus*.

**Table 2:** **The proportion of each theoretically possible genotype in the monomorphic SNP loci**

**Genotype**	**Probe**	**Percentage of parental and non-parental genotype** ^**a**^				**PNPG** ^**c**^
		**AA**	**AB**	**BB**	**NC**	
**AA**	**1317**	**0.998** ^**b**^	8.91E-05	9.76E-05	1.42E-03	1.60E-03
**AB**	**16**	3.49E-04	**0.988**	1.05E-03	1.01E-02	1.15E-02
**BB**	**1441**	1.51E-04	1.24E-04	**0.998**	1.83E-03	2.10E-03
**NC**	**574**	9.73E-06	9.73E-06	9.73E-06	**1.000**	2.92E-05
**Total**	**3348**					**1.60E-03**

^a^Percentages of parental and non-parental genotype are calculated as: number of each detected genotype/(179 analyzed DH lines × probe number for each genotype).

^b^Percentages of parental genotypes were bolted.

^c^Percentage of non-parental genotype.

The above analysis showed that, using a two-dimensional matrix constructed with the genotypes of the monomorphic SNPs in the DH population and PNPG analysis, unauthentic DH lines could be excluded (using columns in the matrix), and the error and stability of the system could be estimated (using rows). These analyses can improve the quality and utilization efficiency of SNP array data.

### Bi-filtering analysis can reduce the interference of mHemi-SNP and Pseudo-simple SNP loci

Previously, the assignment of polymorphic SNP loci was conducted using two methods. The first method is to simply remove the loci that exhibit heterozygous signals in one of the parents, and the other method is to mark signals in the segregating population that have the same P1 value as genotype A, signals that have the same value of P2 as B, and non-parental signal values with missing values (“-”) [26,27,30]. The premise of the second method is the specific binding of a SNP probe to a locus in the genome. Due to the existence of a large number of inter-homoeologues in the genome of *B. napus*, a considerable number of the SNP probes cannot meet this requirement. To reduce the impact of multi-loci SNPs on the subsequent genetic linkage analysis, we further divided Hemi-SNPs into two sub-groups, sHemi-SNPs and mHemi-SNPs, according to whether the NPG can be identified in the DH population. A so-called sHemi-SNP refers to the probe call that generates heterozygous signal (AB) in one of the two parental lines and the parental genotypes can be detected but no NPG will be produce detected in their offspring DH lines. As illustrated in Figure 4 (group 8–11), a sHemi-SNP may include two of three possible signals (genotypes, AA/BB/AB) in parental lines, and for each group of parental genotype, their offspring DH lines can only produce parental genotypes. Furthermore, such a segregation of two different parental genotypes fits into the expected frequency. In contrast, the mHemi-SNP produces an extra non-parental signal in the offspring DH lines in addition to parent-type signals (genotypes) due to more mismatched bases available in the inter-homoeologue (Figure 4, group 12–16), which may result in no hybridization signal and consequently a null detection for one of the inter-homoeologous sequence. Obviously, the genotypes of mHemi-SNPs and Pseudo-simple SNPs are a superposition of the signals from multiple SNP loci, which cannot represent the corresponding genotype of the probe itself (Figure 4). Therefore, the mHemi-SNPs and Pseudo-simple SNPs should be removed to avoid any impact on the calculation of the linkage between these loci.

To identify the difference between mHemi-SNP and Pseudo-simple SNP and the other two types of SNPs (Simple SNP and sHemi-SNP), a method similar to the one above used for the analysis of monomorphic SNPs was applied to analyze the PNPG values of polymorphic SNP loci.

If the genotype of the parents was AB/BB of the two types of SNP: sHemi-SNP and mHemi-SNP, the PNPG values in the DH population were different (Figure 4, group 10, 11, 14, and 15). Considering the two inter-homoeologous sequences YY and RR in P1 and their alleles yy and rr in P2 as two independent loci in the genome, the DH population will expect four genotypes of YYRR, YYrr, yyRR, and yyrr with the fixed frequency of 1/4 for each through the haploid production (Figure 4, top left). In Pseudo-simple SNP type (Figure 4, lower left), only a same set of signals as Simple SNP (Additional file 2: Figure S1a-S1b) are detected in the parental lines but there will be NPGs segregation in the DH population in the first five groups (Figure 4, group 1–5; Additional file 2: Figure S1c-S1f). In sHemi-SNP type, there will be AB (heterozygous) signal detected due to the presence of hemi-SNP but will no NPG in the DH population (Figure 4, top right; Additional file 2: Figure S1i-S1j). In the mHemi-SNP type, the signal values are similar to sHemi-SNP in parental lines but there will be NPG signals detected in the DH populations due to multiple mismatched nucleotides within the inter-homoeologous sequences (Figure 4, lower right; Additional file 2: Figure S1k-S1m). Furthermore, assuming that Locus1 and Locus2 have no linkage relationship, the expected frequencies of signal values (reflecting the corresponding genotypes) in the DH population could be deduced according to parental signals (Figure 4).

Once we have the expected frequencies for all possible four genotypes in the DH population, we can easily distinguish different types of SNPs listed in Figure 4. Since we can calculate the expected frequencies of PGs and NPGs in each group, we introduced a statistics of chi-squared test (χ^2^ test) as the probability of NPGs appearance (expected frequency versus observed frequency) in the DH population. We used PNPG =0.05 as the threshold to judge the presence of NPG or not.

There was an exception in the type of Pseudo-simple SNP for above analysis, where the two polymorphic genotypes of AA/NC and BB/NC (Figure 4, group 6 and 7; Additional file 2: Figure S1g-S1h) cannot be distinguished from a typical Simple SNP through the PNPG values. However, the frequencies of the two parental signal values in the DH population of the Simple SNPs of AA/NC and BB/NC were 0.5 and 0.5, respectively, while the frequencies of AA/NC and BB/NC in the Pseudo-simple SNPs (group 6 and 7) were 0.75 and 0.25, respectively (Figure 4, group 6 and 7; Additional file 2: Figure S1g-S1h). Therefore, the frequencies of these two polymorphic genotypes of AA/NC and BB/NC can be used to distinguish whether the SNP was Simple SNP (0.5/0.5) or Pseudo-simple SNPs (0.75/0.25). It was noted that two parents with AB signals also generated three clear clusters of AA, AB, and BB signals (Additional file 2: Figure S1n).

Based on the PNPG values of the SNP loci, the genotype data for several other polymorphic genotypes were identified from the single-locus sHemi-SNPs (PNPG <0.05). There were 175 (9.5%) SNP loci for the two polymorphic genotypes of AA/NC and BB/NC, including 53 loci with PNPG values >0.05; the remaining 122 loci (6.6% of the total polymorphic loci) can be separated into 80 Simple SNPs (*P* = 0.4391) and 42 Pseudo-simple SNPs (*P* = 0.0012) by χ^2^ test.

Based on the above analysis, the SNP loci with PNPG values >0.05 can be considered as multi-loci SNPs (mHemi-SNPs and Pseudo-simple SNPs, the genotypes of AA/NC and BB/NC could be distinguished by examination of their segregation ratios), whereas the SNP loci with PNPG values <0.05 were considered as single-locus SNPs (Simple SNPs and sHemi-SNPs). Based on this standard, 1,573 SNP loci (85.0%) were screened from 1,850 polymorphic SNP loci for the subsequent analysis. Using the PNPG value and χ^2^ test to extract single-locus SNPs (Simple SNPs and sHemi-SNPs) from the SNP array data can maximize the utilization of the total SNP loci and remove the multi-loci SNPs (mHemi-SNPs and Pseudo-simple SNPs), which were difficult to identify in previous studies.

It is worth to point out that homoeologous recombination between the A and C genomes might result in non-parental phenotype. There are two consequences if such recombination events happen. First, if a given SNP locus is located within the homoeologous recombination fragment, its genotype will be identified as a regular locus, no matter where the fragment is distributed in the *B. napus* genome. In this case, the locus cannot be assigned as a NPG count. Second, if such a SNP locus is located right at the breakpoint of the homoeologous recombination fragments, the locus will not be identified, thus resulting in a false NPG count. It is now known that the homoeologous recombination between the A and C genomes occurred in a relatively low frequency at the level of large fragments [2,30]. The probability that a given SNP locus is exactly located the breakpoint is rare. Therefore, it is reasonable to consider such a recombination event neglectable.

The signal values of the 1,573 valid SNP loci were converted to genotype values. Genotypes that were the same as that of parent P1 were recorded as “A”, genotypes that were the same as that of parent P2 were recorded as “B”, and non-parental genotypes were treated as missing (“-”). We named this method of filtering out unauthentic lines and mHemi-SNP loci in SNP array data using the PNPG values as bi-filtering analysis. In brief, the bi-filtering method could be summarized as follows. First, we used the monomorphic SNPs to calculate the PNPG value of a given DH line (the number of the SNPs with non-parental genotypes of a given DH line divided by the total number of the genotyping SNPs of a given DH line) to filter out the unauthentic lines (Additional file 3: Figure S2); Second, we used the polymorphic SNPs to calculate the PNPG value of a given SNP locus (the number of the DH lines with non-parental genotypes of a given SNP divided by the number of the genotyped DH lines of a given SNP) to filter the mHemi-SNPs and Pseudo-simple SNP (Additional file 3: Figure S2). The bi-filtering method not only makes use of the monomorphic SNPs to identify unauthentic lines and to assess possible errors in SNP array detection, but also uses the polymorphic SNPs more accurately. The method thus can improve the efficiency and accuracy of the SNP array data with a large portion of heterozygous signals, which is common in the analysis of high-throughput genotyping in polyploid species [16,54]. The bi-filtering method was also suitable for analyzing the genotyping data by re-sequencing of the population and parents. A flow diagram was constructed for analyzing of the high-throughput genotyping data (re-sequencing and SNP array) of the bi-parental populations (Additional file 3: Figure S2). However, more specific work will be needed to verify the effect of the bi-filtering method for analyzing re-sequencing data.

### The bi-filtering analysis improves the quality of genetic linkage map

**Figure 5:**
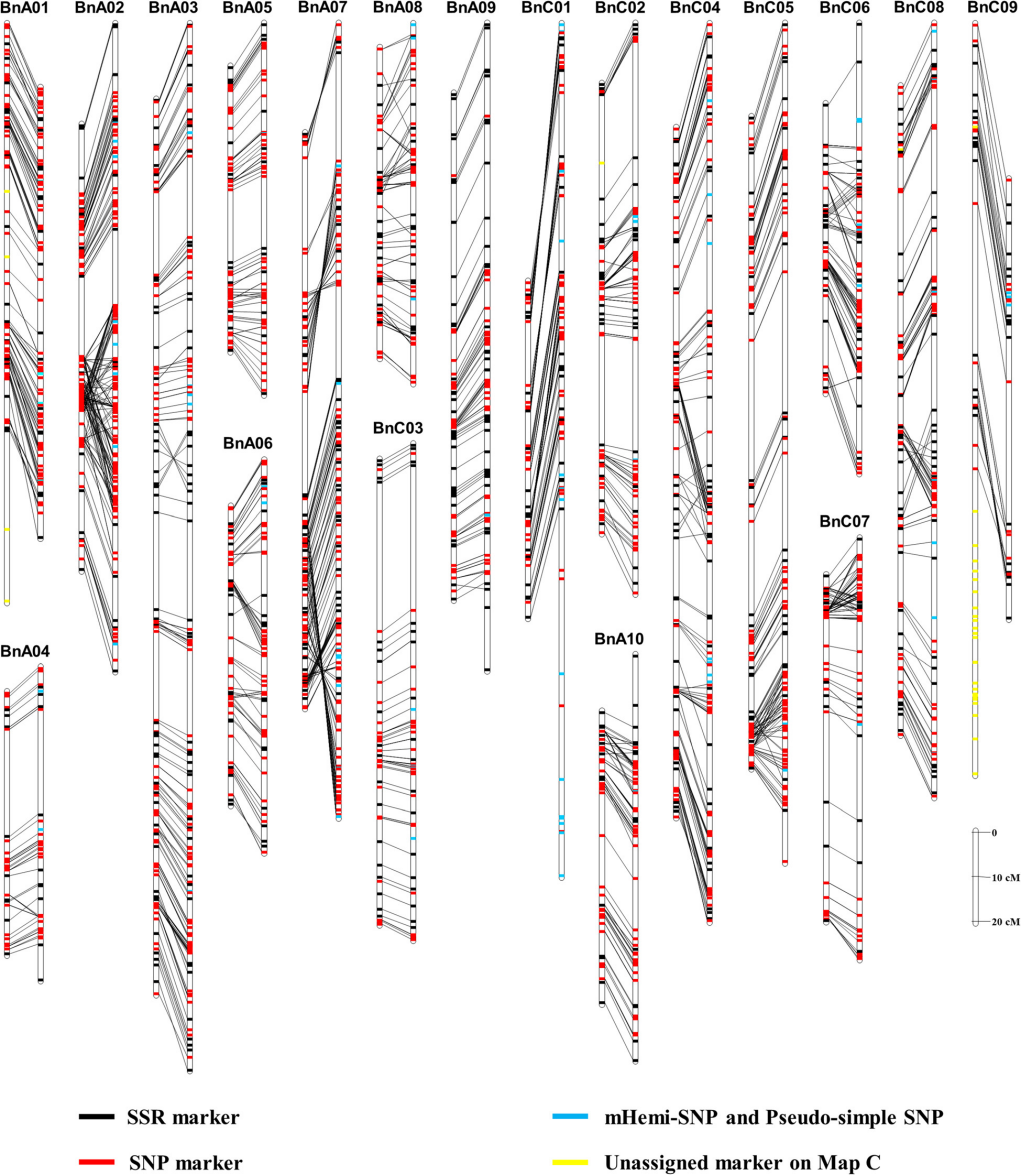
Comparison of the Map B and Map C with the HJ-DH population. The left and right vertical bar of each panel represents the linkage groups (LGs) of the Map B and Map C, respectively. Each LG and markers are represented with a vertical bar and transverse line, respectively. The same markers between the LG of the Map B and Map C are connected with black lines. The simple sequence repeat (SSR), single nucleotide polymorphism (SNP), mHemi-SNP and Pseudo-simple SNP, and the marker that can only be assigned on the Map B are shown with the black, red, blue and yellow transverse line, respectively. The data of the Map C comes from Cai *et al.* [30].

Previously, we constructed a genetic map (Map C) with 190 DH lines and 2,323 polymorphic markers (1,850 SNPs and 473 SSRs) [30] by means of the conventional method that uses simple substitution of genotypes based on the signal value of the parents [26,27]. Linkage analysis mapped 2,115 markers in 19 linkage groups (LGs) on the Map C, which was 2,477.4 cM in length with an average spacing of 1.27 cM between the markers [30]. To assess the effect of the SNP array data processed with bi-filtering analysis on the quality of genetic map, we constructed a new version of the genetic map with processed data (Figure 5, Additional file 4: Table S2, and Additional file 5: Table S3) and compared such a map with the Map C. After a bi-filtering analysis of both mapping population and SNP markers as described above, 179 DH lines and 2,046 polymorphic loci (1,573 SNPs and 473 SSRs) were used to produce a genetic map. Linkage analysis finally placed 2,014 loci onto 19 LGs (Figure 5, Additional file 4: Table S2, and Additional file 5: Table S3) and resulted in a new version of the genetic map (Map B) with total length of 2,020.3 cM and an average spacing of 1.00 cM (Additional file 4: Table S2 and Additional file 5: Table S3).

Compared with the Map C, the Map B now had an increased marker density after bi-filtering analysis of both unauthentic DH lines (11 lines) in mapping population, and mHemi-SNP and Pseudo-simple SNP markers in SNP arrays (Figure 5, Additional file 4: Table S2, and Additional file 5: Table S3). There were 208 markers (9.0% of the total polymorphic markers) that could not be located on the genetic map previously, while the ratio was reduced to 1.6% (32 markers) on the Map B (Figure 5, Additional file 4: Table S2, and Additional file 5: Table S3). Interestingly, all filtered 1,573 SNPs were all mapped on the Map B. There were 132 of the mHemi-SNP and Pseudo-simple SNP markers included in the Map C, which were excluded by the Map B (Figure 5, Additional file 4: Table S2, and Additional file 5: Table S3).

On the LGs with fewer mHemi-SNPs and Pseudo-simple SNPs, such as LGs A04, A05, A09, C03 and C05, the two maps showed good consistency (Figure 5, and Additional file 5: Table S3). However, other LGs exhibited obvious inconsistent, especially in the regions harboring mHemi-SNPs and Pseudo-simple SNPs, suggesting that mHemi-SNPs, Pseudo-simple SNPs, and unauthentic DH lines affected the mapping quality. First, the unauthentic DH lines may cause the exchange of the marker positions. For instance, several regions on LGs A06 (30.1-69.7 cM), A08 (29–61 cM), C07 (0–18.2 cM) and C08 (86–107.7 cM) or their neighboring regions contained few mHemi-SNPs and Pseudo-simple SNPs in the Map C (Figure 5, Additional file 6: Figure S3, Additional file 4: Table S2, and Additional file 5: Table S3), but there were obvious marker rearrangements and inversions in the regions (indicated by crossed lines in Figure 5 and Additional file 6: Figure S3). The positions and orders of the markers in these regions became consistent with the Map B after removal of the 11 unauthentic DH lines (Additional file 6: Figure S3). Second, the mHemi-SNPs and Pseudo-simple SNPs could result in lower marker density of the Map C. There were obvious marker rearrangements and inversions in the regions of LGs A03 (0–132.3 cM; 9 mHemi-SNPs and Pseudo-simple SNPs), A07 (0–155.9 cM; 16 mHemi-SNPs and Pseudo-simple SNPs), C01 (0–190.1 cM; 18 mHemi-SNPs and Pseudo-simple SNPs) of the Map C due to the existence of mHemi-SNPs and Pseudo-simple SNPs (Figure 5 and Additional file 7: Figure S4). However, the positions and orders of the markers in these regions became consistent between the two Maps when the mHemi-SNPs and Pseudo-simple SNPs in these regions were removed (with the 11 unauthentic DH lines retained; Figure 5 and Additional file 7: Figure S4). Third, mHemi-SNPs, Pseudo-simple SNPs, and the unauthentic DH lines may impose influences jointly. For instance, in the regions of LGs A02 (62.8-122.8 cM; 6 mHemi-SNPs and Pseudo-simple SNPs), C02 (41.2-62.2 cM; 5 mHemi-SNPs and Pseudo-simple SNPs), C04 (138.8-174.1 cM; 4 mHemi-SNPs and Pseudo-simple SNPs) and C06 (21.2-61.7 cM; 7 mHemi-SNPs and Pseudo-simple SNPs) of the Map C that contained more mHemi-SNPs and Pseudo-simple SNPs, two maps of these regions showed obvious inconformity (Figure 5, Additional file 4: Table S2, and Additional file 5: Table S3). It was found that mHemi-SNPs and Pseudo-simple SNPs could cause a pseudo genetic linkage relationship between mHemi-SNP and Pseudo-simple SNP and other markers. In total, 87 mHemi-SNPs and Pseudo-simple SNPs were located on LGs A02, A03, A07, C01, C04, C06 and C08 of Map C, respectively, which resulted in excess fragments or markers on the Map C (Figure 5, Additional file 4: Table S2, and Additional file 5: Table S3). Other mHemi-SNPs and Pseudo-simple SNPs were dispersed in the Map C and thus resulted in a decrease in the mapping density. Due to such interferences, there were 77 Simple SNPs that could not be mapped on the genetic map, while all of these Simple SNPs were linked to the genetic map after bi-filtering analysis (Figure 5, and Additional file 5: Table S3).

Since the linear relationship of the SSR marker loci on each of the LGs in both the Map B and C have been proved in different maps [45,46,55-58], a framework map of SSRs could thus serve as a reference to evaluate the linear relationships of SNP loci. To further compare the difference between the two maps, the graphical genotype of each DH line was constructed with the genotyping data from SSR markers and SNP markers processed with bi-filtering and conventional method, respectively. The graphical genotype of each DH line exhibited good collinearity between the framework map of SSRs and the Map B (Additional file 8: Figure S5). However, the graphical genotypes based on the Map C showed pseudo exchange fragments (caused by inversion, translocation and pseudo chromosome fragments) in some DH lines, especially in the LGs with more mHemi-SNPs and Pseudo-simple SNPs (Additional file 8: Figure S5).

Based on the above analysis, we concluded that a screening of mHemi-SNPs, Pseudo-simple SNPs, and the unauthentic DH lines for the construction of genetic maps was important. The bi-filtering analysis can remove mHemi-SNPs, Pseudo-simple SNPs, and unauthentic DH lines, thus improving the quality of a genetic map as observed in the Map B. With more loci included in future higher density SNP arrays, such as 60 K SNP arrays [26,28,29], more mHemi-SNPs and Pseudo-simple SNPs were expected to be filtered and the mapping quality would be further improved.

### The bi-filtering analysis increase the accuracy of QTL mapping

Next, we analyzed if the mHemi-SNP and Pseudo-simple SNP loci could have any adverse effects on QTL mapping. Results of QTL mapping of 20 agronomic traits in four environments were compared between the two maps. On the whole, 346 and 364 QTLs of 20 traits were identified by the Map B and Map C, respectively. There were 36 QTLs located on the excess fragments of LGs A03, A07, C01, C06 and C08 in the Map C, which can explain the contribution of phenotype (*R*^*2*^) 2.68-21.56% (on average 9.96%), with logarithm of odds (LOD) score 3.66-21.1 (on average 7.07). However, these QTLs could not be identified in the Map B, because the pseudo fragments have been filtered and the pseudo-QTLs resulted from the mHemi-SNPs and Pseudo-simple SNPs eliminated. Moreover, the mHemi-SNPs and Pseudo-simple SNPs dispersed along the different LGs of the Map C also affected the QTL identification. There were 18 QTLs that could be identified in the Map B but not in the Map C. Above data thus illustrated that the mapping accuracy of QTLs can be affected to a significant extent by mHemi-SNPs and Pseudo-simple SNPs.

**Figure 6:**
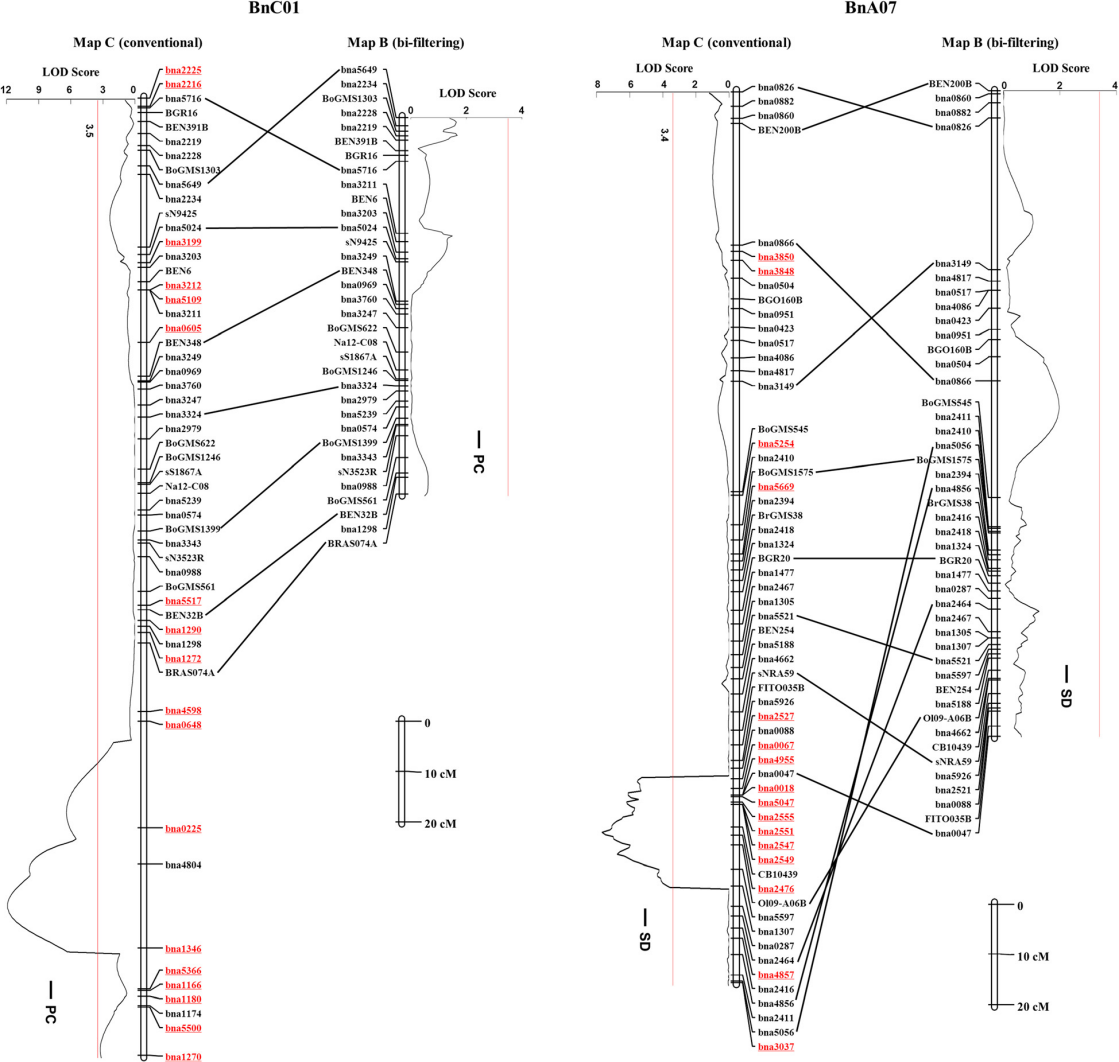
Comparison of genetic map construction and quantitative trait locus (QTL) mapping on the linkage groups (LGs) A07 and C01 of the HJ-DH population with the Map B and Map C. Only a sub-set of molecular markers are presented for each genetic LG. The single nucleotide polymorphism (SNP) markers with underlined (red color) are the mHemi-SNPs and Pseudo-simple SNPs. The detailed information about the LGs of the Map B is described in Additional file 3: Table S3, and the data of the Map C comes from Cai *et al.* [30]. The sub-set same molecular markers between the two LGs depicted are aligned with black lines. PC: protein content; SD: silique density. Significance thresholds for QTLs at the level P = 0.05 are estimated based on 1000 permutation.

In order to more clearly illustrate this effect, we focused on the QTLs on the LGs A07 and C01 (Figure 6). In Map C, each of these two LGs contained 18 and 16 mHemi-SNPs and Pseudo-simple SNPs respectively (Additional file 4: Table S2), which caused an inversion in the upper portion and an extra fragment with a length of approximately 82 cM in the lower portion of the LG C01. In this region, a major QTL of seed protein content (PC) with a LOD score of 11.9 and a contribution to the phenotype of up to 18.1% was detected. However, this QTL was no longer detectable in LG C01 in the Map B, in which these mHemi-SNPs and Pseudo-simple SNPs were eliminated. Such a difference suggested that these mHemi-SNPs and Pseudo-simple SNPs could cause the erroneous detection of QTLs. Similarly, for the LG A07, the presence of 16 mHemi-SNPs and Pseudo-simple SNPs led to disorder in the linkage relationship for the markers on A07 (Figure 6). Similarly, there were 6 pseudo QTLs identified in this region on Map C. Of which a QTL for silique density (SD) with a LOD score of 7.66 and a phenotypic contribution of up to 11.7% was mapped, but no QTL was detectable in the Map B. Taken together, these results indicate that the mHemi-SNPs and Pseudo-simple SNPs could interfere with the establishment of the linkage relationship between the markers and subsequently affect the subsequent QTL mapping, as well as candidate gene analysis, although they only accounted for 6.1% of the total number of markers in the Map C. Therefore, the removal of the unauthentic lines, mHemi-SNPs, and Pseudo-simple SNPs from the population using the bi-filtering method could improve the accuracy of a genetic map that is crucial for subsequent analyses.

## Conclusions

We have developed a novel bi-filtering method to effectively identify unauthentic DH lines as well as mHemi-SNP and Pseudo-simple SNP loci resulted from the superposition of the multiple SNP loci signals in SNP arrays. Such a bi-filtering analysis of the SNP array data can maximize the use of the SNP array data more accurately in polyploid species, to which many important crops belong. The power of the method would be more obvious in higher density arrays where manual filtering analysis will become difficult.

## Additional files

Additional file 1: Table S1. Detailed information of PNPG of the DH population, monomorphic SNP loci and the two-dimensional matrix of the monomorphic SNP loci genotypes in the HJ-DH population. Additional file 2: Figure S1. Clusters of different types SNPs in the DH population without 11 unauthentic DH lines. The SNP classification and group number for each cluster are the same to that of Figure 4. Additional file 3: Figure S2. A flow diagram for analysis the high-throughput genotyping data (re-sequencing and SNP array) of the bi-parental populations. Additional file 4: Table S2. Parameters of two genetic maps constructed by the bi-filtering analysis method (Map B) and the conventional method (Map C). The data of the Map C comes from Cai *et al.* [30]. Additional file 5: Table S3. Detailed information of the genetic linkage maps of the HJ-DH population constructed by the bi-filtering analysis method (Map B), the homoeologous loci and homoeologous collinear fragments identified in *B. rapa* and *B. oleracea*. Additional file 6: Figure S3. Effects of unauthentic DH lines on the localization of the SNP markers on linkage groups (LGs) A06, A08, C07, and C08. The left, middle and right vertical bars of each panel represents the LGs constructed with the data from the Map C without the 11 unauthentic DH lines, the Map B, and the Map C with 11 unauthentic DH lines, respectively. Each LG and markers are represented with a vertical bar and transverse line, respectively. The same markers between the LG of these three maps are connected with black lines. The simple sequence repeat (SSR), single nucleotide polymorphism (SNP), mHemi-SNP and Pseudo-simple SNP, and the marker that can only be assigned on the Map B are shown with the black, red, blue and yellow transverse line, respectively. The data of the Map C were adopted from Cai *et al.* [30]. Additional file 7: Figure S4. Effects of mHemi-SNPs and Pseudo-simple SNPs on the localization of the SNP markers on linkage groups (LGs) A03, A07, and C01. The left, middle and right vertical bars of each panel represents the LGs that are constructed with the data from the Map C without the mHemi-SNPs and Pseudo-simple SNPs in 190 DH lines, the Map B, and the Map C with the mHemi-SNPs and Pseudo-simple SNPs in 190 DH lines, respectively. Each LG and markers are represented with a vertical bar and transverse line, respectively. The same markers between the LG of these three maps are connected with black lines. The simple sequence repeat (SSR), single nucleotide polymorphism (SNP), mHemi-SNP and Pseudo-simple SNP, and the marker that can only be assigned on the Map B are shown with the black, red, blue and yellow transverse line, respectively. The data of the Map C were adopted from Cai *et al.* [30]. Additional file 8: Figure S5. The graphical genotypes of three DH lines (DH2, DH3 and DH81) on LGs A07, C01 and C04 constructed by the SSR, bi-filtering and conventional method, respectively. In each panel, the left, middle and right LG constructed by the SSR, bi-filtering and conventional method, respectively. The data of the Map C comes from Cai *et al.* [30]. In each LG, the horizontal bars represent molecular markers, the red, blue and black color represents P1, P2 and missing genotype, respectively. Arrows indicates the pseudo fragments in the genetic map constructed by the conventional method, which did not exist in the maps constructed by the SSR and bi-filtering methods.

## Abbreviations

CSS: Cluster separation score
MAF: Minor allele frequency
PG: Parental genotype
NPG: Non-parental genotype
PNPG: Percentage of non-parental genotype
sHemi-SNP: Hemi-SNP from a single locus
mHemi-SNP: Hemi-SNP from multi-loci
PC: Protein content
SD: Silique density
