# Supplementary figures and images for: A bi-filtering method for processing single nucleotide polymorphism array data improves the quality of genetic map and accuracy of quantitative trait locus mapping in doubled haploid populations of polyploid Brassica napus
Source: BMC Genomics. 2015 May 28;16(1):409. doi: 10.1186/s12864-015-1559-4 (PMC4445301; doi:10.1186/s12864-015-1559-4)

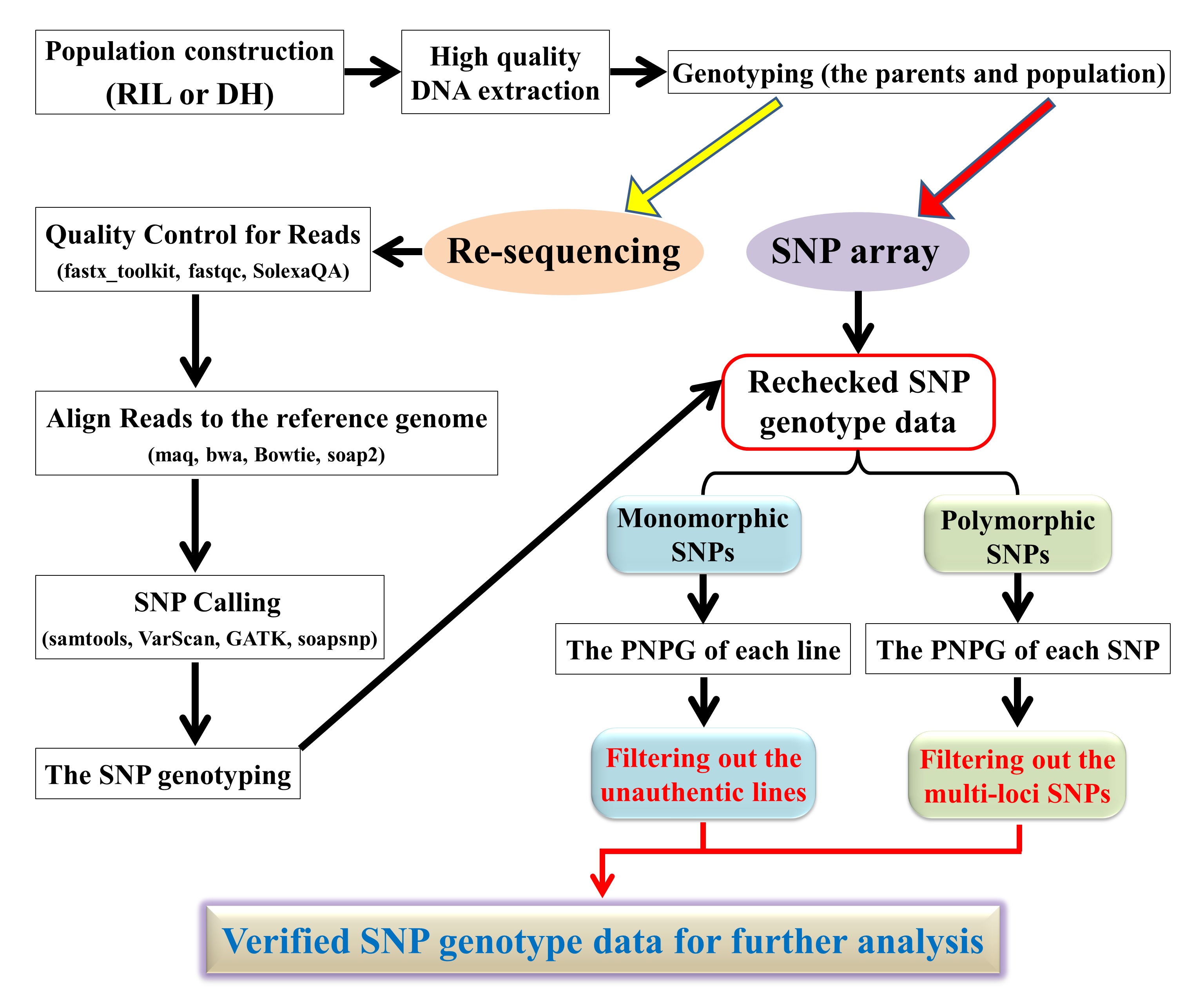

Supplement: Additional file 3: Figure S2. — A flow diagram for analysis the high-throughput genotyping data (re-sequencing and SNP array) of the bi-parental populations. [file 12864_2015_1559_MOESM3_ESM.tiff]

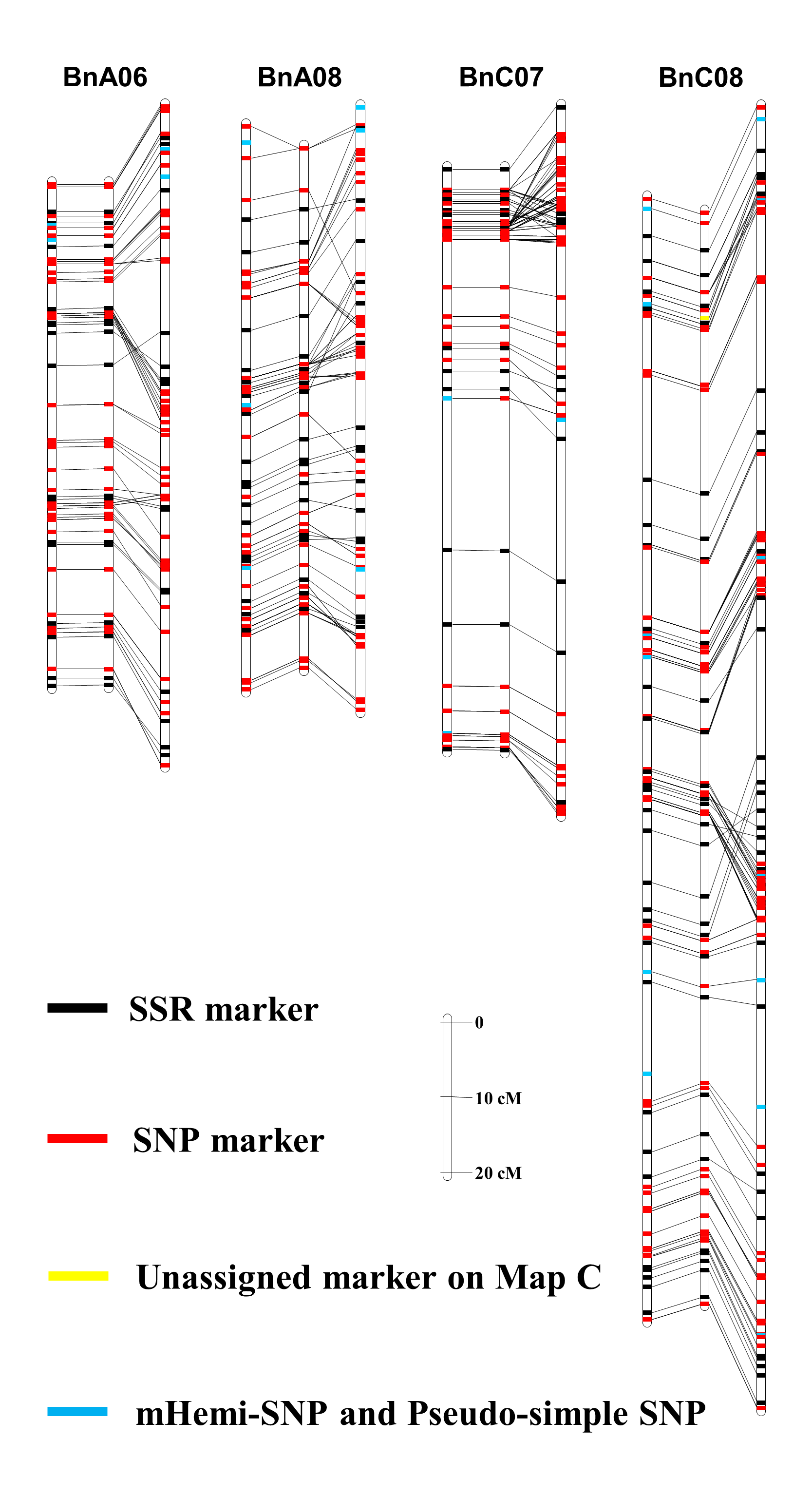

Supplement: Additional file 6: Figure S3. — Effects of unauthentic DH lines on the localization of the SNP markers on linkage groups (LGs) A06, A08, C07, and C08. The left, middle and right vertical bars of each panel represents the LGs constructed with the data from the Map C without the 11 unauthentic DH lines, the Map B, and the Map C with 11 unauthentic DH lines, respectively. Each LG and markers are represented with a vertical bar and transverse line, respectively. The same markers between the LG of these three maps are connected with black lines. The simple sequence repeat (SSR), single nucleotide polymorphism (SNP), mHemi-SNP and Pseudo-simple SNP, and the marker that can only be assigned on the Map B are shown with the black, red, blue and yellow transverse line, respectively. The data of the Map C were adopted from Cai et al. [30]. [file 12864_2015_1559_MOESM6_ESM.tiff]

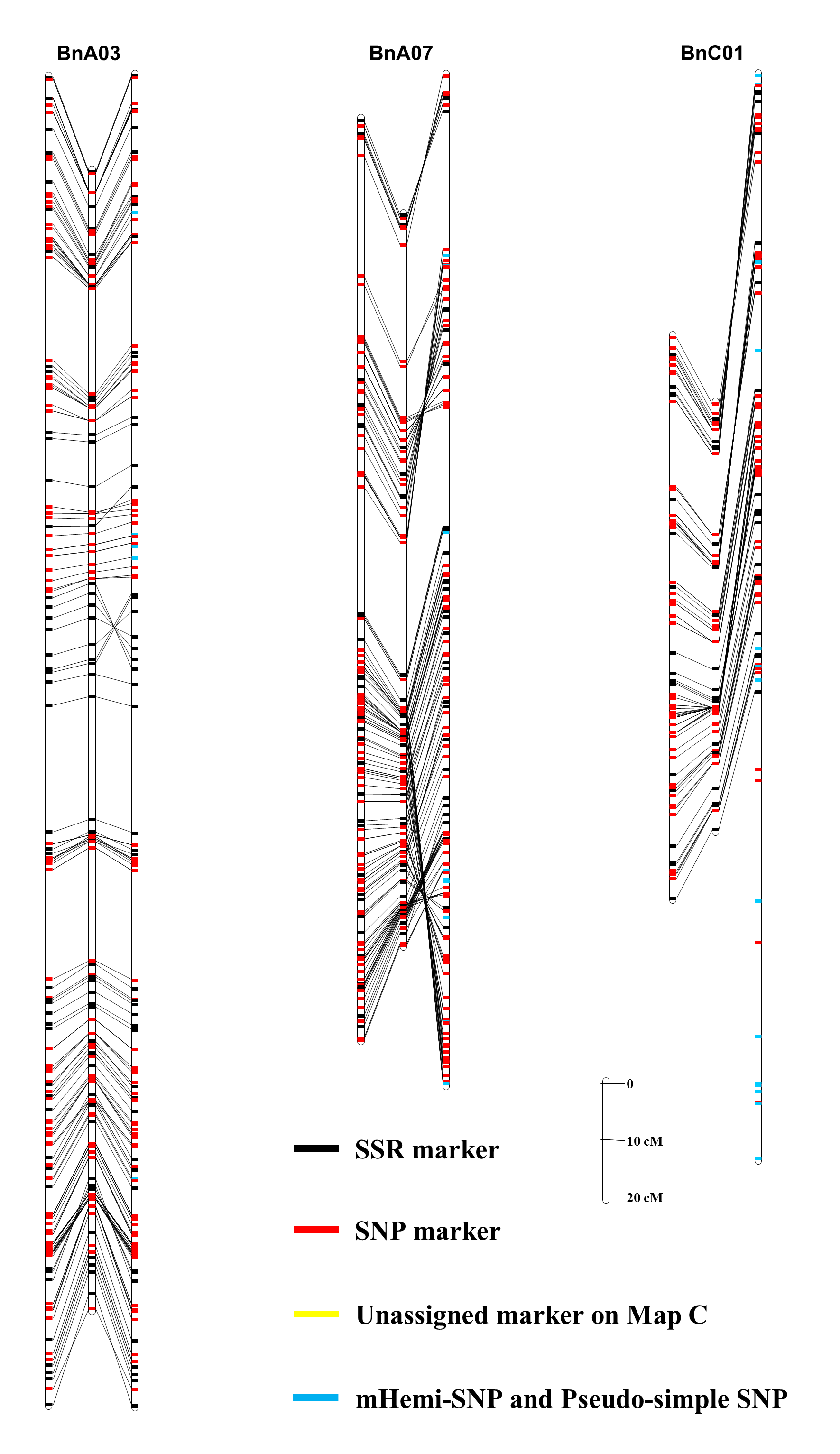

Supplement: Additional file 7: Figure S4. — Effects of mHemi-SNPs and Pseudo-simple SNPs on the localization of the SNP markers on linkage groups (LGs) A03, A07, and C01. The left, middle and right vertical bars of each panel represents the LGs that are constructed with the data from the Map C without the mHemi-SNPs and Pseudo-simple SNPs in 190 DH lines, the Map B, and the Map C with the mHemi-SNPs and Pseudo-simple SNPs in 190 DH lines, respectively. Each LG and markers are represented with a vertical bar and transverse line, respectively. The same markers between the LG of these three maps are connected with black lines. The simple sequence repeat (SSR), single nucleotide polymorphism (SNP), mHemi-SNP and Pseudo-simple SNP, and the marker that can only be assigned on the Map B are shown with the black, red, blue and yellow transverse line, respectively. The data of the Map C were adopted from Cai et al. [30]. [file 12864_2015_1559_MOESM7_ESM.tiff]

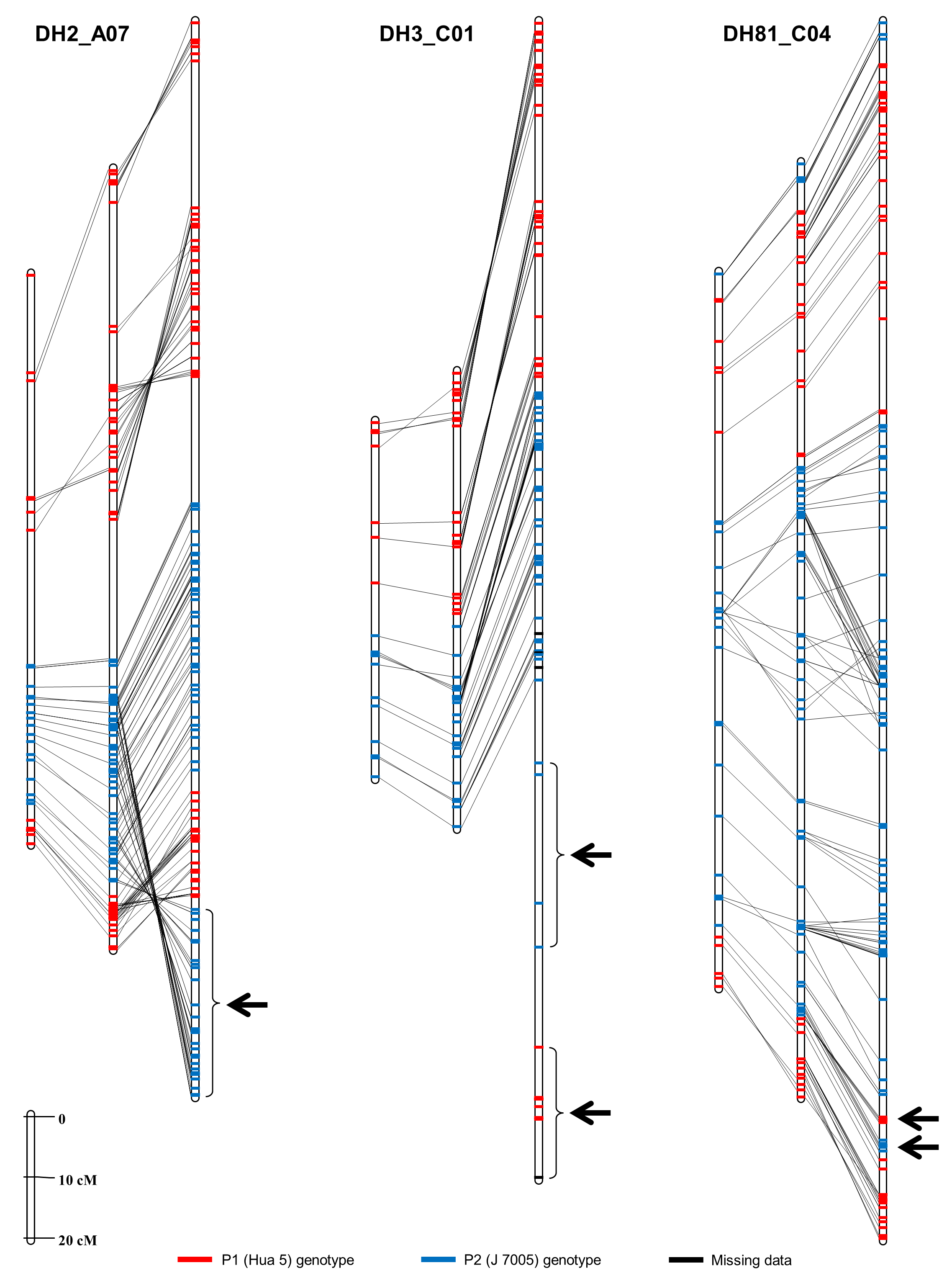

Supplement: Additional file 8: Figure S5. — The graphical genotypes of three DH lines (DH2, DH3 and DH81) on LGs A07, C01 and C04 constructed by the SSR, bi-filtering and conventional method, respectively. In each panel, the left, middle and right LG constructed by the SSR, bi-filtering and conventional method, respectively. The data of the Map C comes from Cai et al. [30]. In each LG, the horizontal bars represent molecular markers, the red, blue and black color represents P1, P2 and missing genotype, respectively. Arrows indicates the pseudo fragments in the genetic map constructed by the conventional method, which did not exist in the maps constructed by the SSR and bi-filtering methods. [file 12864_2015_1559_MOESM8_ESM.tiff]
