# Supplementary material for: A bi-filtering method for processing single nucleotide polymorphism array data improves the quality of genetic map and accuracy of quantitative trait locus mapping in doubled haploid populations of polyploid Brassica napus
Source: BMC Genomics. 2015 May 28;16(1):409. doi: 10.1186/s12864-015-1559-4 (PMC4445301; doi:10.1186/s12864-015-1559-4)
Supplement: Additional file 4: Table S2. — Parameters of two genetic maps constructed by the bi-filtering analysis method (Map B) and the conventional method (Map C). The data of the Map C comes from Cai et al. [30]. [file 12864_2015_1559_MOESM4_ESM.pdf]

Table S2 Parameters of two genetic maps constructed by the bi-filtering method (Map B) and the conventional method (Map C) in the HJ-DH population of *B. napus*

LG	Map B					Map C [a]					
	**Total marker**	**SSR marker**	**SNP marker**	**Length (cM)**	**Average distance (cM/marker)**	**Total marker**	**SSR marker**	**SNP marker**	**mHemi-SNP and Pseudo-simple SNP**	**Length (cM)**	**Average distance (cM/marker)**
**A1**	128	19	109	128.8	1.01	129	19	110	6	105.1	0.81
**A2**	177	15	162	99.2	0.56	190	15	175	13	144.8	0.76
**A3**	185	47	138	199.6	1.08	196	47	149	12	233.2	1.19
**A4**	47	10	37	58.3	1.24	51	11	40	3	69.5	1.36
**A5**	73	16	57	63.2	0.87	74	16	58	1	83.5	1.13
**A6**	74	16	58	66.4	0.90	78	16	62	4	87.4	1.12
**A7**	171	33	138	128.1	0.75	187	33	154	16	155.9	0.83
**A8**	77	17	60	68.9	0.89	81	17	64	4	79.9	0.99
**A9**	94	34	60	112.7	1.20	97	36	61	1	143.9	1.48
**A10**	88	18	70	64.9	0.74	93	20	73	4	90.3	0.97
**Subtotal**	**1114**	**225**	**889**	**990.1**	**0.89**	**1176**	**230**	**946**	**64**	**1193.5**	**1.07**
**C1**	94	18	76	74.8	0.80	117	18	99	18	190.1	1.62
**C2**	96	21	75	100.1	1.04	102	21	81	7	127.1	1.25
**C3**	55	22	33	103.5	1.88	59	22	37	4	110.4	1.87
**C4**	132	40	92	153.6	1.16	142	40	102	10	200.2	1.41
**C5**	136	27	109	145.2	1.07	140	27	113	4	186.9	1.34
**C6**	91	26	65	64.1	0.70	99	27	72	7	100.7	1.02
**C7**	108	15	93	77	0.71	112	15	97	4	93.7	0.84
**C8**	121	32	89	144.6	1.20	131	32	99	11	172.3	1.32
**C9**	67	15	52	167.3	2.50	37	16	21	3	102.5	2.77
**Subtotal**	**900**	**216**	**684**	**1030.2**	**1.14**	**939**	**218**	**721**	**65**	**1283.9**	**1.49**
**Total**	**2014**	**441**	**1573**	**2020.3**	**1.00**	**2115**	**448**	**1667**	**132**	**2477.4**	**1.27**

[a] The data of Map C is come from Cai *et al.* [30].
