# Supplementary material for: A bi-filtering method for processing single nucleotide polymorphism array data improves the quality of genetic map and accuracy of quantitative trait locus mapping in doubled haploid populations of polyploid Brassica napus
Source: BMC Genomics. 2015 May 28;16(1):409. doi: 10.1186/s12864-015-1559-4 (PMC4445301; doi:10.1186/s12864-015-1559-4)
Supplement: Additional file 5: Table S3. — Detailed information of the genetic linkage maps of the HJ-DH population constructed by the bi-filtering analysis method (Map B), the homoeologous loci and homoeologous collinear fragments identified in B. rapa and B. oleracea. [file 12864_2015_1559_MOESM5_ESM.pdf]

Table S3 Detailed information of the genetic linkage map of the HJ-DH population constructed by the bi-filtering analysis method (Map B), comparative analysis with *B. rapa* and *B. oleracea*

B. napus			***B. rapa*/*B. oleracea***		
**LG**	**Marker**	**Position**	**Homologous locus**	**Homologous collinear locus** [a]	
BnA01	bna1059	0	BrA01_284970_301 [b]	BrA01 [c]	284970
BnA01	bna1060	0	BrA01_316954_302	BrA01	316954
BnA01	bna5902	0.6	BrA01_344735_301	BrA01	344735
BnA01	bna5487	2.6	BrA01_405988_295	BrA01	405988
BnA01	BGO001	4.3	BrA01_759441_243	BrA01	759441
BnA01	BEN334	4.3	BoC01_983676_247		
BnA01	bna4875	5.7	BrA01_806580_171	BrA01	806580
BnA01	bna5488	5.7	BrA01_855235_301	BrA01	855235
BnA01	bna1065	6.5	BrA01_922051_301	BrA01	922051
BnA01	bna5005	6.5	BrA01_974796_295	BrA01	974796
BnA01	bna1067	6.5	BrA01_975490_301	BrA01	975490
BnA01	BEN9	7.6	BrA01_1040762_198	BrA01	1040762
BnA01	bna1070	9.1	BrA01_1103533_255	BrA01	1103533
BnA01	bna1073	11.1	BrA01_1291584_301	BrA01	1291584
BnA01	bna1074	11.7	BrA01_1322683_301	BrA01	1322683
BnA01	bna5432	11.7	BoC01_1878304_252		
BnA01	bna1076	12.3	BrA01_1439859_301	BrA01	1439859
BnA01	bna1093	12.3	BrA01_1459501_301	BrA01	1459501
BnA01	NIAB_SSR106	12.9	BrA01_1466151_286	BrA01	1466151
BnA01	bna5906	15.8	BoC01_2488980_301		
BnA01	bna1105	17.8	BrA01_1977120_301	BrA01	1977120
BnA01	bna1106	17.8	BrA01_2067347_301	BrA01	2067347
BnA01	bna1109	18.4	BrA01_2284682_297	BrA01	2284682
BnA01	bna1112	18.7	BrA01_2359318_213	BrA01	2359318
BnA01	BEN10	19	BrA01_2500870_113	BrA01	2500870
BnA01	BGO108	19.6	BrA01_2567859_160	BrA01	2567859
BnA01	bna1115	20.7	BrA01_2667541_301	BrA01	2667541
BnA01	bna1116	20.7	BrA01_2694173_301	BrA01	2694173
BnA01	bna1022	20.7	BoC04_18829093_301		
BnA01	bna1117	20.7	BrA01_2862804_302	BrA01	2862804
BnA01	Ol10-D03A	21.6	BrA01_3282738_133	BrA01	3282738
BnA01	bna1121	21.9	BrA01_3304097_297	BrA01	3304097
BnA01	bna5499	21.9	BrA09_35709086_301		
BnA01	bna0123	22.2	BrA01_3744574_303	BrA01	3744574
BnA01	bna5325	22.2	BoC01_4854933_314		
BnA01	bna1125	22.5	BrA01_3969016_301	BrA01	3969016
BnA01	bna4825	22.5	BrA01_4027253_301	BrA01	4027253
BnA01	bna3175	22.5	BoC01_5700363_302		
BnA01	bna3176	22.5	BoC01_5910378_284		
BnA01	bna5033	22.5	BrA01_4077993_301	BrA01	4077993
BnA01	bna1129	22.5	BrA01_4246436_301	BrA01	4246436

BnA01	BEN2	22.8	BrA01_4276372_187	BrA01	4276372
BnA01	BnEMS1012	22.8	BrA01_4276389_211	BrA01	4276389
BnA01	bna1131	23.7	BrA01_4612731_282	BrA01	4612731
BnA01	bna5497	24.8	BrA01_5022756_301	BrA01	5022756
BnA01	bna1142	28.4	BrA01_5971798_301	BrA01	5971798
BnA01	bna1145	28.7	BrA01_6379546_301	BrA01	6379546
BnA01	bna1146	28.7	BrA01_6388281_309	BrA01	6388281
BnA01	bna1147	28.7	BrA01_6502314_301	BrA01	6502314
BnA01	bna5365	29	BrA01_6681885_301	BrA01	6681885
BnA01	BGR65	29.3	BrA01_6684759_104	BrA01	6684759
BnA01	bna1150	29.6	BrA01_6898141_301	BrA01	6898141
BnA01	bna4633	31.6	BrA01_7399677_301	BrA01	7399677
BnA01	bna1154	31.6	BrA01_7473115_301	BrA01	7473115
BnA01	bna1155	31.6	BrA01_7486801_301	BrA01	7486801
BnA01	bna1156	31.6	BrA01_7530818_301	BrA01	7530818
BnA01	bna5269	31.6	BrA01_7554524_301	BrA01	7554524
BnA01	bna1157	31.6	BrA01_7721347_301	BrA01	7721347
BnA01	bna1159	31.9	BrA01_7855239_303	BrA01	7855239
BnA01	bna1160	31.9	BrA01_7991655_302	BrA01	7991655
BnA01	bna0317	37.2	BrA01_8388907_301	BrA01	8388907
BnA01	bna0318	37.2	BrA01_8395801_301	BrA01	8395801
BnA01	bna1168	41.8	BrA01_8514979_301	BrA01	8514979
BnA01	BnEMS57	45	BrA01_8639083_251	BrA01	8639083
BnA01	bna1181	46.5	BrA01_9203246_301	BrA01	9203246
BnA01	bna1174	51.8	BrA01_10000380_303	BrA01	10000380
BnA01	bna1880	55.1	BrA01_11302933_301	BrA01	11302933
BnA01	bna1202	57.8	BrA01_14804405_301	BrA01	14804405
BnA01	bna5331	57.8	BrA01_14915561_301	BrA01	14915561
BnA01	Na12-C06	62.4			
BnA01	BGR6	66.3	BrA01_14939760_138	BrA01	14939760
BnA01	bna1205	66.9	BrA01_14960083_301	BrA01	14960083
BnA01	bna4847	67.5	BrA01_16129488_301	BrA01	16129488
BnA01	bna1952	67.5	BrA01_16253613_301	BrA01	16253613
BnA01	bna1951	67.5	BrA01_16260435_301	BrA01	16260435
BnA01	bna1949	67.5	BrA01_16433096_275	BrA01	16433096
BnA01	bna1948	67.5	BrA01_16472423_301	BrA01	16472423
BnA01	bna1947	67.5	BrScaffold004113_153_300		
BnA01	bna5381	67.5	BrA01_16945994_301	BrA01	16945994
BnA01	bna5604	67.5	BrA01_17112397_301	BrA01	17112397
BnA01	bna1938	67.5	BrA01_17296297_299	BrA01	17296297
BnA01	bna1216	69.2	BrA01_18097124_301	BrA01	18097124
BnA01	bna5508	69.2	BoC01_25604906_301		
BnA01	bna1215	69.2	BrA01_18102853_269	BrA01	18102853
BnA01	BGR84	70.6	BrA01_19723537_119	BrA01	19723537
BnA01	bna4621	70.9			
BnA01	bna1234	71.2	BrA01_20436893_250	BrA01	20436893
BnA01	bna1240	72.3	BrA01_21533280_301	BrA01	21533280

BnA01	bna1227	72.9	BrA01_21715312_301	BrA01	21715312
BnA01	bna1226	72.9	BrA01_21726916_301	BrA01	21726916
BnA01	bna5240	73.8	BrA01_22141137_301	BrA01	22141137
BnA01	bna1242	73.8	BrA01_22680380_255	BrA01	22680380
BnA01	bna2684	74.1	BrA01_23024808_206	BrA01	23024808
BnA01	bna5050	74.1	BrA01_23024808_206	BrA01	23024808
BnA01	bna1244	74.1	BrA01_23159955_209	BrA01	23159955
BnA01	bna5184	74.1	BrA01_23169456_302	BrA01	23169456
BnA01	bna1246	74.1	BrA01_23390600_301	BrA01	23390600
BnA01	bna1250	74.1	BrA01_23571784_301	BrA01	23571784
BnA01	BEN68	74.7	BrA01_23659029_106	BrA01	23659029
BnA01	bna3349	75.3	BoC01_32601699_301		
BnA01	bna5513	75.3	BrA01_23718576_301	BrA01	23718576
BnA01	BnGMS87	75.9	BrA01_23784674_221	BrA01	23784674
BnA01	bna3358	76.2	BoC01_33124503_303		
BnA01	bna1260	76.5	BrA01_24487702_301	BrA01	24487702
BnA01	bna5255	76.5	BrA03_2569659_301		
BnA01	bna4831	77.9	BrA01_24694541_172	BrA01	24694541
BnA01	bna1269	77.9	BoC01_35362787_314		
BnA01	bna0776	77.9	BoC01_35286677_301		
BnA01	bna1277	78.5	BrA01_26384627_301	BrA01	26384627
BnA01	bna3367	78.5	BoC01_36821459_254		
BnA01	bna0344	78.5	BrA01_26442983_301	BrA01	26442983
BnA01	bna1280	78.5	BrA01_26605007_302	BrA01	26605007
BnA01	bna1281	78.5	BrA01_26622397_301	BrA01	26622397
BnA01	bna1293	78.5	BrA01_27159148_301	BrA01	27159148
BnA01	bna1295	78.5	BrA01_27389556_298	BrA01	27389556
BnA01	bna5656	78.5	BrScaffold000164_241494_301		
BnA01	bna1287	79.1	BrA01_27855099_301	BrA01	27855099
BnA01	BEN32A	83	BrA01_27859602_165	BrA01	27859602
BnA01	bna0962	88.3	BoScaffold000425_184083_301		
BnA01	bna0893	88.3	BoScaffold000425_84667_264		
BnA01	bna0963	88.3	BoScaffold000425_184083_301		
BnA01	bna2273	88.9	BrA01_28153164_301	BrA01	28153164
BnA01	bna0300	88.9	BrScaffold000191_156554_301		
BnA01	bna2272	88.9	BrA01_28168240_301	BrA01	28168240
BnA01	Na12-H02	90	BrA01_28229113_223	BrA01	28229113
BnA01	BRAS074B	90.6	BrA01_28424481_120	BrA01	28424481
BnA01	bna5274	112.7	BrA02_3965136_301		
BnA01	bna4804	128.8	BrA01_4737322_301	BrA01	4737322
BnA02	BoGMS795	0			
BnA02	Ol11-H09A	0.6	BrA02_1510618_161	BrA02	1510618
BnA02	sR12095	0.6	BrA02_1598382_324	BrA02	1598382
BnA02	BEN406	11.7	BoC02_5333538_128		
BnA02	bna4819	15.3	BoScaffold000328_209205_301		
BnA02	bna5528	15.6	BrA02_4103904_301	BrA02	4103904
BnA02	bna1349	15.6	BrA02_4120287_301	BrA02	4120287

BnA02	bna3673	15.6	BoC08_4067834_301		
BnA02	bna1351	15.6	BrA02_4180732_301	BrA02	4180732
BnA02	bna1971	16.8	BrA02_6252346_301	BrA02	6252346
BnA02	bna1367	17.4	BrA02_6466142_299	BrA02	6466142
BnA02	sN3761B	18.6	BrA02_6754912_171	BrA02	6754912
BnA02	bna1371	18.9	BrA02_6766467_301	BrA02	6766467
BnA02	bna4833	19.5	BrA02_7105284_302	BrA02	7105284
BnA02	sR6293	19.8	BrA02_7173113_124	BrA02	7173113
BnA02	bna5910	20.4	BrA02_7275448_301	BrA02	7275448
BnA02	bna3412	21.5	BrA02_7637663_301	BrA02	7637663
BnA02	bna5072	21.6	BrA02_7750536_301	BrA02	7750536
BnA02	bna1379	21.7	BrA02_7756158_301	BrA02	7756158
BnA02	bna1382	21.7	BrA02_7878079_301	BrA02	7878079
BnA02	bna5119	21.7	BrA02_7881877_301	BrA02	7881877
BnA02	bna1387	22	BrA02_8179342_301	BrA02	8179342
BnA02	bna5538	22.5	BrA02_8447213_285	BrA02	8447213
BnA02	bna1393	22.5	BrA02_8625702_301	BrA02	8625702
BnA02	bna1390	22.5	BoC06_25238316_301		
BnA02	bna0125	22.5	BrA02_8738403_301	BrA02	8738403
BnA02	bna1391	22.5	BrScaffold000318_1244_301		
BnA02	bna5442	22.8	BrA02_8884562_301	BrA02	8884562
BnA02	bna1398	22.8	BrA02_8949807_301	BrA02	8949807
BnA02	bna1401	22.8	BrA02_9281581_303	BrA02	9281581
BnA02	bna3513	23.1	BoScaffold000001_P2_469158_301		
BnA02	bna1698	24.3	BrA02_9421963_300	BrA02	9421963
BnA02	bna4638	24.3	BrA02_9645918_301	BrA02	9645918
BnA02	bna1441	26.5	BrA02_10325878_301	BrA02	10325878
BnA02	BEN281B	27.4	BrA02_10444068_202	BrA02	10444068
BnA02	bna2864	28	BoC01_19115033_293		
BnA02	bna0362	28.3	BrA02_10956748_301	BrA02	10956748
BnA02	bna1447	28.6	BrA02_11015035_301	BrA02	11015035
BnA02	bna1445	29.2	BrA02_11183543_301	BrA02	11183543
BnA02	bna1404	29.5	BrA02_11454601_301	BrA02	11454601
BnA02	bna1406	29.5	BrA02_11666536_303	BrA02	11666536
BnA02	bna1407	29.5	BrA02_11750590_301	BrA02	11750590
BnA02	bna5073	30.9	BrA02_12314392_301	BrA02	12314392
BnA02	bna5852	30.9	BrA02_12589154_301	BrA02	12589154
BnA02	bna1418	31.5	BrA02_12688756_301	BrA02	12688756
BnA02	bna1420	31.5	BrA02_12756742_296	BrA02	12756742
BnA02	bna1424	33.1	BrA02_13556242_301	BrA02	13556242
BnA02	bna0786	33.1	BoC02_18798720_286		
BnA02	BGR87	33.7	BrA02_14212023_120	BrA02	14212023
BnA02	bna3297	51.7	BoC07_36458580_302	BoC07	36458580
BnA02	bna5839	51.8	BoC07_36544565_301	BoC07	36544565
BnA02	bna3294	51.9	BoC07_36545063_301	BoC07	36545063
BnA02	bna3292	52	BoC07_36597360_301	BoC07	36597360
BnA02	bna5838	52.1	BoC07_36658059_301	BoC07	36658059

BnA02	bna3291	52.2	BoC07_36664505_301	BoC07	36664505
BnA02	bna3290	52.3	BoC07_36672987_301	BoC07	36672987
BnA02	bna3289	52.4	BoC07_36714679_301	BoC07	36714679
BnA02	bna5237	52.5	BoC07_36755053_277	BoC07	36755053
BnA02	bna3287	52.5	BoC07_36847163_301	BoC07	36847163
BnA02	bna4881	52.5	BoC07_36869769_222	BoC07	36869769
BnA02	bna3286	52.5	BoC07_36909176_301	BoC07	36909176
BnA02	bna3282	52.5	BoC07_37335580_301	BoC07	37335580
BnA02	bna3281	52.5	BoC07_37417143_299	BoC07	37417143
BnA02	bna5837	52.5	BoC07_37452969_301	BoC07	37452969
BnA02	bna3280	52.5	BoC07_37454319_301	BoC07	37454319
BnA02	bna3279	52.5	BoC07_37488062_301	BoC07	37488062
BnA02	bna3277	52.5	BoC07_37555131_301	BoC07	37555131
BnA02	bna0269	53.6	BoScaffold000013_P2_1538530_301		
BnA02	bna0870	53.6	BoScaffold000013_P2_1854300_287		
BnA02	bna4751	53.9	BoScaffold000180_336494_302		
BnA02	bna0265	53.9	BoScaffold000013_P2_2445372_302		
BnA02	bna0267	53.9	BoScaffold000013_P2_2007823_301		
BnA02	BGR52	55.3	BrA02_17526120_144		
BnA02	sR10417	55.3	BrA02_17125071_227		
BnA02	bna4720	55.6	BoC02_21299338_301	BoC02	21299338
BnA02	bna3533	56.7	BoC05_9768588_287		
BnA02	bna3531	56.7	BoC05_7783846_301		
BnA02	bna3528	56.7	BoC02_22655560_300	BoC02	22655560
BnA02	bna3529	56.7	BrA10_89244_100		
BnA02	bna5824	57.8	BoC01_11317620_301		
BnA02	bna3255	58.1	BoC02_23357144_301	BoC02	23357144
BnA02	bna3257	58.4	BoC02_23381964_301	BoC02	23381964
BnA02	bna5840	58.4	BoC06_1845858_301		
BnA02	bna3263	58.4	BrA05_8520929_145		
BnA02	bna3256	58.4	BoC03_32323085_302		
BnA02	bna3284	58.7	BoC09_27155188_301		
BnA02	bna0858	59	BoScaffold000393_56192_294		
BnA02	bna3298	59.3	BoC06_1908498_229		
BnA02	bna1031	59.3	BoScaffold000512_57088_141		
BnA02	bna3444	59.6	BoC02_25162106_301	BoC02	25162106
BnA02	bna3266	59.9	BoScaffold000335_300181_301		
BnA02	bna3272	59.9	BoScaffold000335_100012_301		
BnA02	bna3438	59.9	BoC02_25050175_278	BoC02	25050175
BnA02	bna3461	59.9	BoC06_14216429_301		
BnA02	bna5859	59.9	BoScaffold000003_P1_338520_297		
BnA02	bna3453	60.2	BoC07_25305432_301		
BnA02	bna0612	60.5	BoScaffold000233_755201_170		
BnA02	bna3258	60.5	BoC02_23426511_301	BoC02	23426511
BnA02	bna3269	60.5	BoScaffold000335_230394_301		
BnA02	bna4130	60.6	BoC08_9033619_300		
BnA02	bna5858	60.7	BrA03_21378186_130		

BnA02	bna3470	60.7	BoC03_41885114_301		
BnA02	bna3443	60.7	BoC02_25146228_302	BoC02	25146228
BnA02	bna4704	60.7	BoScaffold000419_82799_291		
BnA02	bna3492	60.7	BoC08_13180288_301		
BnA02	bna3449	60.8	BoC02_25222313_294	BoC02	25222313
BnA02	bna3446	60.9	BoC02_25244717_155	BoC02	25244717
BnA02	bna3450	60.9	BoC02_25309091_301	BoC02	25309091
BnA02	bna5969	60.9	BoC02_25568930_301	BoC02	25568930
BnA02	bna3451	60.9	BoC02_25351109_301	BoC02	25351109
BnA02	bna3452	60.9	BoC02_25442729_300	BoC02	25442729
BnA02	bna3456	60.9	BoC02_25719601_239	BoC02	25719601
BnA02	bna3457	60.9	BoC02_25741664_301	BoC02	25741664
BnA02	bna3460	60.9	BoC02_25833179_301	BoC02	25833179
BnA02	bna3462	60.9	BoC02_25869796_301	BoC02	25869796
BnA02	bna3463	61.2	BoC02_25884580_301	BoC02	25884580
BnA02	bna3467	61.2	BoC02_26044159_301	BoC02	26044159
BnA02	bna3468	61.2	BoC02_26067961_300	BoC02	26067961
BnA02	bna3267	61.2	BoScaffold000335_309595_223		
BnA02	bna3474	61.2	BoC02_26265513_301	BoC02	26265513
BnA02	bna3478	61.5	BoC02_26377496_250	BoC02	26377496
BnA02	bna3479	61.8	BoC02_26439434_295	BoC02	26439434
BnA02	bna3484	61.8	BoC02_26666221_258	BoC02	26666221
BnA02	bna3485	61.8	BoC02_26694404_299	BoC02	26694404
BnA02	bna3436	61.8	BoScaffold000031_P1_149878_301		
BnA02	bna3487	61.8	BoC02_26757922_301	BoC02	26757922
BnA02	bna3490	61.8	BoC02_26900603_297	BoC02	26900603
BnA02	bna3491	62.1	BoC02_26921898_264	BoC02	26921898
BnA02	bna4790	62.1	BoC02_27031205_301	BoC02	27031205
BnA02	bna3493	62.4	BoC02_27046229_278	BoC02	27046229
BnA02	bna3496	62.4	BoC02_27104488_302	BoC02	27104488
BnA02	bna3498	62.7	BoC02_27193907_301	BoC02	27193907
BnA02	bna3965	63	BoC02_28292620_301	BoC02	28292620
BnA02	bna3964	63	BoC02_28311847_301	BoC02	28311847
BnA02	bna3963	63.3	BoC02_28352422_239	BoC02	28352422
BnA02	bna3962	63.6	BoC02_28386263_301	BoC02	28386263
BnA02	bna3960	63.6	BoC02_28444056_301	BoC02	28444056
BnA02	bna3959	63.6	BoC02_28482686_301	BoC02	28482686
BnA02	bna3957	63.6	BoC02_28752066_301	BoC02	28752066
BnA02	bna3956	63.6	BoC02_28779755_301	BoC02	28779755
BnA02	bna3955	63.6	BoC02_28805541_301	BoC02	28805541
BnA02	bna1021	63.6	BoC02_28822177_301	BoC02	28822177
BnA02	bna3958	63.6	BoC02_28569158_301	BoC02	28569158
BnA02	bna3240	63.6	BoC02_29075223_301	BoC02	29075223
BnA02	bna3552	66.6	BoC02_29369413_301	BoC02	29369413
BnA02	bna0519	66.6	BoC07_18065200_298		
BnA02	bna0846	69.9	BoC02_29637108_269	BoC02	29637108
BnA02	bna0734	69.9	BoC02_29924834_311	BoC02	29924834

BnA02	bna4706	69.9	BoC02_31474599_301	BoC02	31474599
BnA02	bna3549	69.9	BoC07_32620548_299		
BnA02	bna3551	69.9	BoC02_31483657_301	BoC02	31483657
BnA02	bna4705	69.9	BoC02_31504241_301	BoC02	31504241
BnA02	bna3548	69.9	BoC02_31576602_301	BoC02	31576602
BnA02	bna3546	70.2	BoC02_31630315_301	BoC02	31630315
BnA02	bna3544	70.3	BoC02_31689351_301	BoC02	31689351
BnA02	bna3541	70.4	BoC02_31762247_301	BoC02	31762247
BnA02	bna3539	70.5	BoC02_31838722_301	BoC02	31838722
BnA02	bna3536	70.6	BoC02_31889051_301	BoC02	31889051
BnA02	bna3535	70.9	BoC02_31914762_301	BoC02	31914762
BnA02	bna0621	71.2	BoC02_35051270_295	BoC02	35051270
BnA02	bna1053	71.3			
BnA02	bna4764	71.4	BoC05_8530889_302		
BnA02	bna0540	71.7	BoC02_35600452_301	BoC02	35600452
BnA02	BGO153	73.4	BrA02_22858298_238		
BnA02	bna0587	77.7	BoC02_36701272_301	BoC02	36701272
BnA02	bna0743	80.7	BoC02_43650063_300		
BnA02	BEN189A	81.6			
BnA02	CB10022B	91			
BnA02	BrGMS363	92.4	BrA02_27738746_153		
BnA02	bna1514	92.7	BrA02_27812564_301		
BnA02	bna3571	93.8	BoC02_41705763_301	BoC02	41705763
BnA02	bna3565	93.8	BoScaffold000121_P2_789519_301		
BnA02	bna3564	93.8	BoScaffold000121_P2_775141_301		
BnA02	bna3568	93.8	BrA02_27439547_301		
BnA02	bna3569	93.8	BoC02_41757926_287	BoC02	41757926
BnA02	bna3566	96.8	BoScaffold000514_31276_301		
BnA02	BEN349	99.2			
BnA03	sN2032	0			
BnA03	bna0051	0.3	BoScaffold000091_1664085_301		
BnA03	bna3560	0.3	BoScaffold000407_6294_301		
BnA03	bna0288	0.3	BoC03_32383728_301	BoC03	32383728
BnA03	bna3791	3.6	BoC03_39863170_301	BoC03	39863170
BnA03	bna3790	3.6	BoC03_39888365_301	BoC03	39888365
BnA03	bna5949	3.6	BoC03_40197638_301	BoC03	40197638
BnA03	CALSSRB	3.6	BoC03_40355283_138	BoC03	40355283
BnA03	bna3780	3.6	BoC03_40477244_304	BoC03	40477244
BnA03	bna3789	3.6	BrA06_10261885_283		
BnA03	bna3782	3.6	BrA08_17627997_303		
BnA03	bna3778	3.6	BoC03_40501352_301	BoC03	40501352
BnA03	bna3775	3.6	BoC03_40591901_303	BoC03	40591901
BnA03	BoGMS819	6.2			
BnA03	BGO120	10.1	BoC03_43720660_235	BoC03	43720660
BnA03	bna0738	10.4	BoC06_4218118_188		
BnA03	bna0639	11	BoC03_43830330_301	BoC03	43830330
BnA03	BoGMS680	14.6			

BnA03	bna0583	15.5	BoC03_44543356_301	BoC03	44543356
BnA03	bna2666	15.8	BrA08_16479209_192		
BnA03	bna5138	15.8	BrA08_16478870_298		
BnA03	bna3811	16.4	BoC03_45939776_300	BoC03	45939776
BnA03	BoGMS154	16.7			
BnA03	bna3783	18.4	BoC03_40257094_301		
BnA03	bna3810	18.4	BoC03_46851701_301	BoC03	46851701
BnA03	bna3807	19.8	BoC03_47298054_301	BoC03	47298054
BnA03	bna3806	19.8	BoC03_47311414_301	BoC03	47311414
BnA03	bna4714	19.8	BoC03_47341746_301	BoC03	47341746
BnA03	bna3805	19.8	BoC03_47417434_295	BoC03	47417434
BnA03	bna4713	19.8			
BnA03	bna3804	19.8	BoScaffold000009_P1_1530670_280		
BnA03	bna3802	19.8	BoC03_47584059_280	BoC03	47584059
BnA03	bna0078	19.8	BoC03_47610468_156	BoC03	47610468
BnA03	bna4962	19.8	BoC03_47616595_270	BoC03	47616595
BnA03	bna0146	19.8	BoC03_47617425_301	BoC03	47617425
BnA03	BoGMS1295	20.1			
BnA03	bna3801	20.4	BoC03_47620248_301	BoC03	47620248
BnA03	bna3800	20.4	BoC03_47645315_307	BoC03	47645315
BnA03	bna3394	39	BoScaffold000098_115349_301		
BnA03	BEN123	39.6	BrA03_6449351_263		
BnA03	BN12AB	40.2	BrA03_6265845_277		
BnA03	bna3612	41.1	BoC03_7268049_303	BoC03	7268049
BnA03	bna3613	41.1	BrA03_6261112_302		
BnA03	bna3614	41.4	BoC03_7073696_301	BoC03	7073696
BnA03	bna4964	41.4	BoC03_7009435_301	BoC03	7009435
BnA03	bna3615	41.4	BoC03_6946602_301	BoC03	6946602
BnA03	bna3616	41.4	BoC03_6931807_301	BoC03	6931807
BnA03	bna3627	43.7	BoC03_6535141_301	BoC03	6535141
BnA03	bna3628	43.7	BoC03_6513447_305	BoC03	6513447
BnA03	bna3629	43.7	BoC03_6491259_301	BoC03	6491259
BnA03	BEN190A	46.3	BoC03_6271105_286	BoC03	6271105
BnA03	Na14-E02	47.4			
BnA03	Ol10-D03C	55.2			
BnA03	bna3582	59.8	BrA03_4102152_300		
BnA03	bna3603	60.1	BoC03_4914984_300	BoC03	4914984
BnA03	bna3588	60.1	BoC03_4552667_284	BoC03	4552667
BnA03	bna3593	61	BoC03_4170187_301	BoC03	4170187
BnA03	BoGMS1464A	62.1			
BnA03	BGO125	62.1	BoC03_3682470_273	BoC03	3682470
BnA03	bna5036	63.5	BoC03_3574955_301	BoC03	3574955
BnA03	bna1289	65.5	BoC01_20781518_301		
BnA03	bna5481	65.5	BoC03_2042670_301	BoC03	2042670
BnA03	bna3689	66.6	BoC03_1724642_215	BoC03	1724642
BnA03	bna3688	66.6	BoC03_1704921_301	BoC03	1704921
BnA03	bna3685	68.9	BrA03_1485228_301		

BnA03	bna0107	70.3	BoC03_24221_231	BoC03	24221
BnA03	bna0198	71.4	BrA03_135000_254		
BnA03	bna0197	71.4	BrA03_80381_301		
BnA03	BRMS-008	72.3	BrA03_53344_165		
BnA03	BrGMS679	74	BrA03_43587_209		
BnA03	BnEMS993	76.1			
BnA03	BRMS-006	78.4			
BnA03	BoGMS1320	83			
BnA03	BoGMS1201	85.7			
BnA03	BoGMS1373	86.3			
BnA03	sR12137IA	88.2	BrA03_1699524_355		
BnA03	BoGMS1307	92.1			
BnA03	CB10413	113.8	BrA03_28648208_215	BrA03	28648208
BnA03	BGR54	115.8	BrA03_28268108_129	BrA03	28268108
BnA03	bna1538	116.4	BrA03_28222246_301	BrA03	28222246
BnA03	bna1542	116.4	BrA03_27821377_289	BrA03	27821377
BnA03	bna1545	116.4	BrA03_27612941_301	BrA03	27612941
BnA03	bna1546	116.7	BrA03_27561648_301	BrA03	27561648
BnA03	bna1547	116.7	BrA03_27455080_301	BrA03	27455080
BnA03	bna1548	116.7	BrA03_27445975_306	BrA03	27445975
BnA03	bna5278	117	BrA03_27399085_301	BrA03	27399085
BnA03	bna1549	117	BrA03_27385091_301	BrA03	27385091
BnA03	BrGMS509	117.3	BrA03_27239138_278	BrA03	27239138
BnA03	bna5279	117.6	BrA03_27181901_301	BrA03	27181901
BnA03	bna1551	117.6	BrA03_27104883_301	BrA03	27104883
BnA03	bna1552	118.7	BrA03_26637960_298	BrA03	26637960
BnA03	bna1553	118.7	BrA03_26608007_293	BrA03	26608007
BnA03	bna1802	138.5	BrA03_501255_301	BrA03	501255
BnA03	Na10-G10	139.1	BrA03_672353_135	BrA03	672353
BnA03	bna5585	141.1	BrA03_1596241_300	BrA03	1596241
BnA03	sR12137IB	141.4	BrA03_1699524_355	BrA03	1699524
BnA03	BEN320	142	BoC03_1630224_238		
BnA03	BoGMS539	144	BrA03_1821839_183	BrA03	1821839
BnA03	BGO029	146	BrA03_3156872_138	BrA03	3156872
BnA03	BoGMS1464B	146.6			
BnA03	bna1748	148.9	BrA03_4257090_301	BrA03	4257090
BnA03	bna5581	148.9	BrA03_4339043_301	BrA03	4339043
BnA03	bna1747	148.9	BrA03_4399435_301	BrA03	4399435
BnA03	bna1743	151.2	BrA03_5006694_301	BrA03	5006694
BnA03	bna1741	151.2	BrA03_5083348_301	BrA03	5083348
BnA03	bna0462	151.2	BoC03_5872410_301		
BnA03	bna1740	151.2	BrA03_5160080_301	BrA03	5160080
BnA03	BEN190B	152.6	BoC03_6271105_286		
BnA03	bna1738	153.2	BrA03_5415096_301	BrA03	5415096
BnA03	bna1736	153.2	BrA03_5523389_301	BrA03	5523389
BnA03	bna1730	153.8	BrA03_6018284_301	BrA03	6018284
BnA03	BN12AA	154.7	BrA03_6265845_277	BrA03	6265845

BnA03	BEN251	154.7	BrA03_6283089_162	BrA03	6283089
BnA03	bna1727	155.3	BrA03_6286513_301	BrA03	6286513
BnA03	BnEMS1084	157	BrA03_6289126_377	BrA03	6289126
BnA03	bna5580	158.7	BrA03_6423348_301	BrA03	6423348
BnA03	bna1722	159	BrA03_6758687_302	BrA03	6758687
BnA03	bna1717	159.6	BrA03_7094848_303	BrA03	7094848
BnA03	bna1716	159.6	BrA03_7178701_155	BrA03	7178701
BnA03	bna1715	159.6	BrA03_7225004_272	BrA03	7225004
BnA03	bna1713	159.6	BrA03_7284240_301	BrA03	7284240
BnA03	BGR80	161.9	BrA03_7377352_124	BrA03	7377352
BnA03	bna1712	163	BrA03_7521574_301	BrA03	7521574
BnA03	bna1711	163	BrA03_7618218_224	BrA03	7618218
BnA03	bna1709	163.3	BrA03_7766266_309	BrA03	7766266
BnA03	bna5064	163.3	BrA03_7793224_299	BrA03	7793224
BnA03	bna1706	163.3	BrA03_7935716_301	BrA03	7935716
BnA03	Na12-E02A	163.9	BrA03_7953653_103	BrA03	7953653
BnA03	bna0092	165.3	BrA03_8627204_301	BrA03	8627204
BnA03	bna1842	165.6	BrA03_8940224_301	BrA03	8940224
BnA03	BGR75	166.7	BrA03_8963189_141	BrA03	8963189
BnA03	bna5284	170.6	BrA03_9243550_307	BrA03	9243550
BnA03	bna1846	170.6	BrA03_9250681_288	BrA03	9250681
BnA03	bna1847	170.6	BrA03_9258827_301	BrA03	9258827
BnA03	bna1849	170.6	BoC03_10808656_301		
BnA03	bna5880	170.6	BrA03_9382131_301	BrA03	9382131
BnA03	bna1851	171.2	BrA03_9627215_301	BrA03	9627215
BnA03	bna5590	171.2	BrA03_9641522_301	BrA03	9641522
BnA03	bna1852	171.2	BrA03_9657714_299	BrA03	9657714
BnA03	bna5577	172.1	BrA03_10455016_301	BrA03	10455016
BnA03	bna1692	173	BrA03_10716163_301	BrA03	10716163
BnA03	bna1691	173	BrA03_10761610_301	BrA03	10761610
BnA03	bna0057	173	BrA03_10900451_301	BrA03	10900451
BnA03	bna1686	173	BrA03_10969259_301	BrA03	10969259
BnA03	BRAS087B	176.8	BoC03_16290437_171		
BnA03	BRAS051B	178			
BnA03	BEN133	178.6	BrA03_11054113_157	BrA03	11054113
BnA03	bna1685	179.5	BrA03_11070726_301	BrA03	11070726
BnA03	bna1683	179.5	BrA03_11218618_301	BrA03	11218618
BnA03	bna5575	179.5	BrA03_11319872_301	BrA03	11319872
BnA03	bna1681	179.5	BrA03_11365646_301	BrA03	11365646
BnA03	bna1678	179.5	BrA03_11499674_302	BrA03	11499674
BnA03	bna1682	179.5	BrScaffold000167_105752_301		
BnA03	bna1677	179.5	BrA03_11531312_301	BrA03	11531312
BnA03	bna4986	179.5	BrA03_11534180_301	BrA03	11534180
BnA03	bna0238	179.5	BoC03_14372308_301		
BnA03	bna1676	179.5	BrA03_11593119_301	BrA03	11593119
BnA03	bna1674	179.5	BrA03_11685935_300	BrA03	11685935
BnA03	bna1673	180.1	BrA03_12316922_301	BrA03	12316922

BnA03	bna1232	180.1	BrA03_12548931_305	BrA03	12548931
BnA03	bna1670	180.4	BrA03_12932549_301	BrA03	12932549
BnA03	bna1669	180.4	BrA03_12933240_294	BrA03	12933240
BnA03	bna4985	180.4	BrA03_12990760_301	BrA03	12990760
BnA03	bna1667	180.4	BrA03_13160678_301	BrA03	13160678
BnA03	bna1665	180.4	BrA03_13257273_260	BrA03	13257273
BnA03	bna2170	180.4	BrA05_3828439_301		
BnA03	bna4844	180.4	BrA03_13298884_301	BrA03	13298884
BnA03	bna1663	180.4	BrA03_13365862_301	BrA03	13365862
BnA03	bna1662	180.4	BrA03_13425088_301	BrA03	13425088
BnA03	bna1660	180.4	BrA03_13579698_250	BrA03	13579698
BnA03	bna1659	181.5	BrA03_13658610_263	BrA03	13658610
BnA03	bna5446	181.5	BrA03_13678885_301	BrA03	13678885
BnA03	bna5903	181.5	BrA07_6044789_301		
BnA03	bna1658	181.5	BrA03_13707058_303	BrA03	13707058
BnA03	bna1656	181.5	BoC03_18995783_301		
BnA03	bna1650	182.9	BrA03_14292099_301	BrA03	14292099
BnA03	BEN25	186.2	BrA03_14628164_132	BrA03	14628164
BnA03	bna1643	188.5	BoScaffold000040_1433582_301		
BnA03	bna1638	189.6	BrA03_15368607_301	BrA03	15368607
BnA03	bna1637	189.6	BrA03_15421652_301	BrA03	15421652
BnA03	BoGMS1425	190.5			
BnA03	BrGMS216	191.9	BrA03_16125257_283	BrA03	16125257
BnA03	BrGMS217	193.1	BrA03_16137511_129	BrA03	16137511
BnA03	BGR35	196.8	BrA03_16137523_113	BrA03	16137523
BnA03	bna1631	199.6	BrA03_16229444_301	BrA03	16229444
BnA04	bna0702	0	BrA01_22363559_293		
BnA04	bna0817	0.6	BoC04_27594573_294		
BnA04	bna0556	3.2	BoC08_35189595_301		
BnA04	sN13034	3.8	BrA04_6624964_137		
BnA04	BRAS021	5			
BnA04	Na10-C01A	7.6			
BnA04	BoGMS829	7.6			
BnA04	bna2052	8.2	BoC04_29405992_301		
BnA04	bna0697	8.2	BoC04_29321703_301		
BnA04	BnEMS1184	32	BrA04_276812_389	BrA04	276812
BnA04	bna1810	33.1	BrA04_600073_301	BrA04	600073
BnA04	bna1822	35.7	BrA04_1457537_301	BrA04	1457537
BnA04	bna1827	37.1	BrA04_1735132_301	BrA04	1735132
BnA04	bna1828	37.1	BrA04_1779999_301	BrA04	1779999
BnA04	bna5083	37.1	BrA04_1894868_214	BrA04	1894868
BnA04	bna1830	37.1	BrA04_1943599_301	BrA04	1943599
BnA04	bna1832	37.4	BrA04_2048437_301	BrA04	2048437
BnA04	bna5913	37.7	BrA04_2199893_301	BrA04	2199893
BnA04	bna4845	37.7	BrA04_2230696_301	BrA04	2230696
BnA04	bna1834	37.7	BrA04_2309487_289	BrA04	2309487
BnA04	bna5592	38.6	BrA04_2497900_301	BrA04	2497900

BnA04	bna1860	38.9	BrA04_2650277_301	BrA04	2650277
BnA04	bna1861	39.5	BrA04_2688974_301	BrA04	2688974
BnA04	bna5593	39.5	BrA04_2735498_301	BrA04	2735498
BnA04	bna5196	39.5	BrA04_2933073_218	BrA04	2933073
BnA04	bna1865	39.5	BrA04_2968931_301	BrA04	2968931
BnA04	sR9411	40.9			
BnA04	bna5868	45.2	BrA04_4112310_301	BrA04	4112310
BnA04	bna5595	45.2	BrA04_4259512_185	BrA04	4259512
BnA04	bna1876	45.2	BrA04_4263813_301	BrA04	4263813
BnA04	bna5342	45.2	BoC04_7704927_262		
BnA04	BGR98	46.3	BoC04_7807666_118		
BnA04	CB10347	46.6	BrA04_4267194_184	BrA04	4267194
BnA04	bna5881	47.5	BrA06_5428029_222		
BnA04	bna1885	47.5	BrA04_4823706_301	BrA04	4823706
BnA04	bna1884	47.5	BrA04_4932863_301	BrA04	4932863
BnA04	BnEMS1116	50.8	BrA04_5237214_137	BrA04	5237214
BnA04	bna1882	54.1	BrA04_5287304_301	BrA04	5287304
BnA04	bna1888	55	BrA04_5985363_301	BrA04	5985363
BnA04	bna1893	56.1	BrA04_7035364_301	BrA04	7035364
BnA04	bna1895	56.4	BrA04_7233828_301	BrA04	7233828
BnA04	bna1900	56.4	BrA04_7520180_301	BrA04	7520180
BnA04	bna1901	56.4	BrA04_7588670_301	BrA04	7588670
BnA04	bna1894	56.4	BrA08_19316263_301		
BnA04	bna3976	57.2	BoScaffold000192_615990_301		
BnA04	bna5380	57.2	BrA04_7643434_301	BrA04	7643434
BnA04	BEN98	58.3	BrA04_7920797_150	BrA04	7920797
BnA05	BnGMS584B	0	BrA03_20134626_223		
BnA05	BGR74	2.7	BrA03_20135054_129		
BnA05	Ra3-D04	3.3			
BnA05	BrGMS406	3.3	BrA03_18621814_333	BrA03	18621814
BnA05	Na10-A09	3.3			
BnA05	bna1616	3.9	BrA03_18834614_301	BrA03	18834614
BnA05	bna1615	3.9	BrA03_18836652_294	BrA03	18836652
BnA05	bna1617	3.9	BoC06_34818725_300		
BnA05	bna1613	4.2	BoC03_25470736_300		
BnA05	bna1608	4.5	BrA03_20080950_301	BrA03	20080950
BnA05	bna1601	5.1	BrA03_20667595_269	BrA03	20667595
BnA05	bna5566	9	BrA03_21198346_301	BrA03	21198346
BnA05	bna4349	9.6	BoC06_37133106_225		
BnA05	bna1599	10.2	BrA03_21504721_301	BrA03	21504721
BnA05	bna1590	13.5	BrA03_22300522_303	BrA03	22300522
BnA05	CB10034	16.8	BoC06_38415389_93		
BnA05	bna2843	20.1	BrA03_23127508_301	BrA03	23127508
BnA05	bna5886	20.3	BrA03_23160521_301	BrA03	23160521
BnA05	bna1589	20.4	BrA03_23467471_300	BrA03	23467471
BnA05	bna4642	20.4	BrA03_23503230_234	BrA03	23503230
BnA05	bna5911	20.4	BoC06_41088998_252		

BnA05	bna0038	20.4	BrA03_23526906_301	BrA03	23526906
BnA05	bna0037	20.4	BrA03_23528679_301	BrA03	23528679
BnA05	bna1585	20.4	BrA03_23800118_301	BrA03	23800118
BnA05	bna4840	21.5	BrA03_23887523_299	BrA03	23887523
BnA05	bna1584	22.4	BrA03_24365533_301	BrA03	24365533
BnA05	bna1575	23	BrA03_25047900_301	BrA03	25047900
BnA05	pMR181B	23.9			
BnA05	BEN83	25	BoC06_43435020_259		
BnA05	BnEMS146	25	BrA03_25526885_250	BrA03	25526885
BnA05	BGR56	25	BoC06_43222338_105		
BnA05	bna1572	25.3	BrA03_25340743_301	BrA03	25340743
BnA05	bna5121	25.3	BrA03_25340743_301	BrA03	25340743
BnA05	bna5097	25.3	BrA03_25499459_223	BrA03	25499459
BnA05	bna4838	25.3	BrA03_25504906_301	BrA03	25504906
BnA05	bna4389	25.6	BoC06_43594658_301		
BnA05	bna1571	25.6	BrA03_25614556_301	BrA03	25614556
BnA05	bna1567	25.6	BoC06_43730699_301		
BnA05	bna0021	26.4	BrA03_26163719_301	BrA03	26163719
BnA05	bna5444	27.3	BrA03_26272710_301	BrA03	26272710
BnA05	BnGMS265	43.6	BrA05_22210227_345	BrA05	22210227
BnA05	sR9477	44.8	BrA05_22498503_263	BrA05	22498503
BnA05	bna2038	45.7	BrA05_22292108_301	BrA05	22292108
BnA05	bna5616	46.3	BrA05_22427562_301	BrA05	22427562
BnA05	bna2037	46.3	BrA05_22427562_301	BrA05	22427562
BnA05	BGR38	48	BrA05_22561185_146	BrA05	22561185
BnA05	bna2035	48.6	BrA05_22580798_289	BrA05	22580798
BnA05	bna5092	48.6	BrA05_22631736_297	BrA05	22631736
BnA05	bna2033	49.5	BrA05_22766863_301	BrA05	22766863
BnA05	bna2028	50.1	BrA05_23020982_301	BrA05	23020982
BnA05	bna1476	50.7	BrA02_23247623_301		
BnA05	bna5091	50.7	BoC05_32701848_301	BoC05	32701848
BnA05	bna2029	51.3	BoC05_32515770_301	BoC05	32515770
BnA05	bna5450	51.6	BoC05_32350689_300	BoC05	32350689
BnA05	bna2030	51.6	BoC05_32350587_297	BoC05	32350587
BnA05	bna5288	52.5	BrA05_23082022_302	BrA05	23082022
BnA05	bna2026	52.8	BrA05_23162355_301	BrA05	23162355
BnA05	bna5090	53.4	BrA05_23207558_245	BrA05	23207558
BnA05	BRMS-007	54.5	BrA05_23268922_132	BrA05	23268922
BnA05	bna5615	55.6	BrA05_23385467_301	BrA05	23385467
BnA05	bna2024	55.9	BrA05_23425922_301	BrA05	23425922
BnA05	bna2025	56.5	BrA05_23502843_301	BrA05	23502843
BnA05	BoGMS1059	58.2			
BnA05	bna1515	59.3	BrA02_26946155_301		
BnA05	bna5991	59.3	BoScaffold000460_96754_301		
BnA05	bna0286	59.4	BrScaffold000191_59457_298		
BnA05	bna0306	59.5	BrScaffold000203_24232_301		
BnA05	bna1516	59.8	BoScaffold000460_119035_272		

BnA05	bna5371	59.8	BoScaffold000461_93279_301		
BnA05	bna0321	61.1	BrA02_26686182_208		
BnA05	bna1511	61.5	BrA02_26644147_301		
BnA05	bna2023	62.6	BrA05_23533846_301	BrA05	23533846
BnA05	BEN200A	63.2	BrA05_23831506_214	BrA05	23831506
BnA06	bna5650	0	BrA06_2993650_301	BrA06	2993650
BnA06	bna5651	0.3	BrA06_3114668_301	BrA06	3114668
BnA06	bna4853	0.3	BrA06_3203762_301	BrA06	3203762
BnA06	bna0130	0.3	BrA06_3279046_301	BrA06	3279046
BnA06	bna2267	0.3	BrA06_3279046_301	BrA06	3279046
BnA06	sN2837	3.6	BrA06_3578834_153	BrA06	3578834
BnA06	bna5251	4.2	BrA06_3651623_301	BrA06	3651623
BnA06	bna2259	4.2	BrA06_3651623_301	BrA06	3651623
BnA06	Na12-D08	5.1	BrA06_3726880_69	BrA06	3726880
BnA06	bna5040	5.7	BrA06_3867444_242	BrA06	3867444
BnA06	bna2254	5.7	BrA06_3867444_242	BrA06	3867444
BnA06	bna5392	6.8	BrA06_4026624_322	BrA06	4026624
BnA06	bna2251	6.8	BrA06_4035954_301	BrA06	4035954
BnA06	Ol10-D01	8.2	BrA06_4399748_208	BrA06	4399748
BnA06	bna2247	9.9	BrA06_4534080_301	BrA06	4534080
BnA06	bna2245	9.9	BoScaffold000127_203799_227		
BnA06	bna5455	9.9	BoScaffold000127_203799_227		
BnA06	bna4062	9.9	BoC05_3725941_301		
BnA06	bna2246	9.9	BrA06_4738919_263	BrA06	4738919
BnA06	bna5657	10.2	BrA06_5577784_301	BrA06	5577784
BnA06	bna5887	10.5	BrA09_16121849_288		
BnA06	bna5932	10.5	BrA09_16121849_288		
BnA06	bna2279	11.6	BrA06_6520150_300	BrA06	6520150
BnA06	bna5343	12.5	BrA06_6691478_301	BrA06	6691478
BnA06	bna5658	12.8	BrA06_6878642_301	BrA06	6878642
BnA06	BEN253	16.4	BrA06_9151367_207	BrA06	9151367
BnA06	bna5132	17	BrA06_10134979_311	BrA06	10134979
BnA06	bna1897	17	BrScaffold000178_232155_303		
BnA06	bna3792	17	BoC03_39765391_301		
BnA06	bna3796	17	BoC03_39601720_301		
BnA06	bna5781	17.3	BrScaffold000244_26717_302		
BnA06	bna0279	17.3	BrScaffold000796_970_278		
BnA06	bna4342	17.3	BrA06_11667624_301	BrA06	11667624
BnA06	bna5142	17.6	BrA06_19593663_299	BrA06	19593663
BnA06	bna0335	17.6	BrA06_19609664_301	BrA06	19609664
BnA06	BGR34	18.2	BrA06_11864505_137	BrA06	11864505
BnA06	BRMS-049A	18.5	BrA06_12580768_141	BrA06	12580768
BnA06	BoGMS1203	19.6			
BnA06	BoGMS1230	23.9			
BnA06	bna2318	29.2	BrA06_14265955_298	BrA06	14265955
BnA06	bna4958	29.2	BrA06_14594986_291	BrA06	14594986
BnA06	bna2296	33.8	BrA06_16620834_301	BrA06	16620834

BnA06	bna2297	34.1	BrA06_16659915_301	BrA06	16659915
BnA06	bna2580	34.7	BrA07_1396686_301		
BnA06	bna2311	37.7	BoScaffold000153_664915_261		
BnA06	bna2305	40.4	BrA06_17822965_301	BrA06	17822965
BnA06	BGR45	41.3	BrA06_17992203_105	BrA06	17992203
BnA06	BoGMS314	41.6	BrA06_18069372_115	BrA06	18069372
BnA06	bna2307	42.2	BrA06_18116757_177	BrA06	18116757
BnA06	bna4990	42.2	BoC06_35346921_292		
BnA06	bna5395	42.5	BoScaffold000153_664915_261		
BnA06	bna5898	42.5	BrA06_18657413_302	BrA06	18657413
BnA06	bna5877	42.5	BrA06_18657413_302	BrA06	18657413
BnA06	bna2312	42.8	BrA06_18772227_301	BrA06	18772227
BnA06	bna2339	43.7	BrA06_21171937_301	BrA06	21171937
BnA06	bna4655	44	BrA06_21247253_219	BrA06	21247253
BnA06	bna5665	44.3	BrA06_21475708_303	BrA06	21475708
BnA06	bna2336	44.3	BrA06_21512860_268	BrA06	21512860
BnA06	bna5922	46	BrA06_21676878_301	BrA06	21676878
BnA06	BnEMS59	47.4	BrA06_21942889_171	BrA06	21942889
BnA06	BnEMS60	47.7	BrA06_21942889_171	BrA06	21942889
BnA06	bna2342	51	BrA06_22104886_300	BrA06	22104886
BnA06	bna5298	57	BrA06_22807945_301	BrA06	22807945
BnA06	bna2344	57	BrA06_22807945_301	BrA06	22807945
BnA06	bna2346	57	BrA06_22896930_301	BrA06	22896930
BnA06	sR12156A	58.1	BrA06_23032511_177	BrA06	23032511
BnA06	bna5923	58.7	BrA06_23656979_285	BrA06	23656979
BnA06	bna2358	59.3	BrA06_24695710_301	BrA06	24695710
BnA06	bna0629	59.3	BoC06_27820808_301		
BnA06	BEN278B	59.9	BoC06_29189191_152		
BnA06	bna2364	64.2	BrA06_26039984_299	BrA06	26039984
BnA06	bna5347	64.2	BrA06_26039984_299	BrA06	26039984
BnA06	CB10330	65.4	BrA06_26104700_148	BrA06	26104700
BnA06	BnEMS695	66.4	BrA06_26241942_212	BrA06	26241942
BnA07	BEN200B	0			
BnA07	bna1214	0.6	BrA04_9976097_301		
BnA07	bna0860	0.6	BoScaffold000485_60469_265		
BnA07	BEN186	1.7			
BnA07	bna0837	2	BoC05_32800593_301		
BnA07	bna4140	2.3	BoScaffold000461_38186_301		
BnA07	bna0084	2.3	BoC05_32774889_301		
BnA07	bna0882	2.3	BoScaffold000460_57388_301		
BnA07	bna0826	5.3	BoScaffold000462_60360_302		
BnA07	bna0771	25.7	BoC07_13343898_301	BoC07	13343898
BnA07	bna3841	26.6	BoC07_13552421_301	BoC07	13552421
BnA07	bna3149	35.5	BoC04_33944565_303		
BnA07	bna0896	35.8	BoScaffold000401_119918_239		
BnA07	bna0806	36.1	BoScaffold000401_176117_301		
BnA07	bna0594	36.4	BoC07_15896409_301	BoC07	15896409

BnA07	bna3875	37.8	BoC06_13001402_300		
BnA07	bna3878	37.8	BoScaffold000029_1537639_301		
BnA07	bna3877	37.8	BoScaffold000029_1577084_301		
BnA07	bna4817	37.8	BoScaffold000029_1654518_301		
BnA07	bna0351	37.8	BrA01_21633874_301		
BnA07	bna3876	37.8	BoScaffold000029_1736580_301		
BnA07	bna0035	39.5	BoScaffold000284_63051_301		
BnA07	bna0036	39.5	BoScaffold000284_63570_300		
BnA07	bna0517	39.5	BoScaffold000284_16130_301		
BnA07	bna4086	39.5	BoC03_33143147_301		
BnA07	Ol10-D03B	40.9	BoC07_18528915_106	BoC07	18528915
BnA07	bna0547	41.5	BoC07_18776685_301	BoC07	18776685
BnA07	bna4786	42.9	BrA05_11057670_301		
BnA07	bna0423	43.2	BoC07_20728474_301	BoC07	20728474
BnA07	BoGMS490	45.5	BoC07_21568379_300	BoC07	21568379
BnA07	bna4370	46.4	BoC06_41160354_281		
BnA07	bna0951	47.3	BoC07_21887638_301	BoC07	21887638
BnA07	BGO160B	49.3	BoC07_22121093_242	BoC07	22121093
BnA07	BGO161	49.6	BoC07_22141972_243	BoC07	22141972
BnA07	BEN391A	49.6	BoC07_22205936_234	BoC07	22205936
BnA07	bna0505	51.3	BoC07_24768318_301	BoC07	24768318
BnA07	bna0504	52.7	BoC07_25038543_302	BoC07	25038543
BnA07	bna1038	56.3	BoScaffold000009_P1_226299_301		
BnA07	bna3842	56.6	BoC07_25410267_301	BoC07	25410267
BnA07	bna5067	56.6	BoC07_25424488_301	BoC07	25424488
BnA07	bna4502	56.6	BoC07_25424488_301	BoC07	25424488
BnA07	bna3846	56.6	BoC07_25858003_301	BoC07	25858003
BnA07	bna3851	56.6	BoC07_26057612_301	BoC07	26057612
BnA07	bna0888	56.6	BoC07_26256585_301	BoC07	26256585
BnA07	bna0866	57.5	BoC07_40244944_301		
BnA07	BoGMS545	80.6	BrA07_277845_210	BrA07	277845
BnA07	J0609	80.9			
BnA07	BGO114	80.9			
BnA07	bna0323	81.5	BrA07_359031_301	BrA07	359031
BnA07	bna2369	81.5	BrA07_367720_301	BrA07	367720
BnA07	sR4047	85.1	BrA07_829294_270	BrA07	829294
BnA07	bna2411	86.5	BrA07_923958_207	BrA07	923958
BnA07	bna2410	86.8	BrA07_1197924_299	BrA07	1197924
BnA07	bna5003	87.1	BrA07_1300601_296	BrA07	1300601
BnA07	bna3045	87.1	BrA09_18277850_301		
BnA07	bna3044	87.1	BrA07_1372394_300	BrA07	1372394
BnA07	bna5002	87.4	BrA07_1595561_301	BrA07	1595561
BnA07	bna3040	87.4	BrA10_11548392_301		
BnA07	bna5056	87.4	BrA10_11839859_301		
BnA07	bna3036	87.4	BrA10_11872043_301		
BnA07	bna3043	87.4	BrA07_1603000_301	BrA07	1603000
BnA07	bna5888	87.4	BrA07_1734481_301	BrA07	1734481

BnA07	bna5264	87.4	BrA07_1766618_273	BrA07	1766618
BnA07	bna3128	87.4	BrA07_1766618_273	BrA07	1766618
BnA07	BoGMS1575	87.7			
BnA07	sR0282R	88			
BnA07	Na12-B02	89.4			
BnA07	bna5777	90.3	BrA10_3160721_301		
BnA07	bna3120	90.3	BrA10_3243515_298		
BnA07	bna2378	90.3	BrA08_9537088_276		
BnA07	bna3029	90.3	BrA09_19718503_279		
BnA07	bna5661	90.3	BrA10_3116354_268		
BnA07	bna2377	90.3	BrA07_3065564_282	BrA07	3065564
BnA07	bna2382	90.6	BrA07_3596136_301	BrA07	3596136
BnA07	bna2383	90.6	BrA07_3717065_301	BrA07	3717065
BnA07	bna2390	90.6	BoC06_3357873_302		
BnA07	bna2384	90.6	BrA07_3796323_302	BrA07	3796323
BnA07	bna2386	90.6	BrA07_3935049_301	BrA07	3935049
BnA07	bna2391	90.6	BrA07_4180352_301	BrA07	4180352
BnA07	bna2394	91.2	BrA07_4480120_291	BrA07	4480120
BnA07	BEN236	91.5	BrA07_4675434_199	BrA07	4675434
BnA07	Ra2-G08	91.5	BrA07_4761169_394	BrA07	4761169
BnA07	bna2396	91.8	BrA07_4911941_301	BrA07	4911941
BnA07	bna2400	91.8	BrA07_5089975_263	BrA07	5089975
BnA07	bna4856	92.1	BrA01_10550339_299		
BnA07	bna2404	92.1	BrA07_5367229_301	BrA07	5367229
BnA07	BrGMS38	93.1	BrA07_5615635_407	BrA07	5615635
BnA07	bna2416	94.7	BrA07_6248377_104	BrA07	6248377
BnA07	BEN45	95	BrA07_6280732_122	BrA07	6280732
BnA07	bna2418	95.3	BrA07_6360973_301	BrA07	6360973
BnA07	bna1324	96.2	BrA07_7009807_279	BrA07	7009807
BnA07	bna2432	97.1	BrA07_7562178_301	BrA07	7562178
BnA07	BGR20	97.7	BrA07_7572279_104	BrA07	7572279
BnA07	bna2433	98.6	BrA07_7610939_301	BrA07	7610939
BnA07	bna2434	98.6	BrA07_7635002_285	BrA07	7635002
BnA07	bna2435	98.9	BrA07_7752491_226	BrA07	7752491
BnA07	bna2436	98.9	BrA07_7874003_301	BrA07	7874003
BnA07	bna1477	99.2	BoC09_38401229_303		
BnA07	bna2452	99.8	BrA07_9030502_297	BrA07	9030502
BnA07	bna0287	100.7	BrA01_12936313_302		
BnA07	CB10299	101.8	BrA07_9168262_154	BrA07	9168262
BnA07	bna0132	102.9	BrA07_9841219_301	BrA07	9841219
BnA07	bna5173	102.9	BrA07_10076851_301	BrA07	10076851
BnA07	bna2462	102.9	BrA07_9867651_305	BrA07	9867651
BnA07	bna2464	102.9	BrA07_10009675_301	BrA07	10009675
BnA07	bna2465	102.9	BrA07_10070123_301	BrA07	10070123
BnA07	BEN288	105.9	BrA07_11178806_135	BrA07	11178806
BnA07	BEN178	106.8			
BnA07	bna2467	107.4	BoC06_647138_302		

BnA07	bna1305	108.5	BrA02_14309_301	BrA02	14309
BnA07	bna1307	108.5	BrA02_76135_272	BrA02	76135
BnA07	bna5521	109.9	BrA02_236832_302	BrA02	236832
BnA07	bna1312	110.2	BrA02_303906_301	BrA02	303906
BnA07	bna5368	110.2	BrA02_303906_301	BrA02	303906
BnA07	bna5597	110.8	BrA04_9074350_301		
BnA07	bna1315	110.8	BrA02_423955_211	BrA02	423955
BnA07	bna5524	110.8	BrA02_613245_301	BrA02	613245
BnA07	BEN254	111.7			
BnA07	bna1320	112	BrA02_647559_280	BrA02	647559
BnA07	bna5525	112	BrA02_770869_301	BrA02	770869
BnA07	bna1322	112	BrA02_810600_301	BrA02	810600
BnA07	bna2617	112	BrA02_6306888_301		
BnA07	bna5188	112.6	BrA07_11472403_301	BrA07	11472403
BnA07	bna2471	113.5	BrA07_11728297_301	BrA07	11728297
BnA07	bna2473	113.8	BrA07_11896650_302	BrA07	11896650
BnA07	bna2474	113.8	BrA07_11898064_287	BrA07	11898064
BnA07	bna2475	113.8	BrA07_11960515_301	BrA07	11960515
BnA07	sR7223	114.1	BrA07_12314707_413	BrA07	12314707
BnA07	Ol09-A06B	115			
BnA07	bna4662	116.7	BrA07_12361556_300	BrA07	12361556
BnA07	bna2483	116.7	BrA07_12411966_301	BrA07	12411966
BnA07	bna5682	116.7	BrA07_12511408_301	BrA07	12511408
BnA07	CB10439	117	BrA07_12579775_179	BrA07	12579775
BnA07	sNRA59	119.6	BrA07_13058693_125	BrA07	13058693
BnA07	bna2489	120.7	BrA07_13156451_301	BrA07	13156451
BnA07	bna0085	121	BrA07_13171897_301	BrA07	13171897
BnA07	bna2496	121	BrA07_13283149_301	BrA07	13283149
BnA07	bna2498	121	BrA07_13347565_301	BrA07	13347565
BnA07	bna2500	121	BrA07_13513700_301	BrA07	13513700
BnA07	bna2502	121	BrA07_13568545_301	BrA07	13568545
BnA07	BnGMS147A	121.3	BrA07_13588712_232	BrA07	13588712
BnA07	bna2503	121.6	BrA07_13642281_301	BrA07	13642281
BnA07	bna2504	121.6	BrA07_13654040_301	BrA07	13654040
BnA07	bna5926	121.6	BrA07_13674921_300	BrA07	13674921
BnA07	bna5302	121.9	BrA07_13840515_301	BrA07	13840515
BnA07	bna0086	121.9	BrA07_13936337_300	BrA07	13936337
BnA07	bna5045	121.9	BrA07_14002506_301	BrA07	14002506
BnA07	bna2510	121.9	BrA07_14002506_301	BrA07	14002506
BnA07	bna2512	121.9	BrA07_14125225_301	BrA07	14125225
BnA07	bna4821	121.9	BrA07_14326373_302	BrA07	14326373
BnA07	bna2514	121.9	BrA07_14388103_301	BrA07	14388103
BnA07	bna0167	121.9	BrA07_14594194_301	BrA07	14594194
BnA07	bna4778	121.9	BrA07_14596135_301	BrA07	14596135
BnA07	bna0168	121.9	BrA07_14599861_301	BrA07	14599861
BnA07	BGR100	122.2	BrA07_14656668_112	BrA07	14656668
BnA07	BGR99A	122.2	BrA07_14701381_148	BrA07	14701381

BnA07	bna2521	122.5	BrA07_14792117_299	BrA07	14792117
BnA07	bna2524	122.5	BrA07_14926716_301	BrA07	14926716
BnA07	bna2526	122.5	BrA07_15006833_301	BrA07	15006833
BnA07	BGO156B	122.8	BrA07_15030696_149	BrA07	15030696
BnA07	bna0169	123.1	BrA07_15353942_297	BrA07	15353942
BnA07	bna5112	123.1	BoScaffold000343_161218_301		
BnA07	bna5688	123.1	BrA07_15354338_301	BrA07	15354338
BnA07	bna0088	123.1	BrA07_15381136_300	BrA07	15381136
BnA07	bna2540	123.4	BrA07_16164923_302	BrA07	16164923
BnA07	Ol12-E03A	124.3	BrA07_16247167_117	BrA07	16247167
BnA07	bna2541	125.2	BrA07_16254256_301	BrA07	16254256
BnA07	FITO035B	126.1			
BnA07	bna2559	127.8	BrA07_17846151_301	BrA07	17846151
BnA07	bna2562	127.8	BrA07_18088001_301	BrA07	18088001
BnA07	bna0047	128.1	BrA07_18127466_299	BrA07	18127466
BnA08	bna5304	0	BrA08_759127_301	BrA08	759127
BnA08	bna2760	0	BrA08_914135_259	BrA08	914135
BnA08	bna5706	5.6	BrA08_979684_89	BrA08	979684
BnA08	BoGMS1375	8.2			
BnA08	BGR2	12.5	BrA08_1088421_148	BrA08	1088421
BnA08	bna2743	15.1	BrA08_2516101_301	BrA08	2516101
BnA08	bna0378	15.1	BrA08_2500830_301	BrA08	2500830
BnA08	bna0374	15.1	BrA05_15205515_301		
BnA08	bna5876	16	BrA08_2973687_301	BrA08	2973687
BnA08	bna5897	16	BrA08_2973687_301	BrA08	2973687
BnA08	bna5259	16.6	BrA08_3336709_303	BrA08	3336709
BnA08	bna2925	18	BrA08_4781235_301	BrA08	4781235
BnA08	bna2713	18	BrA08_5300196_301	BrA08	5300196
BnA08	bna4864	18	BrA08_5366755_298	BrA08	5366755
BnA08	bna5052	18	BrA08_5490202_302	BrA08	5490202
BnA08	bna5705	18	BrA08_5660516_305	BrA08	5660516
BnA08	bna2710	18	BrA08_5664464_294	BrA08	5664464
BnA08	BGR93	22.3	BrA08_5736292_113	BrA08	5736292
BnA08	BGR69	27.6	BrA08_6151459_136	BrA08	6151459
BnA08	bna0319	28.7	BrA08_7064446_301	BrA08	7064446
BnA08	bna0221	28.7	BrA08_7092588_301	BrA08	7092588
BnA08	bna5411	28.7	BoScaffold000064_262868_307		
BnA08	bna2759	28.7	BoScaffold000064_262868_307		
BnA08	bna2756	28.7	BrA08_7182928_304	BrA08	7182928
BnA08	bna0291	28.7	BoC08_9845211_301		
BnA08	bna0217	28.7	BrA08_7232063_301	BrA08	7232063
BnA08	bna2495	28.7	BoC04_17492930_266		
BnA08	bna1525	28.7	BrScaffold000180_112061_233		
BnA08	bna2752	28.7	BrA08_7444486_309	BrA08	7444486
BnA08	BGR61	29.3	BrA08_7614956_149	BrA08	7614956
BnA08	bna1296	29.9	BrA08_7698295_301	BrA08	7698295
BnA08	bna2704	29.9	BrA08_7717106_301	BrA08	7717106

BnA08	bna2707	30.2	BoC01_19271492_301		
BnA08	bna1941	30.2	BrA08_8200184_317	BrA08	8200184
BnA08	bna2596	30.5	BrA08_8479782_301	BrA08	8479782
BnA08	bna5303	30.5	BrA08_8495863_301	BrA08	8495863
BnA08	sS2331BB	31.1	BrA08_8588167_116	BrA08	8588167
BnA08	bna2598	31.7	BrA08_8589463_301	BrA08	8589463
BnA08	bna2599	31.7	BrA08_8721374_301	BrA08	8721374
BnA08	BGR1A	32.3	BrA08_8946388_150	BrA08	8946388
BnA08	bna5221	35.3	BrA08_10186467_301	BrA08	10186467
BnA08	bna2694	35.3	BrA08_10186467_301	BrA08	10186467
BnA08	BEN43	38.6	BrA08_10397240_227	BrA08	10397240
BnA08	BEN48	41.3	BrA08_10398587_201	BrA08	10398587
BnA08	FITO131	41.9			
BnA08	bna2699	43.3	BrA08_10711075_301	BrA08	10711075
BnA08	BGR8	44.4	BrA08_11358558_145	BrA08	11358558
BnA08	BRMS97	46.7	BrA08_14676090_194	BrA08	14676090
BnA08	bna2686	48.4	BoScaffold000375_162328_301		
BnA08	bna2687	48.4	BrA08_14729798_301	BrA08	14729798
BnA08	bna2677	49.8	BrA08_15726752_301	BrA08	15726752
BnA08	bna5699	49.8	BrA08_15726752_301	BrA08	15726752
BnA08	bna2672	50.7	BrA08_16033023_308	BrA08	16033023
BnA08	BEN116	51.3	BrA08_16117222_181	BrA08	16117222
BnA08	sR3688	51.9	BrA08_16205351_255	BrA08	16205351
BnA08	bna4993	52.5	BrA08_16248783_301	BrA08	16248783
BnA08	bna4861	55.2	BrA08_16652551_302	BrA08	16652551
BnA08	BGO006	57.2	BrA08_16900709_113	BrA08	16900709
BnA08	bna2662	58.1	BrA08_17000653_297	BrA08	17000653
BnA08	bna5856	58.1	BrA08_17050908_266	BrA08	17050908
BnA08	BGO120B	59	BrA08_17125566_219	BrA08	17125566
BnA08	bna2661	59.6	BrA08_17158805_304	BrA08	17158805
BnA08	bna2660	59.6	BrA08_17192991_301	BrA08	17192991
BnA08	bna2659	59.6	BrA08_17297854_301	BrA08	17297854
BnA08	bna5462	60.5	BrA08_17473735_301	BrA08	17473735
BnA08	bna2655	60.5	BrA08_17535008_301	BrA08	17535008
BnA08	bna2654	60.5	BrA08_17579140_301	BrA08	17579140
BnA08	bna2653	60.5	BrA08_17586824_299	BrA08	17586824
BnA08	bna2649	60.5			
BnA08	CALSSRA	61.1	BrA08_17620710_145	BrA08	17620710
BnA08	bna2652	61.7	BrA08_17642831_301	BrA08	17642831
BnA08	bna2650	61.7	BrA08_17858014_209	BrA08	17858014
BnA08	bna5404	61.7	BrA08_17858014_209	BrA08	17858014
BnA08	bna5218	67.7	BrA05_8139038_304		
BnA08	bna2623	67.7	BrA05_8139038_304		
BnA08	bna2621	68	BrA05_8223432_300		
BnA08	bna2619	68.9	BrA05_8318584_302		
BnA09	BEN332A	0	BrA09_936877_256	BrA09	936877
BnA09	BEN50B	0.9	BrA09_936928_277	BrA09	936928

BnA09	BEN50A	9.1	BrA09_936928_277	BrA09	936928
BnA09	BEN332B	9.1	BrA09_936877_256	BrA09	936877
BnA09	BN38A	10	BrA09_764538_156	BrA09	764538
BnA09	bna2964	18.2	BoC09_285052_304		
BnA09	BEN55B	19.1	BrA09_2356689_162	BrA09	2356689
BnA09	FITO135A	19.7			
BnA09	BrGMS725A	28.6	BrA09_2302831_166	BrA09	2302831
BnA09	BnGMS133	40.7	BrA09_4198837_248	BrA09	4198837
BnA09	BnEMS820A	47			
BnA09	sR9447	51.3	BrA09_4281574_349	BrA09	4281574
BnA09	sN1988	51.7	BrA09_3907955_277	BrA09	3907955
BnA09	bna2954	52.5	BrScaffold000178_33738_301		
BnA09	bna2953	52.8	BrScaffold000178_39098_249		
BnA09	bna2566	52.8	BrA09_4683310_301	BrA09	4683310
BnA09	bna5309	52.8	BrA09_4905856_301	BrA09	4905856
BnA09	bna5176	52.8	BrA09_4940739_301	BrA09	4940739
BnA09	bna5143	52.8	BrA09_5245915_301	BrA09	5245915
BnA09	bna2952	52.8	BrA09_5298231_301	BrA09	5298231
BnA09	bna2955	53.1	BrA09_4357188_170	BrA09	4357188
BnA09	bna0274	59.4	BrA09_6783993_301	BrA09	6783993
BnA09	bna2934	59.4	BrA09_6805269_300	BrA09	6805269
BnA09	bna0725	62.4	BoScaffold000215_564770_302		
BnA09	BrGMS316	63.5	BrA09_9108578_310	BrA09	9108578
BnA09	bna2920	64.4	BrA09_9447053_301	BrA09	9447053
BnA09	bna5873	64.4	BrA09_9452991_253	BrA09	9452991
BnA09	bna0498	65	BoC08_1052043_301		
BnA09	bna4685	65.3	BrA09_9735024_301	BrA09	9735024
BnA09	bna5419	65.3	BrA09_9724424_301	BrA09	9724424
BnA09	bna2916	65.6	BrA09_9845178_301	BrA09	9845178
BnA09	bna2902	65.6	BrA09_13737008_301	BrA09	13737008
BnA09	BEN284	66.5	BrA09_13822937_96	BrA09	13822937
BnA09	Na12-A01	66.8			
BnA09	BnGMS213B	68.2	BrA09_13917079_149	BrA09	13917079
BnA09	BnEMS973	70.9	BrA09_13642334_172	BrA09	13642334
BnA09	bna2905	72.6	BrA09_13278233_292	BrA09	13278233
BnA09	bna0275	72.9	BrA09_12962010_301	BrA09	12962010
BnA09	bna2891	72.9	BrA09_16845654_301	BrA09	16845654
BnA09	bna5100	73.2	BrA09_16640161_301	BrA09	16640161
BnA09	bna2895	73.2	BrA09_16640161_301	BrA09	16640161
BnA09	bna2894	73.2	BrA09_16690925_301	BrA09	16690925
BnA09	bna2893	73.2	BrA09_16722763_301	BrA09	16722763
BnA09	bna5469	73.5	BrA09_17036026_301	BrA09	17036026
BnA09	bna0245	73.8	BrA08_9367918_302		
BnA09	bna0244	73.8	BrA08_9420581_229		
BnA09	bna0248	73.8	BrA08_9276658_302		
BnA09	bna0246	73.8	BrA08_9326518_301		
BnA09	bna2887	73.8	BrA07_5758348_284		

BnA09	bna2313	74.1	BrA06_19739366_301		
BnA09	bna5662	74.1	BrA06_19739366_301		
BnA09	bna0328	74.1	BrA09_19111915_301	BrA09	19111915
BnA09	bna2882	74.1	BrA09_19145713_300	BrA09	19145713
BnA09	bna5417	74.1	BrA09_19152171_311	BrA09	19152171
BnA09	bna2872	74.1	BrA09_20115127_301	BrA09	20115127
BnA09	bna2180	74.4	BrA09_20620287_301	BrA09	20620287
BnA09	BGR60	75.3	BrA09_20556819_126	BrA09	20556819
BnA09	CB10022A	75.6			
BnA09	BGR42	75.6			
BnA09	sR9251J	76.2	BrA09_15477898_167	BrA09	15477898
BnA09	BEN278A	76.8	BoC09_8928998_155		
BnA09	BrGMS726B	78.8			
BnA09	BGR43	83.6	BrA09_23392125_137	BrA09	23392125
BnA09	BGR23	88	BrA09_24532607_124	BrA09	24532607
BnA09	BnGMS131	88.6	BrA09_24510113_135	BrA09	24510113
BnA09	BEN59	89.2	BrA09_23910646_178	BrA09	23910646
BnA09	BN9A	89.8			
BnA09	BnEMS1144	91.8	BrA09_23895488_281	BrA09	23895488
BnA09	bna2861	94.1	BrA09_23920888_299	BrA09	23920888
BnA09	bna2859	94.1	BrA09_23717141_301	BrA09	23717141
BnA09	bna2862	94.1	BrA09_23926596_301	BrA09	23926596
BnA09	bna2865	94.4	BrA09_24105625_301	BrA09	24105625
BnA09	bna5289	97.7	BrA05_15805956_301		
BnA09	bna4848	98	BrA09_24770365_301	BrA09	24770365
BnA09	bna1598	98.6	BrA09_25004211_301	BrA09	25004211
BnA09	bna1596	98.6	BrA09_25131434_304	BrA09	25131434
BnA09	bna2603	98.6	BrA09_25224371_302	BrA09	25224371
BnA09	bna2852	98.6	BrA09_25503885_301	BrA09	25503885
BnA09	sR12777	99.7	BrA09_24782574_242	BrA09	24782574
BnA09	BGO179A	100.6	BrA09_25571083_231	BrA09	25571083
BnA09	BoGMS788	100.9			
BnA09	bna2825	105.2	BrA09_28241830_308	BrA09	28241830
BnA09	bna5141	105.2	BrA09_28241830_308	BrA09	28241830
BnA09	bna4867	106.1	BrA09_28910557_301	BrA09	28910557
BnA09	bna2820	106.1	BrA09_28706021_301	BrA09	28706021
BnA09	bna2815	106.7	BrA09_29101489_302	BrA09	29101489
BnA09	bna5468	106.7	BrA09_29310612_301	BrA09	29310612
BnA09	bna2810	106.7	BrA09_29348737_293	BrA09	29348737
BnA09	bna2809	108.1	BrA09_29454769_300	BrA09	29454769
BnA09	bna5306	108.1	BrA09_29496278_301	BrA09	29496278
BnA09	bna4828	108.1	BrA03_22478492_271		
BnA09	BEN84	109.5	BrA09_29791688_229	BrA09	29791688
BnA09	bna2807	110.9	BrA09_29820627_301	BrA09	29820627
BnA09	Na14-B03	112.7			
BnA10	BnGMS249	0			
BnA10	BnGMS9	3.1	BrA10_1812746_129		

BnA10	BGR66	3.4	BrA10_135760_150	BrA10	135760
BnA10	bna3135	4.3	BrA10_284964_301	BrA10	284964
BnA10	bna0364	4.3	BrScaffold000123_221988_293		
BnA10	bna3106	4.3	BrA01_18702013_301		
BnA10	bna3129	4.3	BoC05_2322489_302		
BnA10	bna0205	4.3	BrScaffold000291_24321_301		
BnA10	bna5551	4.3	BrA02_22682772_310		
BnA10	bna1468	4.3	BrA02_22613210_254		
BnA10	bna4639	4.3	BrA02_22543533_301		
BnA10	bna3134	4.3	BrA10_379786_223	BrA10	379786
BnA10	bna5520	4.3	BrScaffold000123_29982_302		
BnA10	bna0262	4.3	BrA04_12452903_297		
BnA10	bna1469	4.3	BrA02_22693201_301		
BnA10	bna2899	4.3	BrScaffold000369_8642_301		
BnA10	bna0213	4.3	BrScaffold000123_306903_301		
BnA10	sR6083	5.2	BrA10_648054_414	BrA10	648054
BnA10	BEN210	5.2	BrA10_901957_108	BrA10	901957
BnA10	bna3127	6.1	BrA10_1513166_189	BrA10	1513166
BnA10	sORH13B	7	BrA10_1629861_259	BrA10	1629861
BnA10	BGR71A	7	BrA08_2185800_119		
BnA10	BoGMS1114	7			
BnA10	BGR64	7			
BnA10	bna4678	7.9	BrA10_3725683_301	BrA10	3725683
BnA10	bna3104	7.9	BrA10_4969953_301	BrA10	4969953
BnA10	bna3087	7.9	BrA10_7809191_301	BrA10	7809191
BnA10	bna5773	8.2	BoScaffold000034_P1_1356708_56		
BnA10	bna1815	8.5	BrA10_6180843_301	BrA10	6180843
BnA10	bna1814	8.5	BrA10_6199138_301	BrA10	6199138
BnA10	BoGMS197A	9.4	BrA10_6291918_156	BrA10	6291918
BnA10	bna2697	10.5	BrA10_6447037_301	BrA10	6447037
BnA10	bna3095	11.1	BrA10_7355531_301	BrA10	7355531
BnA10	bna3096	11.1	BoC09_28296469_301		
BnA10	bna5771	11.1	BoC09_28296469_301		
BnA10	bna3091	13.1	BrA10_7607081_299	BrA10	7607081
BnA10	bna3086	13.1	BrA10_7906431_301	BrA10	7906431
BnA10	bna5770	13.1	BrA10_7906431_301	BrA10	7906431
BnA10	bna4871	13.1	BrA10_8083775_303	BrA10	8083775
BnA10	bna4972	13.1	BrA10_8285199_301	BrA10	8285199
BnA10	bna0797	13.1	BoC05_4605240_301		
BnA10	bna3082	13.1	BrA10_8315472_301	BrA10	8315472
BnA10	bna5314	13.1	BrA10_8315472_301	BrA10	8315472
BnA10	BoGMS1199	14.5			
BnA10	BnEMS1185	15.1	BrA10_8369776_218	BrA10	8369776
BnA10	bna3079	16	BrA10_8387305_300	BrA10	8387305
BnA10	bna3081	16	BoC09_26000052_293		
BnA10	bna3074	16	BrA10_8564289_304	BrA10	8564289
BnA10	bna5936	16.6	BrA10_8766904_259	BrA10	8766904

BnA10	bna5704	16.6	BrScaffold000123_244680_301		
BnA10	bna5889	16.6	BrA10_8766904_259	BrA10	8766904
BnA10	bna3069	16.6	BrA10_8857702_301	BrA10	8857702
BnA10	bna1597	16.6	BrA09_25072577_299		
BnA10	bna5766	17.2	BrA10_9122134_302	BrA10	9122134
BnA10	bna5765	17.2	BrA10_9187027_301	BrA10	9187027
BnA10	bna3059	18.1	BrA10_9327235_301	BrA10	9327235
BnA10	bna5544	27.5	BrA09_31314801_290		
BnA10	bna5009	39.1	BrA10_13224157_301	BrA10	13224157
BnA10	bna5761	40.8	BrA10_13407917_302	BrA10	13407917
BnA10	bna5933	40.8	BrA10_13429404_301	BrA10	13429404
BnA10	BGO090	43.1	BrA10_13726214_212	BrA10	13726214
BnA10	BEN312	43.1	BoC09_33883158_221		
BnA10	bna4676	44	BoC09_33861997_259		
BnA10	bna5019	44	BoC09_33861997_259		
BnA10	bna3021	44	BrA10_13734397_303	BrA10	13734397
BnA10	bna3020	44	BrA10_13749676_299	BrA10	13749676
BnA10	bna5310	44.3	BrA10_13938715_302	BrA10	13938715
BnA10	bna3016	44.3	BrA10_13938715_302	BrA10	13938715
BnA10	bna3010	45.2	BrA10_14254838_301	BrA10	14254838
BnA10	bna5177	45.2	BrA10_14313425_301	BrA10	14313425
BnA10	bna3009	45.2	BrA10_14313425_301	BrA10	14313425
BnA10	bna5759	45.5	BrA10_14370230_311	BrA10	14370230
BnA10	bna3000	45.8	BrA10_14646372_302	BrA10	14646372
BnA10	bna2997	47.2	BrA10_14697676_302	BrA10	14697676
BnA10	bna5756	47.2	BrA10_14697676_302	BrA10	14697676
BnA10	bna2989	48.9	BrA10_15031960_301	BrA10	15031960
BnA10	bna2974	48.9	BoC09_37549777_301		
BnA10	bna2973	48.9	BrA10_16433391_205	BrA10	16433391
BnA10	bna5749	48.9	BrA10_16433391_205	BrA10	16433391
BnA10	BnEMS1173	54.5	BrA10_15360864_275	BrA10	15360864
BnA10	BnEMS1156	54.8			
BnA10	bna5471	56.2	BrA10_15331714_302	BrA10	15331714
BnA10	bna0874	56.8	BrA10_16503144_298	BrA10	16503144
BnA10	bna1805	59.4	BrA10_17449798_261	BrA10	17449798
BnA10	bna3242	59.4	BrA01_9626079_301		
BnA10	bna2968	59.4	BrA10_17501817_301	BrA10	17501817
BnA10	BnGMS175	60.3			
BnA10	BnEMS812	64.9			
BnC01	bna5649	0	BrA06_2902867_301	BrA06	2902867
BnC01	bna2234	1.7	BrA06_2756643_296	BrA06	2756643
BnC01	BoGMS1303	2.8			
BnC01	bna4787	3.4	BrA06_2542572_301	BrA06	2542572
BnC01	bna2228	3.7	BrA06_2261818_301	BrA06	2261818
BnC01	bna2222	4.6	BrA06_1613040_317	BrA06	1613040
BnC01	bna5645	4.6	BrA06_1613040_317	BrA06	1613040
BnC01	bna2220	4.6	BrA06_1529976_301	BrA06	1529976

BnC01	bna5093	4.6	BrA06_1480375_301	BrA06	1480375
BnC01	bna2219	4.6	BrA06_1480375_301	BrA06	1480375
BnC01	BEN391B	6.6			
BnC01	BGR16	7.5			
BnC01	BGO160	7.8			
BnC01	bna5716	8.7	BrA06_26268826_297		
BnC01	bna0385	8.7	BrA01_8207233_301		
BnC01	bna2770	8.7	BrA06_26268826_297		
BnC01	bna3211	23	BoC01_10693467_301	BoC01	10693467
BnC01	bna4695	23	BoC09_34391901_189		
BnC01	bna3210	23	BoC01_10738704_301	BoC01	10738704
BnC01	BEN6	24.7	BoC01_10755773_281	BoC01	10755773
BnC01	bna3207	26.7	BoC01_10912713_298	BoC01	10912713
BnC01	bna5825	26.7	BoC06_31921803_301		
BnC01	bna5025	26.7	BoScaffold000024_2832965_301		
BnC01	bna5826	26.7	BoC01_10912713_298	BoC01	10912713
BnC01	bna3206	26.7	BoC06_31921522_291		
BnC01	bna4878	26.7	BoScaffold000024_2832841_302		
BnC01	bna3203	26.7	BoC01_11024274_301	BoC01	11024274
BnC01	bna3198	27.8	BoC01_11204317_301	BoC01	11204317
BnC01	bna3197	28.1	BoC01_11212130_301	BoC01	11212130
BnC01	bna3196	28.1	BoC06_35205331_302		
BnC01	bna5060	28.1	BoC01_11218340_301	BoC01	11218340
BnC01	bna5483	28.1	BoC01_11261704_301	BoC01	11261704
BnC01	bna5024	28.1	BoC01_11284149_301	BoC01	11284149
BnC01	bna4694	28.1	BoC01_11284149_301	BoC01	11284149
BnC01	sN9425	28.7	BoC01_11302996_367	BoC01	11302996
BnC01	bna2009	36.5	BrA09_6090407_302		
BnC01	bna3249	36.5	BoC01_13821490_301	BoC01	13821490
BnC01	BEN348	37.1	BoC01_14211979_313	BoC01	14211979
BnC01	bna0969	38	BoScaffold000127_1253028_301		
BnC01	bna3760	39.1	BoScaffold000424_146746_302		
BnC01	bna0792	39.7	BoScaffold000127_609513_281		
BnC01	bna1032	39.7	BoC04_5903411_257		
BnC01	bna3243	41.7	BrA04_2359134_301		
BnC01	bna4696	41.7	BoC06_12911807_301		
BnC01	bna3247	41.7	BoScaffold000338_130703_302		
BnC01	bna3244	41.7	BoC05_9312267_188		
BnC01	BoGMS622	46.5			
BnC01	Na12-C08	50.2	BrA01_14848800_244		
BnC01	CB10258	51.6			
BnC01	sS1867A	51.9			
BnC01	BoGMS1246	52.2			
BnC01	bna3324	53.3	BoC01_14276456_301	BoC01	14276456
BnC01	bna3325	53.3	BoC01_14285456_301	BoC01	14285456
BnC01	bna5841	53.3	BoC01_14300995_301	BoC01	14300995
BnC01	bna3326	53.3	BoC01_14301103_301	BoC01	14301103

BnC01	bna3327	53.3	BoC01_14319520_264	BoC01	14319520
BnC01	bna3330	53.3	BoC01_14397348_301	BoC01	14397348
BnC01	bna3313	53.3	BoScaffold000361_152166_300		
BnC01	bna3319	53.3	BoScaffold000361_58170_301		
BnC01	bna3312	53.3	BoScaffold000361_162937_301		
BnC01	bna5437	53.3	BoC01_14403643_301	BoC01	14403643
BnC01	bna0891	53.3	BoC01_14522842_301	BoC01	14522842
BnC01	bna3338	53.3	BoC01_14848536_301	BoC01	14848536
BnC01	bna3336	53.3	BoC08_10783461_307		
BnC01	bna3316	53.3	BoScaffold000361_123005_301		
BnC01	bna0665	53.3	BoC01_16040336_301	BoC01	16040336
BnC01	bna3320	53.3	BoScaffold000361_48863_301		
BnC01	bna0532	53.3	BoScaffold000337_4413_301		
BnC01	bna3335	53.3	BoC01_16340725_301	BoC01	16340725
BnC01	bna3310	53.4	BoScaffold000361_218671_301		
BnC01	bna0701	53.5	BoScaffold000237_639353_294		
BnC01	bna3314	53.6	BoScaffold000361_140793_301		
BnC01	bna0832	53.7	BoC01_17141760_301	BoC01	17141760
BnC01	bna2979	54.3	BoC04_10825273_249		
BnC01	bna5239	56.3	BoC01_21703665_246	BoC01	21703665
BnC01	bna0574	57.5	BoC01_29077523_285	BoC01	29077523
BnC01	BoGMS1399	59.8			
BnC01	bna0941	60.9	BoC01_30797471_301	BoC01	30797471
BnC01	bna3342	60.9	BoC01_31074192_301	BoC01	31074192
BnC01	bna5879	60.9	BoC01_31086172_301	BoC01	31086172
BnC01	bna3343	60.9	BoC01_31149562_301	BoC01	31149562
BnC01	sN3523R	61.2	BoC01_31581658_199	BoC01	31581658
BnC01	bna0553	61.8	BoC01_31930694_301	BoC01	31930694
BnC01	bna0988	63.2	BoC03_24421151_301		
BnC01	bna3357	63.2	BoC03_50713788_301		
BnC01	bna3350	63.2	BoC01_32625067_301	BoC01	32625067
BnC01	BoGMS561	67.5	BoC01_36186383_144	BoC01	36186383
BnC01	BEN410	70.2	BoC01_37885135_241	BoC01	37885135
BnC01	BEN32B	70.6	BoC01_38372637_153	BoC01	38372637
BnC01	bna1286	71.4	BrA01_27902747_299		
BnC01	bna0878	71.4	BoC07_23218661_179		
BnC01	bna3369	71.4	BrA01_28250786_301		
BnC01	bna1298	71.4	BrA01_28260995_301		
BnC01	BRAS074A	74.8	BrA01_28424481_120		
BnC02	BrGMS231B	0			
BnC02	BGO054	1			
BnC02	bna5066	1.5	BrA07_20511170_301	BrA07	20511170
BnC02	bna2584	2	BrA07_21529099_298	BrA07	21529099
BnC02	bna5215	2	BrA07_21637773_301	BrA07	21637773
BnC02	bna2585	2	BoC07_943422_239		
BnC02	bna2588	2	BrA07_21918751_301	BrA07	21918751
BnC02	BrGMS231A	2.3			

BnC02	bna3407	17.6	BoC02_11048047_301		
BnC02	BEN425	25.7			
BnC02	MR52a	31.2	BoC02_6520290_134	BoC02	6520290
BnC02	BnEMS1119	34.5	BoC02_7359570_209	BoC02	7359570
BnC02	BrGMS268	34.5	BoC02_7359632_296	BoC02	7359632
BnC02	BRAS083	34.5	BoC02_7413861_156	BoC02	7413861
BnC02	BEN134	35.1	BoC02_8463131_197	BoC02	8463131
BnC02	bna0442	36.2	BoC02_8604252_305	BoC02	8604252
BnC02	bna1358	36.5	BrA02_5592528_299		
BnC02	bna5529	36.5	BoC02_5955002_301		
BnC02	bna1356	36.5	BrA02_5301560_301		
BnC02	bna1361	36.5	BrA02_5924413_148		
BnC02	bna5532	36.5	BrA02_6228686_200		
BnC02	bna2583	39.5	BrA07_21437273_301		
BnC02	BEN396	42.2			
BnC02	BoGMS328	42.8			
BnC02	sN3761A	43.4	BoC02_9227135_179	BoC02	9227135
BnC02	bna3385	44.3	BoScaffold000344_348629_302		
BnC02	bna3379	44.3	BoC06_26117133_301		
BnC02	bna3371	44.3	BoC06_25893091_286		
BnC02	bna3375	44.3	BoC06_26016799_299		
BnC02	bna3383	44.3	BrA10_7152347_268		
BnC02	bna3380	44.4	BoC06_26158817_188		
BnC02	bna3387	44.5	BoScaffold000024_78841_304		
BnC02	bna3389	44.6	BoScaffold000024_319346_301		
BnC02	bna3386	44.7	BoScaffold000024_11794_301		
BnC02	bna0551	44.7	BoC02_10311145_301	BoC02	10311145
BnC02	bna3413	44.7	BoC02_10734477_301	BoC02	10734477
BnC02	bna3409	44.7	BoC01_28896212_301		
BnC02	bna3410	44.7	BoC02_10914216_301	BoC02	10914216
BnC02	bna3392	44.7	BoC02_11087706_230	BoC02	11087706
BnC02	bna3384	44.7	BoScaffold000344_317376_304		
BnC02	bna4963	44.7	BoScaffold000024_58713_299		
BnC02	bna4700	44.7	BoC02_11100426_239	BoC02	11100426
BnC02	bna3522	45.5	BoScaffold000001_P2_243108_301		
BnC02	bna3525	45.5	BrA02_8653202_301		
BnC02	bna3524	45.5	BoScaffold000001_P2_214096_301		
BnC02	bna3520	45.5	BoScaffold000001_P2_334963_301		
BnC02	bna3519	45.6	BoScaffold000001_P2_358314_297		
BnC02	bna3517	45.7	BoScaffold000001_P2_387136_301		
BnC02	bna5942	47.4	BoScaffold000001_P2_669898_302		
BnC02	bna3505	48	BoScaffold000001_P2_1178512_301		
BnC02	bna4791	48	BoScaffold000001_P2_714220_301		
BnC02	bna3508	48	BoScaffold000001_P2_629586_302		
BnC02	bna3510	48	BoScaffold000001_P2_541721_301		
BnC02	Ol13-G05	49.4			
BnC02	BEN281A	51.3	BoC02_11649330_219	BoC02	11649330

BnC02	Na12-H09	52.5	BrA02_11624278_128		
BnC02	Ol09-A06A	53.5	BoC02_26800345_89		
BnC02	bna3862	55.7	BoC02_16785367_301	BoC02	16785367
BnC02	bna3863	55.7	BoC02_16849058_301	BoC02	16849058
BnC02	bna3864	55.7	BoC02_17022146_301	BoC02	17022146
BnC02	bna1423	56	BrA02_13167839_301		
BnC02	BGO146	80.6	BrA02_15287651_259	BrA02	15287651
BnC02	bna1223	81.8	BoC05_1773758_301		
BnC02	bna1439	82.7	BrA02_15879592_265	BrA02	15879592
BnC02	bna1440	82.7	BrA02_16588127_301	BrA02	16588127
BnC02	bna0309	82.8	BoC02_24685579_301		
BnC02	bna5051	82.9	BrA05_9241979_297		
BnC02	bna5545	82.9	BoC02_24759788_303		
BnC02	bna2134	83.2	BrA05_9166879_314		
BnC02	bna0619	83.2	BrA05_6407217_297		
BnC02	bna2135	83.5	BrA05_9080490_301		
BnC02	bna2136	83.5	BrA05_9054410_303		
BnC02	BGR58	84.6	BoC02_33628790_105		
BnC02	bna4836	84.9	BrA02_19401694_301	BrA02	19401694
BnC02	bna5035	84.9	BrA02_19401939_301	BrA02	19401939
BnC02	bna5209	84.9	BrA02_19573717_301	BrA02	19573717
BnC02	bna2268	84.9	BrA02_19573717_301	BrA02	19573717
BnC02	bna1455	84.9	BrA02_19646538_297	BrA02	19646538
BnC02	bna5627	85.2	BoC02_33293315_168		
BnC02	BEN296	87.5	BrA02_22857073_152	BrA02	22857073
BnC02	bna5548	88.1	BoC02_36430470_302		
BnC02	bna1472	89.2	BrA02_22157503_301	BrA02	22157503
BnC02	bna5552	89.2	BrA02_22157503_301	BrA02	22157503
BnC02	bna1473	89.2	BrA02_22357115_301	BrA02	22357115
BnC02	bna5554	90.1	BrA02_23362692_299	BrA02	23362692
BnC02	BRMS-026	93.1	BrA02_24682175_228	BrA02	24682175
BnC02	bna5556	95.1	BrA02_24201837_301	BrA02	24201837
BnC02	bna1490	95.4	BrA02_24269285_301	BrA02	24269285
BnC02	bna1494	95.4	BrA02_24580861_304	BrA02	24580861
BnC02	bna1088	96.3	BrA02_25898411_314	BrA02	25898411
BnC02	bna1087	96.6	BrA02_26014141_254	BrA02	26014141
BnC02	bna0664	97.2	BrA02_26034248_221	BrA02	26034248
BnC02	bna1086	97.2	BrA02_26034636_301	BrA02	26034636
BnC02	bna0053	97.2	BrA02_26164634_301	BrA02	26164634
BnC02	BEN14	98.1	BrA02_26265807_241	BrA02	26265807
BnC02	bna1080	100.1	BrA02_27246329_302	BrA02	27246329
BnC03	BnEMS824	0			
BnC03	BnEMS1171	0.9	BoC03_56984210_644	BoC03	56984210
BnC03	bna0488	2	BoC03_56831583_301	BoC03	56831583
BnC03	BGR89	4	BoC03_55727445_158	BoC03	55727445
BnC03	BoGMS1153	4.9			
BnC03	bna0578	38.2	BoC06_40640551_301		

BnC03	BoGMS576	40.5			
BnC03	Na12-E02B	42.4	BrA03_7953653_103		
BnC03	BoGMS693	44.9			
BnC03	BRAS120	44.9	BoC03_8598996_193	BoC03	8598996
BnC03	bna0584	49.5	BoC03_8917659_299	BoC03	8917659
BnC03	bna0109	54.1	BoC03_9403365_301	BoC03	9403365
BnC03	Ol10-E05	56.1	BoC03_9847453_95	BoC03	9847453
BnC03	bna3238	58.5	BoC03_10671466_301	BoC03	10671466
BnC03	bna3235	60.8	BoC03_10826083_300	BoC03	10826083
BnC03	bna3234	60.8	BoC03_10837267_301	BoC03	10837267
BnC03	bna5830	60.8	BoC03_10837868_301	BoC03	10837868
BnC03	bna3232	60.8	BoC03_10876501_299	BoC03	10876501
BnC03	bna0420	61.7	BoScaffold000417_72597_301		
BnC03	bna0815	62	BoC03_13119401_301	BoC03	13119401
BnC03	bna3642	62.6	BoC03_14248590_301	BoC03	14248590
BnC03	CB10057	63.7	BoC03_14249960_197	BoC03	14249960
BnC03	BRAS051A	63.7			
BnC03	sNRA56	63.7	BoC03_14457833_251	BoC03	14457833
BnC03	bna4752	64.8	BoC03_14715730_223	BoC03	14715730
BnC03	BRAS087A	65.9	BoC03_16290437_171	BoC03	16290437
BnC03	BRAS005	65.9	BoC03_16290458_196	BoC03	16290458
BnC03	bna3694	67	BoScaffold000445_93975_301		
BnC03	bna3641	67	BoC04_4765967_301		
BnC03	bna3695	67	BoScaffold000445_115904_301		
BnC03	bna1672	67	BrA03_12402499_301		
BnC03	bna3691	67	BoC03_16510429_301	BoC03	16510429
BnC03	bna3697	67.3	BoC03_17189721_301	BoC03	17189721
BnC03	bna3698	67.3	BoC03_17198073_301	BoC03	17198073
BnC03	bna0810	68.2	BoC03_17384693_301	BoC03	17384693
BnC03	bna0527	68.5	BoC03_18059121_301	BoC03	18059121
BnC03	BnGMS153	71.8	BoC03_19715716_184	BoC03	19715716
BnC03	bna3665	72.9	BoC03_19898731_291	BoC03	19898731
BnC03	FITO007.2	76.8			
BnC03	bna3725	79.5	BoScaffold000040_247857_301		
BnC03	bna4359	79.5	BoScaffold000040_345382_301		
BnC03	bna3728	79.8	BoScaffold000040_77369_301		
BnC03	bna3726	79.8	BoScaffold000040_222077_301		
BnC03	BrGMS556	85.1	BoC03_21360451_159	BoC03	21360451
BnC03	BEN78	90.4	BoC03_26376496_139	BoC03	26376496
BnC03	BnGMS584A	93.1	BoC03_27306726_219	BoC03	27306726
BnC03	bna0400	94	BoC03_27365297_301	BoC03	27365297
BnC03	Na10-E02C	96.8			
BnC03	Na10-E02B	100.3			
BnC03	Na12-G05	101.7			
BnC03	bna0842	102.6	BoC07_28315359_306		
BnC03	bna0989	102.6	BoC07_27998777_301		
BnC03	bna0711	102.6	BoScaffold000389_119417_304		

BnC03	bna0545	102.9	BoC03_29474486_301	BoC03	29474486
BnC03	bna0960	103.5	BoC03_29818084_301	BoC03	29818084
BnC04	bna4772	0	BoScaffold000479_44720_309		
BnC04	BEN337	2.3	BoC04_28531_179	BoC04	28531
BnC04	BnEMS634	2.3	BoC04_28573_164	BoC04	28573
BnC04	bna0741	2.6	BoC04_34927_301	BoC04	34927
BnC04	bna3881	2.6	BoScaffold000492_80177_301		
BnC04	bna3880	2.6	BoScaffold000492_36021_301		
BnC04	BRAS072	2.9			
BnC04	BEN266	2.9	BoC04_121523_205	BoC04	121523
BnC04	BGO140	7.8	BoC04_2985740_184	BoC04	2985740
BnC04	BoGMS876	8.1			
BnC04	sS2277	8.1	BoC04_2065086_215	BoC04	2065086
BnC04	bna5435	10.1	BoC03_21757372_301		
BnC04	bna3898	11.2	BoScaffold000152_P2_118199_301		
BnC04	bna3896	11.5	BoC04_7283093_289	BoC04	7283093
BnC04	bna3895	11.5	BoC04_7107888_301	BoC04	7107888
BnC04	bna3893	11.5	BoC04_7053518_301	BoC04	7053518
BnC04	bna3890	12.1	BoC04_6765723_301	BoC04	6765723
BnC04	bna3889	12.1	BoC04_6755490_300	BoC04	6755490
BnC04	bna3886	12.1	BoC04_6692801_301	BoC04	6692801
BnC04	bna3885	12.1	BoC04_6681467_301	BoC04	6681467
BnC04	bna3884	12.1	BoC04_6662671_301	BoC04	6662671
BnC04	BGR83	15.4	BoC04_4863957_119	BoC04	4863957
BnC04	bna3901	16.3	BoC04_4853652_301	BoC04	4853652
BnC04	bna3902	16.3	BoC04_4832311_301	BoC04	4832311
BnC04	bna3903	16.3	BoC04_4811011_301	BoC04	4811011
BnC04	bna3904	16.3	BoC04_4788675_301	BoC04	4788675
BnC04	bna3905	16.3	BoC04_4687761_301	BoC04	4687761
BnC04	bna3910	16.3	BoC04_4503191_230	BoC04	4503191
BnC04	bna2154	19.9	BrA05_5043649_301		
BnC04	bna5967	23.2	BoC04_20851795_301	BoC04	20851795
BnC04	BGO141	24.6	BoC04_20034307_263	BoC04	20034307
BnC04	BoGMS560	25.2	BoC04_34176660_276		
BnC04	bna0695	30.8	BoScaffold000024_972317_297		
BnC04	BEN269	35.7	BoC04_19590343_175		
BnC04	bna3769	36.6	BoScaffold000498_71526_216		
BnC04	bna4000	47.7	BoC01_25489245_300		
BnC04	bna4001	48	BoC01_25461049_301		
BnC04	bna4718	48	BoC04_29837685_301	BoC04	29837685
BnC04	BEN239	50	BoC04_30860617_210	BoC04	30860617
BnC04	BGO041	50	BoC04_30860620_207	BoC04	30860620
BnC04	bna4135	50.3	BoC04_31011812_301	BoC04	31011812
BnC04	CB10196	50.9	BoC04_33057690_247	BoC04	33057690
BnC04	BEN30A	52.3	BoC04_36133841_239	BoC04	36133841
BnC04	bna4009	53.4	BoC03_20863405_301		
BnC04	bna4019	53.7	BoC04_36163611_301	BoC04	36163611

BnC04	bna4022	54.6	BoC04_36389682_301	BoC04	36389682
BnC04	bna4032	56	BoC04_37567682_301	BoC04	37567682
BnC04	bna4033	56.7	BoC04_37601346_301	BoC04	37601346
BnC04	bna4038	57.4	BoC04_37790278_301	BoC04	37790278
BnC04	bna5613	57.4	BoC04_37966586_296	BoC04	37966586
BnC04	BEN260	57.7	BoC04_38004702_204	BoC04	38004702
BnC04	bna4042	58	BoC04_38020170_301	BoC04	38020170
BnC04	bna5200	58	BrA04_17055673_303		
BnC04	bna2011	58	BrA04_17309194_302		
BnC04	bna4044	58	BoC04_38084313_301	BoC04	38084313
BnC04	bna4046	58	BoC04_38310490_301	BoC04	38310490
BnC04	BEN28	58.3	BoC04_38625818_244	BoC04	38625818
BnC04	BEN420	58.3			
BnC04	BEN330A	60.9	BoC04_39009491_117	BoC04	39009491
BnC04	bna2013	63.9	BoC04_39112360_301	BoC04	39112360
BnC04	Na12-E05A	64.2	BoC04_39335346_159	BoC04	39335346
BnC04	bna3657	64.5	BoC08_15576714_301		
BnC04	bna5088	64.5	BrA04_18941625_301		
BnC04	bna3659	64.5	BoC04_39408421_301	BoC04	39408421
BnC04	bna5901	65.4	BoScaffold000383_71931_301		
BnC04	bna3654	71.4	BoC04_39447484_301	BoC04	39447484
BnC04	Ol10-B01C	77.4	BoC04_39600479_169	BoC04	39600479
BnC04	bna4048	77.7	BoC04_39634651_301	BoC04	39634651
BnC04	bna4050	77.7	BoC04_39746095_301	BoC04	39746095
BnC04	BEN387	79.7	BoC04_39865802_148	BoC04	39865802
BnC04	bna4051	81.7	BoC04_39944696_258	BoC04	39944696
BnC04	bna4054	81.7	BoC04_40220984_301	BoC04	40220984
BnC04	BGO044	82	BoC04_40264415_217	BoC04	40264415
BnC04	BEN376	85	BoC04_40441062_146	BoC04	40441062
BnC04	BEN216	88.3	BoC04_40450483_103	BoC04	40450483
BnC04	BnEMS439B	89.7	BrA04_18048955_207		
BnC04	BEN313A	91.4	BoC04_40800903_129	BoC04	40800903
BnC04	Na12-E05B	104.7	BoC04_39335346_159		
BnC04	Ol10-B01A	105.1	BoC04_39600479_169		
BnC04	bna1518	109.9	BrA04_18370211_301	BrA04	18370211
BnC04	bna1519	109.9	BrA04_18248518_214	BrA04	18248518
BnC04	bna1522	110.8	BrA04_18097253_288	BrA04	18097253
BnC04	Ol10-B01B	110.8	BoC04_39600479_169		
BnC04	bna5246	111.1	BrA04_17577550_301	BrA04	17577550
BnC04	BrGMS426	119	BoC04_37779242_205		
BnC04	BEN30B	124.7	BoC04_36133841_239		
BnC04	bna4017	125.3	BoC04_35968371_301		
BnC04	bna1987	125.3	BrA04_15411549_301	BrA04	15411549
BnC04	bna1816	125.6	BrA04_14899563_301	BrA04	14899563
BnC04	bna1975	125.6	BrA04_14885528_301	BrA04	14885528
BnC04	bna4013	125.6	BoC04_35723468_301		
BnC04	bna1973	125.6	BrA04_14639857_301	BrA04	14639857

BnC04	bna5286	125.6	BrA04_14588034_301	BrA04	14588034
BnC04	bna1965	125.9	BrA04_13571365_291	BrA04	13571365
BnC04	bna1964	125.9	BrA04_13541330_301	BrA04	13541330
BnC04	bna5383	125.9	BrA04_13490597_299	BrA04	13490597
BnC04	bna1963	125.9	BrA04_13435626_298	BrA04	13435626
BnC04	bna1983	125.9	BoC04_32509105_285		
BnC04	bna1984	125.9	BoC04_32547519_302		
BnC04	bna1985	125.9	BrA04_13293395_301	BrA04	13293395
BnC04	BGR55	126.8	BrA04_13660464_142	BrA04	13660464
BnC04	Ol11-B05	129.8			
BnC04	bna1956	134.7	BrA04_12982228_301	BrA04	12982228
BnC04	bna1958	135.3	BrA04_12775106_301	BrA04	12775106
BnC04	bna5039	135.3	BrA04_12759591_267	BrA04	12759591
BnC04	BnEMS843	138.3	BoC04_30578742_303		
BnC04	bna1930	138.9	BrA04_12051217_211	BrA04	12051217
BnC04	bna1929	139.2	BrA04_11857893_301	BrA04	11857893
BnC04	bna5601	139.5	BrA04_11641375_301	BrA04	11641375
BnC04	bna1927	140.1	BrA04_11512229_301	BrA04	11512229
BnC04	bna1925	140.1	BrA04_11436981_301	BrA04	11436981
BnC04	bna5959	140.7	BoScaffold000008_P1_232056_301		
BnC04	bna4796	140.7	BoScaffold000008_P1_233001_301		
BnC04	bna5542	140.7	BrA03_24870135_301		
BnC04	bna1433	140.7	BrA03_24869598_301		
BnC04	bna1923	140.7	BrA04_10941413_232	BrA04	10941413
BnC04	BGO078	141.6	BrA04_10745608_134	BrA04	10745608
BnC04	sN2025	143	BrA04_10382393_654	BrA04	10382393
BnC04	bna1913	147.3	BrA04_9930221_301	BrA04	9930221
BnC04	bna5598	147.3	BrA04_9866051_301	BrA04	9866051
BnC04	bna1912	147.3	BrA04_9866051_301	BrA04	9866051
BnC04	bna0161	147.3	BoScaffold000053_1407745_301		
BnC04	bna0128	147.3	BrA04_9829689_301	BrA04	9829689
BnC04	bna5199	147.9	BrA04_9543819_301	BrA04	9543819
BnC04	bna1910	147.9	BrA04_9531877_301	BrA04	9531877
BnC04	bna1906	148.8	BoScaffold000301_484897_216		
BnC04	bna1905	148.8	BrA04_8788332_264	BrA04	8788332
BnC04	bna1903	149.9	BrA04_8431313_305	BrA04	8431313
BnC04	BGR71B	150.5			
BnC04	BEN95	151.6	BrA04_8694754_115	BrA04	8694754
BnC04	BEN229	152.5	BoC04_29308814_232		
BnC04	bna0006	153.6	BrA04_7342568_290	BrA04	7342568
BnC05	BGR49	0	BoC05_27137863_144		
BnC05	bna3300	1.1	BoScaffold000237_761486_300		
BnC05	bna0559	1.1	BoC05_26882248_301		
BnC05	bna4794	4.7	BoC04_13352367_301		
BnC05	bna0448	6.1	BoScaffold000450_12080_301		
BnC05	BnGMS208	7			
BnC05	BnGMS198	7			

BnC05	sS2131	7.9	BoC05_8854465_174		
BnC05	BoGMS319	16.8	BoC05_7007360_227	BoC05	7007360
BnC05	bna4117	19.8	BoC05_13932622_288	BoC05	13932622
BnC05	bna4125	19.8	BoC05_29708580_318		
BnC05	bna4124	19.8	BoC05_15224000_302	BoC05	15224000
BnC05	bna4127	19.8	BoC05_15339888_301	BoC05	15339888
BnC05	bna4289	21.5	BoC05_16473116_245	BoC05	16473116
BnC05	bna4288	21.5	BoC05_16496431_301	BoC05	16496431
BnC05	bna0194	21.8	BoC09_19095369_301		
BnC05	Na12-C01	22.7	BoC05_16975819_85	BoC05	16975819
BnC05	bna4065	26.6	BoScaffold000333_229916_301		
BnC05	bna4072	26.9	BoC05_25517337_305	BoC05	25517337
BnC05	bna4073	27.2	BoC05_25556828_227	BoC05	25556828
BnC05	bna4075	27.5	BoC05_25710318_302	BoC05	25710318
BnC05	bna4076	28.1	BoC05_25894318_301	BoC05	25894318
BnC05	bna3414	28.1	BoC02_10603202_301		
BnC05	BEN81	29	BoC05_25942418_171	BoC05	25942418
BnC05	bna4078	29.9	BoC05_25948119_305	BoC05	25948119
BnC05	bna4079	29.9	BoC05_26000822_301	BoC05	26000822
BnC05	bna4080	30.2	BoC05_26021073_301	BoC05	26021073
BnC05	bna4082	33.5	BoC05_3205578_301	BoC05	3205578
BnC05	bna4083	34.6	BoC05_3151125_301	BoC05	3151125
BnC05	BEN363	35.5	BoC05_3150464_236	BoC05	3150464
BnC05	BGO151	35.5	BoC05_3149393_257	BoC05	3149393
BnC05	bna4084	37.8	BoC05_2997517_301	BoC05	2997517
BnC05	bna4085	38.4	BoScaffold000087_P1_63592_301		
BnC05	bna4089	41.7	BoC05_2576267_301	BoC05	2576267
BnC05	bna4090	41.7	BoC05_2553972_301	BoC05	2553972
BnC05	bna4093	42.3	BoC05_2440345_300	BoC05	2440345
BnC05	bna4094	42.3	BoC05_2416377_281	BoC05	2416377
BnC05	sORH13A	43.7			
BnC05	bna4056	49.7	BoC05_395352_301	BoC05	395352
BnC05	bna4057	49.7	BoC05_283542_301	BoC05	283542
BnC05	BEN140	81			
BnC05	bna0623	81.9	BoC09_9601331_299		
BnC05	BoGMS1561	82.5			
BnC05	FITO114	86.4			
BnC05	bna3301	86.7	BoScaffold000249_566822_301		
BnC05	bna4610	90	BoScaffold000364_152113_301		
BnC05	BEN285	111.1	BrA05_2348320_166		
BnC05	BrGMS387	113.5	BrA05_1577399_319	BrA05	1577399
BnC05	bna2197	114.6	BrA05_1492477_301	BrA05	1492477
BnC05	bna2192	115.5	BrA05_2109698_321	BrA05	2109698
BnC05	bna2187	116.1	BrA05_2287965_301	BrA05	2287965
BnC05	bna5640	116.1	BrA05_2402280_301	BrA05	2402280
BnC05	bna0023	116.7	BrA05_2534365_297	BrA05	2534365
BnC05	bna2182	116.7	BrA05_2604626_246	BrA05	2604626

BnC05	bna2181	117.3	BrA05_2718163_301	BrA05	2718163
BnC05	bna0371	117.3	BrA05_2752010_301	BrA05	2752010
BnC05	bna2179	119.6	BrA05_3043046_300	BrA05	3043046
BnC05	bna2178	120.5	BrA05_3135216_162	BrA05	3135216
BnC05	bna5639	120.8	BrA05_3324028_301	BrA05	3324028
BnC05	bna2174	120.8	BrA05_3550306_265	BrA05	3550306
BnC05	bna2173	120.8	BrA05_3610261_301	BrA05	3610261
BnC05	bna5453	120.8	BrA05_3615531_301	BrA05	3615531
BnC05	bna5205	121.1	BrA05_3782985_299	BrA05	3782985
BnC05	BEN211	122.8	BrA05_5098813_154	BrA05	5098813
BnC05	BRAS063	124.2	BrA05_3298798_243		
BnC05	BnEMS1036	124.2	BrA05_3202272_265		
BnC05	BrGMS351	124.5	BrA05_3298816_227		
BnC05	BGR88B	132.7	BrA05_18227120_147		
BnC05	BGR51	133.8	BrA05_16460698_150		
BnC05	BrGMS124	133.8	BoC05_19788361_158		
BnC05	Na10-E02A	134.1	BrA06_16875254_125		
BnC05	BnEMS1072	134.2	BrA06_20995022_200		
BnC05	Na12-E01	134.3			
BnC05	bna2138	135.4	BrA05_7855910_301	BrA05	7855910
BnC05	bna2120	135.4	BrA05_9646616_251	BrA05	9646616
BnC05	bna5629	135.4	BrA05_9900847_301	BrA05	9900847
BnC05	bna2118	135.7	BrA05_10292928_301	BrA05	10292928
BnC05	bna2124	135.7	BrA05_11106780_301	BrA05	11106780
BnC05	bna2128	135.7	BrA05_11607194_301	BrA05	11607194
BnC05	bna2069	136	BoC05_8041058_241		
BnC05	bna1165	136.3	BoC09_24054236_321		
BnC05	bna5130	136.3	BrA05_11714048_301	BrA05	11714048
BnC05	bna2131	136.3	BrA05_11718716_303	BrA05	11718716
BnC05	bna2133	136.3	BrA05_11840091_301	BrA05	11840091
BnC05	bna2116	136.6	BrA05_11945153_301	BrA05	11945153
BnC05	bna2115	136.6	BrA05_11980424_301	BrA05	11980424
BnC05	bna2114	136.6	BrA05_11984544_301	BrA05	11984544
BnC05	bna2113	136.6	BrA05_12011855_301	BrA05	12011855
BnC05	bna2122	137.2	BrA01_11993737_299		
BnC05	bna5870	137.8	BrA05_12033088_301	BrA05	12033088
BnC05	bna2111	137.8	BrA05_12052721_301	BrA05	12052721
BnC05	bna2107	137.8	BrA05_12307753_301	BrA05	12307753
BnC05	bna0668	137.8	BoC03_9427817_301		
BnC05	bna2105	137.8	BrA05_12426734_301	BrA05	12426734
BnC05	bna2104	137.8	BrA05_12457820_301	BrA05	12457820
BnC05	bna0354	137.8	BrA05_12838818_301	BrA05	12838818
BnC05	bna1120	137.8	BrScaffold000280_11257_302		
BnC05	bna2061	137.8	BrA05_13227028_301	BrA05	13227028
BnC05	bna0342	137.8	BrA05_13301566_206	BrA05	13301566
BnC05	bna2751	138.1	BrA08_1336839_301		
BnC05	bna5620	138.4	BrA05_13538777_301	BrA05	13538777

BnC05	bna2071	138.4	BrA05_13880226_296	BrA05	13880226
BnC05	bna0253	138.4	BrScaffold000201_41824_301		
BnC05	bna0252	138.4	BrA02_22725096_301		
BnC05	bna2075	138.4	BrA05_14029064_301	BrA05	14029064
BnC05	bna2060	138.4	BrScaffold004022_427_301		
BnC05	BGR50	139.3	BoC05_13100763_143		
BnC05	bna2076	140.2	BrA05_14040757_301	BrA05	14040757
BnC05	bna2077	140.2	BrA05_14155289_301	BrA05	14155289
BnC05	bna2082	140.2	BrA05_14435311_301	BrA05	14435311
BnC05	bna2083	140.2	BrA05_14444029_301	BrA05	14444029
BnC05	bna2085	140.2	BrA05_14595356_301	BrA05	14595356
BnC05	bna5012	140.2	BrA05_14740842_277	BrA05	14740842
BnC05	bna2087	140.2	BrA05_14742565_301	BrA05	14742565
BnC05	bna2091	140.2	BrA05_15013435_301	BrA05	15013435
BnC05	bna5625	140.2	BrA05_15086181_301	BrA05	15086181
BnC05	bna2066	140.2	BrA05_15356777_304	BrA05	15356777
BnC05	bna2057	140.5	BrA05_16835720_305	BrA05	16835720
BnC05	bna2056	140.5	BrA05_16844353_301	BrA05	16844353
BnC05	bna4801	141.1	BrA05_17757971_229	BrA05	17757971
BnC05	bna2375	141.1	BrA05_17832691_301	BrA05	17832691
BnC05	bna5017	141.1	BrA05_17913610_301	BrA05	17913610
BnC05	bna5619	141.4	BrA05_18458288_258	BrA05	18458288
BnC05	bna4729	141.4	BoScaffold000509_10901_301		
BnC05	bna2049	141.7	BrA05_18654178_301	BrA05	18654178
BnC05	bna4798	141.7	BoC05_23892687_300		
BnC05	bna0769	141.7	BoC05_23817122_301		
BnC05	bna2048	141.7	BrA05_18778891_301	BrA05	18778891
BnC05	bna1162	142.3	BoScaffold000106_777496_318		
BnC05	bna2045	144	BrA05_21047756_279	BrA05	21047756
BnC05	bna5617	144.6	BrA05_21102199_301	BrA05	21102199
BnC05	bna2044	144.6	BrA05_21107331_301	BrA05	21107331
BnC05	bna2043	144.6	BrA05_21481144_301	BrA05	21481144
BnC05	bna2042	144.9	BrA05_21631356_301	BrA05	21631356
BnC05	bna5011	145.2	BrA05_21646834_301	BrA05	21646834
BnC05	BGR70	145.2	BrA05_21844706_150	BrA05	21844706
BnC06	BEN374B	0	BoC07_1042174_213		
BnC06	bna5689	9	BrA07_21961835_303	BrA07	21961835
BnC06	bna2589	9	BrA07_22034312_301	BrA07	22034312
BnC06	bna2592	9	BrA07_22193291_301	BrA07	22193291
BnC06	bna2593	9	BoC07_302532_301	BoC07	302532
BnC06	BEN335	9	BoC07_30944062_119		
BnC06	bna2587	9.3	BoC07_771518_295	BoC07	771518
BnC06	BEN374A	11.3	BoC07_1042174_213	BoC07	1042174
BnC06	BnEMS994	13.3	BoC07_1198475_240	BoC07	1198475
BnC06	bna2577	15	BrA07_20501932_294		
BnC06	bna1012	15	BoC07_2451947_301	BoC07	2451947
BnC06	bna2570	15	BrA07_19430864_301		

BnC06	bna5863	15	BoC01_10128342_270		
BnC06	bna5410	15	BoC01_25355578_299		
BnC06	bna2747	15	BoC01_25355779_301		
BnC06	bna0395	15	BrA07_21174257_235		
BnC06	bna5135	15.3	BrA07_18992968_301		
BnC06	BEN228	18.6	BoC07_2509216_254	BoC07	2509216
BnC06	bna3900	18.6	BoC04_4881367_301		
BnC06	BoGMS1186	18.6			
BnC06	bna4754	18.6	BoC07_2693719_260	BoC07	2693719
BnC06	bna5046	18.6	BrA07_17488642_228		
BnC06	BnEMS82B	22.5	BoC07_2965046_191	BoC07	2965046
BnC06	bna4145	23.4	BoC07_3320152_301	BoC07	3320152
BnC06	bna4144	23.4	BoC07_3469180_301	BoC07	3469180
BnC06	BnEMS1124	23.7	BoC07_3642978_334	BoC07	3642978
BnC06	bna4116	24	BoC07_4899883_227	BoC07	4899883
BnC06	BEN16	24	BoC07_4905589_121	BoC07	4905589
BnC06	bna4115	24.3	BoC07_4914531_301	BoC07	4914531
BnC06	BGO055	24.6	BoC07_7490142_210	BoC07	7490142
BnC06	bna4793	24.9	BoC07_7504465_225	BoC07	7504465
BnC06	BEN271	24.9	BoC07_7556363_123	BoC07	7556363
BnC06	BGO056	25.8	BoC07_7556363_123	BoC07	7556363
BnC06	bna4106	26.7	BoC07_7558675_301	BoC07	7558675
BnC06	bna4901	26.7	BoC07_7711919_297	BoC07	7711919
BnC06	BEN327	26.7	BoC07_8457556_255	BoC07	8457556
BnC06	BEN341A	27	BoC07_8535142_132	BoC07	8535142
BnC06	Ap1c 5pr	27.3			
BnC06	BEN341B	30.8	BoC07_8535142_132	BoC07	8535142
BnC06	bna0669	31.9	BoC07_8913646_301	BoC07	8913646
BnC06	bna2558	32.8	BrA07_17705637_301	BrA07	17705637
BnC06	bna2553	32.8	BrA07_17388386_301	BrA07	17388386
BnC06	bna0114	34.8	BrA07_16767833_301	BrA07	16767833
BnC06	bna0175	34.8	BrA07_16759938_301	BrA07	16759938
BnC06	bna4782	34.8	BrA07_16758082_301	BrA07	16758082
BnC06	bna0113	34.8	BrA07_16757315_301	BrA07	16757315
BnC06	bna0046	34.8	BrA07_16755651_301	BrA07	16755651
BnC06	bna0174	34.9	BrA07_16728857_301	BrA07	16728857
BnC06	bna4952	35	BrA07_16722238_285	BrA07	16722238
BnC06	bna0017	35.1	BrA07_16701988_301	BrA07	16701988
BnC06	bna0034	35.2	BrA07_16695280_301	BrA07	16695280
BnC06	bna2545	35.3	BrA07_16686772_302	BrA07	16686772
BnC06	bna2544	35.4	BrA07_16677737_301	BrA07	16677737
BnC06	bna0133	35.7	BrA07_16558893_301	BrA07	16558893
BnC06	bna4781	36	BrA07_16100952_301	BrA07	16100952
BnC06	bna0033	36	BrA07_16100357_301	BrA07	16100357
BnC06	bna4780	36	BrA07_16099540_301	BrA07	16099540
BnC06	bna0016	36	BrA07_16095439_301	BrA07	16095439
BnC06	bna2539	36	BrA07_16095439_301	BrA07	16095439

BnC06	bna0171	36	BrA07_16089881_301	BrA07	16089881
BnC06	bna0068	36	BrA07_16070666_301	BrA07	16070666
BnC06	bna0015	36	BrA07_16040715_301	BrA07	16040715
BnC06	bna2538	36	BrA07_15931600_301	BrA07	15931600
BnC06	bna2528	36.3	BrA07_15058904_301	BrA07	15058904
BnC06	FITO035A	36.3			
BnC06	bna0470	36.3	BoC07_9423305_301		
BnC06	bna2525	36.3	BrA07_14970124_301	BrA07	14970124
BnC06	BnEMS82A	36.9	BoC07_32508597_207		
BnC06	BnGMS205	38.8			
BnC06	bna0148	40.4	BoC07_35677613_301	BoC07	35677613
BnC06	bna4278	40.4	BoScaffold000356_98841_301		
BnC06	bna1006	40.4	BoC06_33759140_301		
BnC06	bna0009	40.4	BoC07_35675639_301	BoC07	35675639
BnC06	Ol12-E03B	41.5	BoC07_35576050_117	BoC07	35576050
BnC06	BEN185	42.6			
BnC06	bna4285	42.6	BoC07_35504618_268	BoC07	35504618
BnC06	bna1052	42.6	BoC07_34184488_301	BoC07	34184488
BnC06	bna0650	42.6	BoC07_33598518_273	BoC07	33598518
BnC06	bna2529	42.6	BoC07_32751398_304	BoC07	32751398
BnC06	FITO095	42.6			
BnC06	BGO156A	42.6	BoC07_32557010_172	BoC07	32557010
BnC06	bna4628	42.6	BoC04_10754934_301		
BnC06	BGR99B	48.3	BoC07_31854860_133	BoC07	31854860
BnC06	bna0867	58.6	BoC07_29771104_301	BoC07	29771104
BnC06	Ol10-F09	60	BoC07_28931103_119	BoC07	28931103
BnC06	bna0892	60.3	BoC05_7077119_301		
BnC06	bna4154	61.2	BoC07_27663436_303	BoC07	27663436
BnC06	bna4157	61.2	BoC07_27513541_301	BoC07	27513541
BnC06	bna0656	63.2	BoC07_16839511_301		
BnC06	BnGMS147B	63.2	BrA07_13588712_232		
BnC06	bna4965	64.1	BoC07_16180589_301		
BnC07	BoGMS1032	0			
BnC07	bna4251	2.7	BoC06_2074938_285	BoC06	2074938
BnC07	bna4726	2.7	BoC06_2100997_301	BoC06	2100997
BnC07	bna4252	2.7	BoC06_2114909_301	BoC06	2114909
BnC07	bna4254	2.7	BoC06_2200019_301	BoC06	2200019
BnC07	bna4255	2.7	BoC06_2247440_301	BoC06	2247440
BnC07	bna4906	2.7	BoC06_2275925_301	BoC06	2275925
BnC07	bna4258	2.7	BoC06_2366458_283	BoC06	2366458
BnC07	bna4261	2.7	BoC06_2471432_305	BoC06	2471432
BnC07	bna4263	2.7	BoC06_2490783_301	BoC06	2490783
BnC07	Na12-F03	3	BoC06_2511274_275	BoC06	2511274
BnC07	bna4266	3.3	BoC06_2778576_301	BoC06	2778576
BnC07	CB10297	3.9	BoC06_2953737_265	BoC06	2953737
BnC07	sNRH63	4.2			
BnC07	BRAS019	4.2			

BnC07	bna0935	4.5	BoC06_3403068_301	BoC06	3403068
BnC07	BN25C2	5.4	BoC06_4577757_142	BoC06	4577757
BnC07	bna0901	5.7	BoC06_4584719_301	BoC06	4584719
BnC07	Na10-C01B	6	BoC06_5650953_186	BoC06	5650953
BnC07	CB10217	6	BoC06_5914112_163	BoC06	5914112
BnC07	bna4195	6.9	BoScaffold000410_40826_301		
BnC07	bna3967	6.9	BoC06_9908054_301	BoC06	9908054
BnC07	bna4293	6.9	BoC06_12451594_301	BoC06	12451594
BnC07	bna4194	6.9	BoScaffold000410_19634_301		
BnC07	bna3768	6.9	BoC07_24416978_301		
BnC07	bna0459	6.9	BoC06_12726319_301	BoC06	12726319
BnC07	bna4189	7.2	BoC06_13553142_301	BoC06	13553142
BnC07	bna4188	7.2	BoC06_13579405_301	BoC06	13579405
BnC07	bna4186	7.2	BoC06_13624868_301	BoC06	13624868
BnC07	bna4903	7.2	BoC06_13687812_193	BoC06	13687812
BnC07	bna4184	7.2	BoC06_13765187_302	BoC06	13765187
BnC07	bna4182	7.2	BoC02_25633759_301		
BnC07	bna4183	7.2	BoC06_13809881_301	BoC06	13809881
BnC07	bna4181	7.2	BoC06_13872424_301	BoC06	13872424
BnC07	bna4180	7.2	BoC06_13886682_301	BoC06	13886682
BnC07	bna4179	7.2	BoC06_13994462_301	BoC06	13994462
BnC07	bna4177	7.2	BoC06_14027102_301	BoC06	14027102
BnC07	bna4176	7.2	BoC06_14046580_301	BoC06	14046580
BnC07	bna4174	7.2	BoC06_14122045_301	BoC06	14122045
BnC07	bna4172	7.2	BoC06_14197030_301	BoC06	14197030
BnC07	bna4169	7.2	BoC06_14278577_301	BoC06	14278577
BnC07	bna4902	7.2	BoC06_14314918_301	BoC06	14314918
BnC07	bna4167	7.2	BoC06_14324578_301	BoC06	14324578
BnC07	bna4165	7.2	BoC06_14627525_301	BoC06	14627525
BnC07	bna4191	7.2	BoScaffold000960_1800_301		
BnC07	bna4164	7.2	BoC06_14723271_301	BoC06	14723271
BnC07	bna4162	7.5	BoC06_14770118_301	BoC06	14770118
BnC07	bna4161	7.5	BoC06_14804908_300	BoC06	14804908
BnC07	bna4226	7.5	BoC06_14828402_301	BoC06	14828402
BnC07	bna4724	7.5	BrA10_3001069_301		
BnC07	bna4227	7.5	BoC06_14860771_301	BoC06	14860771
BnC07	bna4228	7.5	BoC06_14888151_301	BoC06	14888151
BnC07	bna4229	7.5	BoC06_14926384_301	BoC06	14926384
BnC07	bna0721	7.5	BoC03_39349623_301		
BnC07	bna4232	7.5	BoC06_15015127_301	BoC06	15015127
BnC07	bna4234	7.5	BoC06_15054584_301	BoC06	15054584
BnC07	bna4235	7.5	BoC06_15072864_301	BoC06	15072864
BnC07	bna4236	7.5	BoC06_15105978_301	BoC06	15105978
BnC07	bna4808	7.5	BoC06_15175329_301	BoC06	15175329
BnC07	sN0706	7.8	BoC06_15240254_357	BoC06	15240254
BnC07	bna4907	8.1	BrScaffold000239_42138_304		
BnC07	bna4238	8.1	BoC06_15360330_301	BoC06	15360330

BnC07	bna4240	8.1	BoC06_15620608_301	BoC06	15620608
BnC07	bna4242	8.1	BoC06_15665403_301	BoC06	15665403
BnC07	bna4245	8.1	BoC06_15739861_301	BoC06	15739861
BnC07	bna4246	8.1	BoC06_15748825_301	BoC06	15748825
BnC07	bna4725	8.1	BoC06_15756688_271	BoC06	15756688
BnC07	bna4248	8.1	BoC06_15878853_301	BoC06	15878853
BnC07	bna4247	8.1	BoC06_15801680_301	BoC06	15801680
BnC07	bna4262	8.1			
BnC07	bna1003	8.7	BoC08_23087748_239	BoC08	23087748
BnC07	bna4196	8.7	BoC08_23737763_301	BoC08	23737763
BnC07	bna4198	9.3	BoC08_25975058_301	BoC08	25975058
BnC07	bna4199	9.3	BoC08_26108471_301	BoC08	26108471
BnC07	bna4200	9.3	BoC08_26146753_247	BoC08	26146753
BnC07	bna4201	9.3	BoScaffold000719_1655_301		
BnC07	bna4202	9.3	BoC08_26204631_301	BoC08	26204631
BnC07	bna4203	9.3	BoC08_26244524_301	BoC08	26244524
BnC07	bna0731	15.6	BoC06_24097329_299	BoC06	24097329
BnC07	bna1041	19.5	BoC06_28284306_301	BoC06	28284306
BnC07	bna0474	20.9	BoC09_25920840_231		
BnC07	bna1055	23.2	BoC06_31734439_276	BoC06	31734439
BnC07	sR12156B	23.8	BoC06_31818111_169	BoC06	31818111
BnC07	bna4304	25.2	BoC06_32533026_301	BoC06	32533026
BnC07	bna4908	25.2	BoC06_32566425_301	BoC06	32566425
BnC07	Ol10-D03D	26.8			
BnC07	BGO169	29.2	BoC06_34086182_244	BoC06	34086182
BnC07	bna4313	30.4	BrA06_21862527_241		
BnC07	bna4315	30.4	BoC06_34137121_183	BoC06	34137121
BnC07	BEN119	50.6	BrA06_18641591_209		
BnC07	BEN206	60.4	BrA06_21505620_333		
BnC07	bna4344	68.6	BoC06_37800109_301	BoC06	37800109
BnC07	bna4346	68.6	BoC04_31242317_293		
BnC07	bna4377	71.9	BoC06_39441907_301	BoC06	39441907
BnC07	bna4376	71.9	BoC06_39464119_301	BoC06	39464119
BnC07	bna4399	74.9	BoC06_43166079_281	BoC06	43166079
BnC07	bna4394	74.9	BoC06_43427426_301	BoC06	43427426
BnC07	bna4393	74.9	BoC06_43445980_301	BoC06	43445980
BnC07	bna4390	75.2	BoC06_43509340_301	BoC06	43509340
BnC07	bna4388	75.2	BoC06_43624619_301	BoC06	43624619
BnC07	bna4385	75.8	BoC06_43770397_301	BoC06	43770397
BnC07	bna4384	75.8	BoC06_43790514_301	BoC06	43790514
BnC07	bna3405	76.7	BoC06_44323786_278	BoC06	44323786
BnC07	bna3404	76.7	BoC06_44356546_277	BoC06	44356546
BnC07	bna3403	76.7	BoC06_44410862_275	BoC06	44410862
BnC07	bna3402	76.7	BoC06_44427102_264	BoC06	44427102
BnC07	bna3395	76.7	BoC06_44570444_256	BoC06	44570444
BnC07	BoGMS373	77			
BnC08	bna4401	0	BoC08_1857108_292		

BnC08	bna0848	1.4	BoC04_1469322_301		
BnC08	BEN69	5	BoC08_8369969_220	BoC08	8369969
BnC08	BoGMS351	8.3			
BnC08	BGO178	8.3	BoC08_18707834_252		
BnC08	BoGMS38	8.3	BrA08_4023460_168		
BnC08	bna3766	10.6	BoC08_10732119_233	BoC08	10732119
BnC08	bna3765	10.6	BoC08_10784717_302	BoC08	10784717
BnC08	bna3764	10.6	BoC08_10796441_269	BoC08	10796441
BnC08	bna4711	10.6	BoC08_10837538_302	BoC08	10837538
BnC08	sS2331BA	12.3	BoC08_10903912_116	BoC08	10903912
BnC08	bna3762	12.9	BoC08_10999144_301	BoC08	10999144
BnC08	bna3761	12.9	BoC08_11122588_301	BoC08	11122588
BnC08	bna3767	14	BoC08_11203620_301	BoC08	11203620
BnC08	bna0141	14	BoC08_11329429_300	BoC08	11329429
BnC08	BGR1B	14.6	BoC08_11779145_169	BoC08	11779145
BnC08	BEN189B	14.6	BoC08_14236962_229	BoC08	14236962
BnC08	bna0435	15.2	BoC08_14452136_301	BoC08	14452136
BnC08	bna0836	15.5	BoC07_6327946_301		
BnC08	bna4404	22.9	BoC08_18448987_301	BoC08	18448987
BnC08	bna0071	22.9	BoScaffold000394_170509_304		
BnC08	bna4403	22.9	BoC08_18489803_301	BoC08	18489803
BnC08	bna0808	23.5	BoC08_18653316_301	BoC08	18653316
BnC08	BnGMS161	37.3			
BnC08	BnEMS20	43.3	BoC08_27420490_342	BoC08	27420490
BnC08	BoGMS1200	45.9			
BnC08	BEN235	45.9	BoC08_27486008_243	BoC08	27486008
BnC08	bna2853	46.2	BoScaffold000220_330745_301		
BnC08	bna5803	55.6	BoC08_33288852_301	BoC08	33288852
BnC08	bna4522	55.6	BoC08_33288852_301	BoC08	33288852
BnC08	bna4523	55.6	BoC08_33309801_301	BoC08	33309801
BnC08	bna4524	55.6	BoC08_33323382_301	BoC08	33323382
BnC08	bna5804	55.6	BoC08_33347565_301	BoC08	33347565
BnC08	bna4525	55.6	BoC08_33349656_301	BoC08	33349656
BnC08	BGO185	57	BoC08_33413955_175	BoC08	33413955
BnC08	bna4466	57.6	BoC08_33414439_301	BoC08	33414439
BnC08	bna5790	58.2	BoC08_33626856_301	BoC08	33626856
BnC08	bna4470	58.2	BoC08_33627426_309	BoC08	33627426
BnC08	bna0149	58.2	BoC08_33685221_301	BoC08	33685221
BnC08	bna4737	59.9	BoC08_34090345_302	BoC08	34090345
BnC08	bna5319	59.9	BoC08_34149820_297	BoC08	34149820
BnC08	bna4478	59.9	BoC08_34223188_172	BoC08	34223188
BnC08	bna4479	59.9	BoC08_34299811_301	BoC08	34299811
BnC08	bna4480	59.9	BoC08_34330172_271	BoC08	34330172
BnC08	bna4483	60.5	BoC08_34447669_301	BoC08	34447669
BnC08	bna4484	60.5	BoC08_34490256_301	BoC08	34490256
BnC08	bna5795	60.8	BoC08_34507781_302	BoC08	34507781
BnC08	bna4485	60.8	BoC08_34515885_301	BoC08	34515885

BnC08	bna4486	60.8	BoC08_34516979_301	BoC08	34516979
BnC08	BGO183	64.7	BoC08_34682365_236	BoC08	34682365
BnC08	bna4488	68.6	BoC08_34885790_300	BoC08	34885790
BnC08	bna4490	68.6	BoC08_34974685_301	BoC08	34974685
BnC08	bna4496	68.6	BoC08_35193805_301	BoC08	35193805
BnC08	bna4497	68.6	BoC08_35216428_299	BoC08	35216428
BnC08	bna4498	68.6	BoC08_35252325_276	BoC08	35252325
BnC08	bna4499	68.6	BoC08_35278607_303	BoC08	35278607
BnC08	BEN52	68.9	BoC08_35597022_161	BoC08	35597022
BnC08	bna0480	75.6	BoC08_36969549_301	BoC08	36969549
BnC08	BEN82	76	BoC08_37025192_150	BoC08	37025192
BnC08	bna5810	76.8	BoC08_38521325_301	BoC08	38521325
BnC08	bna2602	76.8	BrA09_21897067_311		
BnC08	bna4543	76.8	BoC08_38528116_301	BoC08	38528116
BnC08	bna4547	76.8	BoC08_38663751_301	BoC08	38663751
BnC08	bna4551	76.8	BoC08_38792031_300	BoC08	38792031
BnC08	bna5692	76.8	BrA09_21897067_311		
BnC08	bna4554	76.8	BoC08_38862591_301	BoC08	38862591
BnC08	bna0059	76.8	BoC08_38862591_301	BoC08	38862591
BnC08	bna5179	77.4	BoC08_38982814_301	BoC08	38982814
BnC08	CB10028	77.7	BoC08_39012255_177	BoC08	39012255
BnC08	BoGMS1558	78.3			
BnC08	bna5813	79.4	BoC08_39218901_301	BoC08	39218901
BnC08	bna4558	79.4	BoC08_39223830_309	BoC08	39223830
BnC08	bna2785	79.7	BrA09_35647627_301		
BnC08	bna5480	79.7	BoScaffold000330_47398_301		
BnC08	bna5808	79.7	BoScaffold000330_198207_301		
BnC08	bna4538	79.7	BoC08_40291476_301	BoC08	40291476
BnC08	bna5807	79.7	BoC08_40306294_301	BoC08	40306294
BnC08	bna5720	79.7	BrA09_36111852_301		
BnC08	bna2782	79.7	BrA09_36016899_303		
BnC08	bna4537	79.7	BoC08_40327538_301	BoC08	40327538
BnC08	bna2783	79.7	BrA09_35751967_301		
BnC08	bna4540	79.7	BoScaffold000330_49205_301		
BnC08	bna4534	79.7	BoC08_40629883_301	BoC08	40629883
BnC08	bna4539	79.7	BoScaffold000330_354434_231		
BnC08	BnEMS860	81.1	BoC08_40829449_328	BoC08	40829449
BnC08	BEN316	83.7			
BnC08	BoGMS125	90.7	BoC08_40929085_506	BoC08	40929085
BnC08	BrGMS394	94.2	BrA09_35835741_166		
BnC08	BrGMS375C	95.7	BrA09_36262449_164		
BnC08	bna5806	96.3	BoC08_40937947_301	BoC08	40937947
BnC08	bna4531	96.3	BoC08_40937947_301	BoC08	40937947
BnC08	bna5428	98	BoC08_41331400_301	BoC08	41331400
BnC08	bna4527	98	BoC08_41383351_301	BoC08	41383351
BnC08	BoGMS1166	98.6			
BnC08	bna4795	102.5	BoScaffold000330_36569_301		

BnC08	BEN136B	104	BoC08_41173283_131	BoC08	41173283
BnC08	bna5805	115.4	BoC08_41421132_301	BoC08	41421132
BnC08	bna4526	116	BoC08_41433239_301	BoC08	41433239
BnC08	BEN136A	116.9	BoC08_41173283_131	BoC08	41173283
BnC08	BrGMS375B	122.1	BrA09_36262449_164	BrA09	36262449
BnC08	BrGMS375A	125.4	BrA09_36262449_164	BrA09	36262449
BnC08	bna2772	126.8	BrA09_36860095_301	BrA09	36860095
BnC08	bna2784	127.7	BrA09_35707655_301	BrA09	35707655
BnC08	bna2789	129.7	BrA09_35389195_301	BrA09	35389195
BnC08	bna2790	129.7	BrA09_35321451_301	BrA09	35321451
BnC08	bna2791	130	BrA09_35278168_284	BrA09	35278168
BnC08	bna5099	130	BrA09_35278168_284	BrA09	35278168
BnC08	bna2796	133	BrA09_33398631_301	BrA09	33398631
BnC08	bna2799	135	BrA09_32229790_301	BrA09	32229790
BnC08	bna5427	135	BoC08_35949347_304		
BnC08	bna5723	135.3	BrA09_32177679_301	BrA09	32177679
BnC08	bna4491	135.9	BoC08_34986249_301		
BnC08	bna2939	136.2	BrA09_31788228_301	BrA09	31788228
BnC08	bna4493	136.2	BoC08_35073478_301		
BnC08	BoGMS1382	137.6			
BnC08	BnEMS14	137.9	BoC08_36424147_266		
BnC08	BoGMS1308	139			
BnC08	BoGMS586	140.2	BoC08_34092882_250		
BnC08	BrGMS5	143.7	BrA09_30466050_255	BrA09	30466050
BnC08	bna5724	144.6	BrA09_30571654_300	BrA09	30571654
BnC08	bna4521	144.6	BoC08_33259513_301		
BnC09	bna1478	0	BrA02_23454785_301		
BnC09	bna5851	3.6	BoC09_171077_274	BoC09	171077
BnC09	BEN332C	4.7	BoC09_186601_237	BoC09	186601
BnC09	BGO199B	8.6	BoC09_193617_190	BoC09	193617
BnC09	BoGMS1072	15.7			
BnC09	BGO199A	19.3	BoC09_193617_190	BoC09	193617
BnC09	BEN343	20.7	BoC09_30393262_235		
BnC09	bna5746	21.8	BrA09_696711_301		
BnC09	bna4581	22.9	BoC09_2014248_302	BoC09	2014248
BnC09	bna4586	22.9	BoC09_1934744_301	BoC09	1934744
BnC09	bna4579	23.5	BoC09_2055747_260	BoC09	2055747
BnC09	bna5960	23.5	BoC09_2063866_301	BoC09	2063866
BnC09	bna3638	23.8	BoC09_2186157_301	BoC09	2186157
BnC09	BoGMS1484	25.2			
BnC09	BrGMS725B	26.3	BrA09_2302831_166		
BnC09	BEN55A	26.9	BoC09_2708553_177	BoC09	2708553
BnC09	FITO135B	27.2			
BnC09	FITO136	30.4			
BnC09	bna4585	40	BoC09_1990112_301		
BnC09	BnEMS820B	73.8			
BnC09	bna4592	75.5	BoC09_5057885_301	BoC09	5057885

BnC09	bna4591	75.5	BoC09_5058499_301	BoC09	5058499
BnC09	bna4761	81.5	BoC09_6686378_301	BoC09	6686378
BnC09	BnGMS213A	81.5	BrA09_13917079_149		
BnC09	BGO196	83.2	BoC09_6732672_119	BoC09	6732672
BnC09	bna0971	84.9	BoC09_7208308_301	BoC09	7208308
BnC09	bna4601	84.9	BoScaffold000035_P2_283375_259		
BnC09	bna4602	85.2	BoScaffold000035_P2_669076_301		
BnC09	bna3857	85.5	BoScaffold000035_P2_1472078_301		
BnC09	bna1011	85.5	BoC07_13141450_301		
BnC09	Na10-C01C	85.5	BoC09_8400120_250	BoC09	8400120
BnC09	BEN203	87.2	BoC09_18992253_333	BoC09	18992253
BnC09	bna4594	93.9	BoC09_23062819_301	BoC09	23062819
BnC09	bna0875	99.2	BoC09_34857697_301	BoC09	34857697
BnC09	bna4041	108.6	BoScaffold000161_P1_535291_229		
BnC09	bna0166	116.3	BrA07_13942212_301		
BnC09	bna2881	119.7	BoC07_33651629_305		
BnC09	bna1399	122	BrA02_8955295_301		
BnC09	bna3506	122	BoScaffold000001_P2_811933_302		
BnC09	bna3005	124	BrA10_14402659_301		
BnC09	bna0339	126.6	BrA08_8334365_302		
BnC09	bna3026	130.2	BrA10_13332063_292		
BnC09	bna5261	132.2	BrA10_13332063_292		
BnC09	bna0491	133.1	BoC07_10188434_304		
BnC09	bna1800	134.8	BrA03_712458_302		
BnC09	bna1436	135.9	BrA08_14011236_307		
BnC09	bna4442	136.8	BoC08_30597656_301		
BnC09	bna4265	142.4	BoC06_2517867_301		
BnC09	bna2801	147	BrA09_30763855_297		
BnC09	bna1529	148.4	BrA03_29998397_301		
BnC09	bna0944	149.5	BoScaffold000269_459624_300		
BnC09	bna3579	149.8	BoC03_4310209_301		
BnC09	bna1048	149.8	BoC08_3936744_301	BoC08	3936744
BnC09	bna0409	149.8	BoC08_4387772_301	BoC08	4387772
BnC09	bna4216	149.8	BoC08_6008129_301	BoC08	6008129
BnC09	bna4218	149.8	BoC08_6095312_301	BoC08	6095312
BnC09	bna0514	149.8	BoC06_39793715_301		
BnC09	bna0934	149.8			
BnC09	bna4220	149.8	BoC08_6163945_301	BoC08	6163945
BnC09	bna0535	149.8	BoC09_20621229_301		
BnC09	bna4221	149.8	BoC08_6286405_301	BoC08	6286405
BnC09	bna3599	150.1	BoC03_5270613_301		
BnC09	bna5849	150.7	BoC09_4095080_301		
BnC09	bna1945	151.6	BrA01_16571686_301		
BnC09	bna5399	154.2	BrA07_14137999_301		
BnC09	bna0582	159.5	BoScaffold000020_P3_180753_297		
BnC09	bna3521	167.3	BoC01_18780739_301		
	**2014**	**2020.3**	**1886**	**1374**	

[a] The method for identification of homologous collinear locus in *B. rapa* and *B. oleracea* is described by Cai *et al.* [29].

[b] The left, middle, and right refers the chromosome or scaffold of the *B. rapa* or *B. oleracea*, the physical position of the homologous locus in *B. rapa* (http://brassicadb.org/brad/index.php; chromosome_v1.5) or *B. oleracea* (http://www.ocri-genomics.org/bolbase/; chromosome_v1.0) chromosome or scaffold, and the matching base pairs of the homologous locus in *B. rapa* or *B. oleracea*, respectively.

[c] The colors of the homologous collinear fragments are same as to the colors of the *B. rapa* and *B. oleracea* chromosomes which described by Cai *et al.* [29].
